# Bovine Rectoanal Junction In Vitro Organ Culture Model System to Study Shiga Toxin-Producing *Escherichia coli* Adherence

**DOI:** 10.3390/microorganisms11051289

**Published:** 2023-05-15

**Authors:** Indira T. Kudva, Erika N. Biernbaum, Eric D. Cassmann, Mitchell V. Palmer

**Affiliations:** 1 Food Safety and Enteric Pathogens Research Unit, National Animal Disease Center, Agricultural Research Service, U.S. Department of Agriculture, Ames, IA 50010, USA; erika.biernbaum@usda.gov; 2 Oak Ridge Institute for Science and Education, Oak Ridge, TN 37830, USA; 3 Virus and Prion Research Unit, National Animal Disease Center, Agricultural Research Service, U.S. Department of Agriculture, Ames, IA 50010, USA; eric.cassmann@usda.gov; 4 Infectious Bacterial Diseases Research Unit, National Animal Disease Center, Agricultural Research Service, U.S. Department of Agriculture, Ames, IA 50010, USA; mitchell.palmer@usda.gov

**Keywords:** STEC, O157, RAJ, IVOC, adherence

## Abstract

Studies evaluating the interactions between Shiga toxin-producing *Escherichia coli* O157:H7 (O157) and the bovine recto–anal junction (RAJ) have been limited to either in vitro analyses of bacteria, cells, or nucleic acids at the RAJ, providing limited information. Alternatively, expensive in vivo studies in animals have been conducted. Therefore, our objective was to develop a comprehensive in vitro organ culture system of the RAJ (RAJ-IVOC) that accurately represents all cell types present in the RAJ. This system would enable studies that yield results similar to those observed in vivo. Pieces of RAJ tissue, obtained from unrelated cattle necropsies, were assembled and subjected to various tests in order to determine the optimal conditions for assaying bacterial adherence in a viable IVOC. O157 strain EDL933 and *E. coli* K12 with known adherence differences were used to standardize the RAJ-IVOC adherence assay. Tissue integrity was assessed using cell viability, structural cell markers, and histopathology, while the adherence of bacteria was evaluated via microscopy and culture methods. DNA fingerprinting verified the recovered bacteria against the inoculum. When the RAJ-IVOC was assembled in Dulbecco’s Modified Eagle Medium, maintained at a temperature of 39 °C with 5% CO_2_ and gentle shaking for a duration of 3–4 h, it successfully preserved tissue integrity and reproduced the expected adherence phenotype of the bacteria being tested. The RAJ-IVOC model system provides a convenient method to pre-screen multiple bacteria-RAJ interactions prior to in vivo experiments, thereby reducing animal usage.

## 1. Introduction

Shiga toxin-producing *Escherichia coli* (STEC) O157:H7 (O157) was initially discovered in 1982 after an outbreak linked to contaminated hamburgers in the United States. This outbreak affected at least 47 people and presented with symptoms including severe abdominal cramping, hemorrhagic colitis, and, notably, little to no fever [1,2]. Since then, STEC serogroups have been implicated in 265,000 infections annually in the United States, with the serogroup O157 causing 36% of these cases [3,4], and 2.8 million cases globally [5]. Non-O157 STEC are commonly implicated in human illness, including serogroups O26, O45, O103, O111, O121, and O145. These serogroups, often referred to as the “Big Six,” have been classified as food adulterants along with O157 by the USDA-Food Safety and Inspection Service (FSIS). This designation enables targeted monitoring of these pathogens within the food chain [6]. STEC infections are acquired via the fecal-oral route by contacting infected humans or animals and/or consuming infected food and water [7]. Infections in humans can manifest either asymptomatically or with a range of symptoms, from watery to bloody diarrhea, which can often worsen and lead to secondary complications such as hemorrhagic uremic syndrome (HUS), resulting in kidney failure, or thrombotic thrombocytopenic purpura (TTP), causing systemic organ failure [8,9].

Cattle serve as the primary reservoirs for STEC. Unlike humans, they remain asymptomatic due to the absence of globotriaosylceramide/Gb_3_ receptors along their gastrointestinal tract, which are essential for the uptake of Shiga toxins [10,11]. Although STEC can colonize various sites within the bovine gastrointestinal tract, a notable tendency for persistence has been observed at the RAJ [12,13,14]. The RAJ is located at the terminal end of the ruminant gastrointestinal tract, where the columnar epithelial cells in the distal colon transition to the stratified squamous epithelial cells towards the anus [12,13,15,16]. Colonization at the RAJ may serve as a prerequisite for super-shedding cattle, which intermittently shed greater than 10^4^ CFU/g of O157 in their feces. This phenomenon contributes to increased O157 transmission within herds [17] and contamination of the environment [18]. O157 tropism for the RAJ can result in micro-colony formation, especially in a region 3–5 cm proximal to the RAJ, thus providing the greatest impact on O157 load in feces [12,14]. The region is proximal to the RAJ and comprises dense lymphoid follicles [19], covered by columnar epithelial cells referred to as the follicle-associated epithelium (FAE). In contrast, O157 exhibits diffuse adherence to the remaining stratified squamous epithelial cells at the RAJ, which is also known as the rectoanal junction squamous epithelium (RSE) [20]. Other bacteria attach to the RAJ, including commensal *E. coli*, and RAJ bacterial communities can shift with O157 colonization [21,22]. However, STEC employs different strategies for adherence to the RAJ compared to commensal bacteria. The O157 proteins, which are responsible for attachment to FAE, are distinct from those required for RSE adherence and also differ from other STEC strains [20,23,24,25,26,27].

Given these observations, blocking STEC adherence at the RAJ is a potential strategy for pre-harvest reduction, providing an impetus for studies involving this site of persistence in cattle. Numerous studies, both in vitro and in vivo, have been conducted utilizing the RAJ to better understand the dynamics between the site and STEC, especially serogroup O157, in cattle [28,29,30,31,32]. Although these studies have provided some insights into O157 and host proteins contributing to O157 persistence, additional studies are needed to determine factors associated with differences in adherence between the cell types at the RAJ and between different O157 strains as well as STEC serogroups [20,23,24,25,26]. Currently, there is an increased reliance on expensive animals to conduct such studies due to the lack of a convenient in vitro model system closely representing both cell types at the RAJ and demonstrating STEC adherence phenotypes observed in vivo. The bovine RSE cell-based adherence assay, which has been standardized by our research team, enables in vitro studies. However, this assay is limited to studying only one cell type at the RAJ [20,23,24,25,26]. Hence, we decided to develop a RAJ in vitro organ culture (RAJ-IVOC) model system that incorporates both RSE and FAE cells. This model system, when optimized, would permit bacterial adherence studies without loss of tissue integrity. Furthermore, it aims to produce adherence results that closely resemble those observed in in vivo animal studies and the in vitro RSE-cell assay. The development of such a comprehensive in vitro model would bring about two significant advantages. Firstly, it would enable the evaluation of multiple bacteria for adherence, allowing for the selection of relevant strains shortlisted for final in vivo studies. Secondly, it would facilitate the rapid screening of various adherence-inhibiting therapies before their application, thereby reducing the overall need for animal usage.

## 2. Materials and Methods

Bacterial Strains. The IVOC assay was standardized using the following bacterial strain: (i) O157 strain EDL933 (ATCC 43895: *stx*1^+^, *stx*2^+^, *eaeA*^+^, *hlyA*^+^) (American Type Culture Collection/ATCC, Manassas, VA, USA), referred to as “O157.” This particular O157 strain was selected based on the previously reported aggregative, moderate adherence phenotype on RSE cells [20,33]. (ii) Non-STEC *E. coli* K12 (MG1655/ATCC 700926: F-lambda-*ilvG*-*rfb*-50 *rph*-1, *stx*1^−^, *stx*2^−^, *eaeA*^−^, *hlyA*^−^), referred to as “*E. coli* K12”.

Bacterial Inoculum Preparation. As previously described, all bacteria were grown overnight in Dulbecco’s modified Eagle’s medium with low glucose (DMEM-LG; Invitrogen, Carlsbad, CA, USA) at 37 °C without aeration, washed, and re-suspended in DMEM with no glucose (DMEM-NG; Invitrogen) before testing in the adherence assays described below [20,33,34].

RAJ tissue collection and processing. RAJ tissues were collected at necropsies of animals being utilized in other studies at the National Animal Disease Center (NADC, Ames, IA, USA), under the approval of the NADC Institutional Animal Care and Use Committee (Supplementary Table S1). Animals included Holstein, Jersey, or Dexter cows, 3–9 years of age. They were fed a maintenance diet with ad libitum access to water. Tissue samples were collected and transported in a transport medium composed of Dulbecco’s modified Eagle’s medium (no glucose) (DMEM-NG; Invitrogen). The transport medium was supplemented with 2.5% fetal bovine serum (FBS; Thermo Scientific HyClone, Logan, UT, USA), 100 µg/mL streptomycin, 100 U/mL penicillin (Pen-Strep; Invitrogen), 2.5 mg/L amphotericin B (Sigma), and 50 µg/ ml gentamicin (Invitrogen). The tissues were kept on ice during transportation. Upon arrival at the laboratory, the tissue samples were rinsed with cold phosphate-buffered saline (PBS) to remove fecal material. Subsequently, they were placed in DMEM-NG with 2.5% FBS while the underlying muscularis layer was trimmed. The rinsed and trimmed tissue was placed in a fresh transport medium on ice for 1 h. It was then either processed for RSE cell extraction, as previously described [20], or used for setting up IVOCs, as described below.

**Figure 1:**
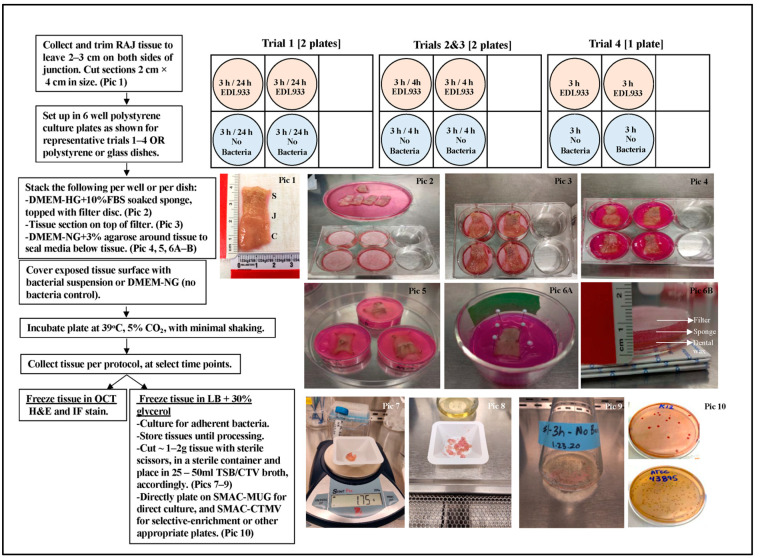
Bovine RAJ- IVOC set up and processing protocol summary.

RAJ-IVOC assembly. The trimmed RAJ tissue was rinsed in PBS and cut into multiple small rectangular pieces, following sterile precautions, as shown in Figure 1. Each piece measured approximately 2 cm × 4 cm (length × breadth) in size. This ensured that about 2 cm of the region on either side of the RAJ was included in each piece of tissue (Figure 1, Pic. 1). Throughout the process, the tissue pieces were kept moist in DMEM-NG. Wells of a 6-well, flat-bottomed polystyrene clear tissue culture plates or individual polystyrene or glass dishes (Figure 1, Pic. 2–6; Corning/Costar, Sigma-Aldrich Corp., St. Louis, MO, USA) were prepared by layering the bottom with a 1–2 mm thick sterile sponge soaked in DMEM-High Glucose (Invitrogen) with 10% FBS. On top of each sponge, a sterile Whatman filter disc (grade 1, 32 mm; Sigma) was placed, as shown in Figure 1. A single tissue piece was laid on top of the filter disc (without stretching) per well with the mucosal (luminal) surface facing upwards, taking care to handle the tissue only at the edges. When needed, sterile pins were used to secure the tissue along the edges (Figure 1, Pic. 6A). In addition, a strip of sterile dental wax (Polysciences, Inc., Warrington, PA, USA) was inserted beneath the sponge to serve as a surface for tethering the pins (Figure 1, Pic. 6B). Sterile DMEM-NG with 3% agarose was then applied around the tissue to generate a seal between the media below and the edges of the tissue on top (Figure 1). This allowed the mucosal region of the RAJ-IVOC to be exposed for evaluation of bacterial adherence phenotypes and contained the bacterial inoculum on the mucosal surface (Figure 1). Considering that the average RAJ-IVOC mucosal surface area is 8 cm^2^, of which the average exposed squamous epithelial cell surface area is 20 µm across, the average exposed columnar epithelial cell surface area is 6 µm across, and there is a close to equal distribution of the two cell types, a total of 10^4^ cells were estimated to comprise the exposed surface area of the RAJ-IVOC. This estimation was used to determine the range of bacterial inoculum to be tested in the RAJ-IVOC adherence assay.

RAJ-IVOC adherence standardization. Due to the limitation in RAJ tissue availability, only the final comparative assays were conducted with technical replicates per strain. However, the “no bacteria” controls were included in all trials and comparative assays for each bacterium tested (Figure 1). The O157 strain EDL933 was evaluated in all assays, while the non-STEC *E. coli* K12 was included only in the comparative assays for verification of results. To standardize the adherence assay, the following conditions were tested in trials 1–4 before the final comparative assays: (i) Incubation time. The RAJ-IVOC-containing plates were incubated for 24, 4, or 3 h (Figure 1). Different incubation times were used to determine the optimal time within which adherence results could be obtained with minimal or no damage to the IVOC system or tissue integrity. (ii) Inoculum concentration. The initial inoculum concentration was set at 100 bacteria per cell (100:1 ratio). A total of 10^6^ CFU bacteria were used to inoculate the estimated 10^4^ exposed tissue surface cells when standardizing the RAJ-IVOC system in the initial trials 1–3. After establishing the optimal incubation time for RAJ-IVOC, the inoculum concentration was increased to 10^8^ CFU bacteria (10,000:1 ratio) for trial 4 and the first round of comparative assays. This increase in concentration aimed to facilitate the visualization of bacteria and determine the specific role of O157 strain ELD933, in the observed histopathology. Subsequently, comparative assays were conducted to evaluate the bacteria:cell ratios of 10^7^ bacteria (1000:1 ratio) and 10^6^ bacteria (100:1 ratio). The aim was to determine the optimal inoculum that would reproduce the adherence phenotypes observed in vivo in animals and in the in vitro RSE-cell assay [20,23,24,26,35]. Adherence patterns on RAJ-IVOC cells were qualitatively recorded as diffuse, aggregative, or nonadherent.

As shown in Figure 1, the bacterial inoculum prepared in DMEM-NG (2 mL total volume) was added to cover the entire top surface of the RAJ-IVOC tissue. For no-bacterial controls, DMEM-NG without bacteria was used in place of the inoculum. Post-inoculation, all RAJ-IVOC-containing plates were incubated at 39 °C with 5% CO_2_ and gentle shaking at 100–110 rpm. After incubation, the inoculum or media left on top of the tissue surface for each IVOC setup per plate/dish was separately aspirated. These aspirated samples were then plated on sorbitol MacConkey agar (BD Biosciences) containing 4-methylumbelliferyl-β-d-glucuronide (100 mg/liter; Sigma) (SMAC-MUG) and MacConkey agar (BD Biosciences) containing 4-methylumbelliferyl-β-d-glucuronide (100 mg/liter; Sigma) (MAC-MUG). The purpose of these platings was to isolate O157 and *E. coli* K12 or other background bacteria, respectively. Each RAJ-IVOC tissue was gently disengaged from the agarose, rinsed with DMEM-NG, and cut in half using sterile precautions. One-half of the RAJ-IVOC tissue, weighing about 1–2 g, was frozen in 10 mL LB with 30% glycerol until ready to culture as described below. The other half of the RAJ-IVOC tissue was placed in an optimal cutting temperature solution (OCT; Tissue-Tek, Sakura Finetek, Torrance, CA, USA) and flash frozen in isopentane on liquid nitrogen before storing at −80 °C. In the laboratory, the frozen tissue was sectioned using the Leica CM 1900 cryostat (Leica Microsystems, Buffalo Grove, IL, USA). The resulting sections, with a thickness of 5 µm, were collected on Colorfrost slides (Thermo Fisher Scientific), air-dried, and then fixed in 95% ethanol. Subsequently, the fixed sections were sent to Microscopy Services at NADC for hematoxylin and eosin (H&E) staining, which served as a histopathological screening method. Alternatively, immunofluorescent staining was performed according to the procedure described below to study the presence and distribution of the inoculated bacteria and tissue markers. All H&E-stained slides were scanned using the Aperio Digital Pathology solutions (Aperio LV1, Leica Biosystems, Deer Park Illinois) to generate electronic images for analysis. The Aperio Image Scope software (eSlideManager version 12.5.0.645; Leica) was used to analyze and examine these images.

RAJ-IVOC tissue culture. Previously, standardized non-enrichment and selective-enrichment protocols [36,37,38] were used to culture the RAJ-IVOC tissue. Briefly, approximately 1–2 g of the frozen RAJ-IVOC tissue sample was thawed and minced under sterile precautions. The minced tissue was then added to 25–50 mL of Trypticase soy broth (BD Bioscience, San Jose, CA, USA), which was supplemented with cefixime (50 μg/L; U.S. Pharmacopeia, Washington, DC, USA), potassium tellurite (2.5 mg/L; Sigma), and vancomycin (40 mg/L; Alfa Aesar, Haverhill, MA, USA) (TSB-CTV). The mixture was thoroughly mixed. The TSB-CTV suspension was serially diluted using sterile saline (0.15 M NaCl) both before and after overnight incubation at 37 °C with aeration. The dilutions prepared before incubation were spread on SMAC-MUG (non-enrichment culture). The dilutions prepared after overnight incubation were plated on SMAC-MUG supplemented with cefixime (50 μg/L), potassium tellurite (2.5 mg/L), and vancomycin (40 mg/L) (SMAC-CTMV; selective-enrichment culture). After overnight incubation at 37 °C, both SMAC-MUG and SMAC-CTMV plates were examined. Colonies that did not ferment sorbitol or exhibit fluorescence under UV light in the presence of 4-methylumbelliferyl-β-d-glucuronide were further confirmed as O157 using latex agglutination tests (*E. coli* O157 latex, Oxoid Diagnostic Reagents, Oxoid Ltd., Hampshire, UK). The non-enriched and selective-enriched cultures were additionally plated on MAC-MUG for increased recovery of any background non-STEC bacteria besides the lactose-fermenting, MUG-utilizing (fluorescent under UV light) *E. coli* K12.

Bacteria recovered from the RAJ-IVOC tissue cultures were typed using polymorphic amplified typing sequences (PATS) to determine the DNA fingerprint and verify against the inoculated O157 and *E. coli* K12 strains, as described below. Quantitative data obtained from the comparative RAJ-IVOC tissue cultures were subjected to statistical analysis to determine the significance of any difference in adherence. The unpaired t-test or one-way ANOVA with Dunnett’s test was employed for this purpose. A significance level of *p* < 0.05 was considered significant. The statistical analysis was performed using GraphPad Prism version 8.0.0, GraphPad Software, San Diego, CA, USA.

To verify the DNA fingerprint of the recovered bacteria, a technique called polymorphic amplified typing sequence (PATS) was employed. This method had been previously described in studies [39,40,41,42]. The bacterial isolates obtained from the RAJ-IVOC tissue cultures underwent PATS analysis for fingerprinting purposes. Briefly, specific primer pairs were utilized to target the 8 polymorphic *Xba*I- and 7 polymorphic *Avr*II- restriction enzyme sites, as well as 4 virulence genes encoding Shiga toxins 1 and 2 (*stx*1 and *stx*2), intimin-γ (*eaeA*), and hemolysin-A (*hlyA*), which were used to generate amplicons from the colony lysates [27,39,40,42,43]. PCR reactions amplifying the *AvrI*I- restriction enzyme site were purified (QIAquick PCR purification kit, Qiagen, Valencia, CA, USA) and digested with the *Avr*II restriction enzyme (New England Biolabs, Beverly, MA, USA) to confirm the presence of the restriction site. All reactions were electrophoresed on 3% agarose gels stained with ethidium bromide. The presence or absence of amplicons for *Xba*I and the virulence genes was recorded using “1” and “0,” respectively. The absence of an *Avr*II amplicon was recorded as “0,” and the presence of the restriction site with a small nucleotide polymorphism was recorded as “1,” “2” for an intact restriction site, and “3” for a restriction site duplication [39,40,43].

Immunofluorescent staining. (i) For test bacteria and RAJ-IVOC cells. As previously described [20,44], ethanol-fixed slides with RAJ-IVOC tissue sections were washed in PBS with 0.1% Tween20 (PBS-T; Sigma), blocked with 5% normal goat serum (NGS) (Vector Laboratories, Burlingame, CA, USA) in PBS-T (room temperature/RT for 20 min), before incubation with primary and secondary antibodies, each at RT for 1 h. Primary antibodies included the mouse anti-PAN cytokeratins (AbD Serotec, Raleigh, NC, USA) targeting the RAJ cell cytokeratins and the rabbit anti-*E. coli* (Thermo Scientific Pierce) targeting the *E. coli* K12 strain. Secondary antibodies included the Alexa Fluor 594 (red)-labeled goat anti-mouse IgG (H+L; F(ab’)2 fragment) (Invitrogen) targeting the anti-cytokeratins primary antibody. Alexa Fluor 488 (green) labeled goat anti-rabbit IgG (H + L; F (ab’)2 fragment) (Invitrogen) targeting the anti-*E. coli* primary antibody or the fluorescein isothiocyanate (FITC; green)-labeled goat anti-O157 (KPL, Gaithersburg, MD, USA) antibodies targeting O157. Post-washing with PBS-T and distilled water, the slides were air-dried in the dark and cover-slipped with Prolong Gold anti-fade reagent containing the DNA stain 4′,6-diamidino-2-phenylindole (DAPI; Invitrogen). Immunofluorescent images from the stained slides were captured using the Nikon Eclipse E800 fluorescence microscope (Nikon Instruments Inc., Elgin, IL, USA) with appropriate filters and digital imaging capabilities [20,44]. Control slides with no bacteria were stained with the same set of antibodies to rule out nonspecific binding. Additional controls were prepared by staining test slides with FITC-tagged antibodies targeting unrelated *Salmonella* bacteria to demonstrate the specificity of the antibodies used [20,44].

(ii) For RAJ-IVOC tissue markers. Cell structural, adhesion, and junctional protein complexes are abundant in epithelial tissues and, hence, can be used as markers of tissue integrity [45]. The staining and cover slipping protocol described above for bacteria and cells was used for staining RAJ-IVOC tissue sections for specific tissue markers, epithelial cadherin (E-cadherin), neural cadherin (N-cadherin), and vimentin, with a different set of primary–secondary antibodies. Cadherins are transmembrane glycoproteins involved in cell-cell adhesion and maintaining tissue integrity [46]. To target cadherins, the primary antibodies used were rat anti-E-cadherin/eukaryotic transmembrane glycoprotein (Novus Biologicals, Littleton, CO, USA) and rabbit anti-N-cadherin/eukaryotic transmembrane glycoprotein (Abcam, Cambridge, MA, USA). The secondary antibodies employed were Alexa Fluor 594 (red) labeled goat anti-rat IgG (H + L; F (ab’)2 fragment) or Alexa Fluor 488 (green) labeled goat anti-rabbit IgG (H + L; F (ab’)2 fragment) (Invitrogen) [44].

Vimentin is an embryonic cytoskeleton filament protein involved in the intracellular transport of proteins that continues to be expressed in adult animals by fibroblasts lining the submucosa [47,48]. To target vimentin, the mouse anti-vimentin/eukaryotic mesenchymal cell protein (Abcam) was used as the primary antibody and the Alexa Fluor 488 (green) labeled goat anti-mouse IgG (H + L; F (ab’)2 fragment) (Invitrogen) as the secondary antibody [44].

In addition to the above tissue markers, the less abundant tight junction protein, occludin, was also targeted since it appears to play an important role in regulating tight junctions [49]. To target occludin, mouse anti-occludin (Invitrogen) was used as the primary antibody, and the Alex Fluor 594 (red) labeled goat anti-mouse IgG (H + L; F (ab’)2 fragment) (Invitrogen) was used as the secondary antibody. While most of the staining protocol was executed at room temperature, the occludin-targeting primary antibody required overnight incubation at 4 °C for improved detection.

RAJ-IVOC tissue viability test. The viability of the RAJ-IVOC tissues, used to set up the comparative assays, was evaluated. Two uninoculated and unfixed tissue samples were tested per assay. One sample was tested pre-incubation, and the other post-incubation to determine if the tissue collection process or test conditions would increase tissue necrosis, leading to decreased cell viability in the RAJ-IVOC. The RedDot2 nuclear staining dye (RedDot™2 Far-Red Nuclear Stain, Biotium, Inc., Fremont, CA, USA) was used per the manufacturer’s instructions; viable cells with intact membranes remain impermeable to the RedDot2 reagent, and hence the nuclei within do not stain red. The RedDot2 reagent in the kit was used to stain fixed RAJ tissue in our stock, and the results were compared to sections of the same tissue stained with the commonly used DNA stain 4′,6-diamidino-2-phenylindole (DAPI; Invitrogen) to verify nuclear staining of tissues lacking intact cell membranes; DAPI stains nuclei irrespective of cellular integrity (Invitrogen). Once verified, the RedDot2 reagent was routinely used to stain two uninoculated, unfixed tissue samples per assay. Briefly, each tissue sample was soaked in RedDot2 reagent (Biotium) diluted in DMEM-NG for 30 min at room temperature before rinsing and freezing as described above under ‘RAJ-IVOC adherence standardization’. The stained frozen tissues were then sectioned on the cryostat to prepare 4–6 µm thick sections, collected on Colorfrost slides (Thermo Fisher Scientific, Pittsburgh, PA, USA), air dried, and cover-slipped with Prolong Glass anti-fade reagent (Invitrogen) before visualization by fluorescent microscopy.

RSE adherence assay. The RSE assay was conducted in two biological replicates with eight technical replicates per assay. Briefly, RSE cells were suspended in DMEM-NG to a final concentration of 10^5^ cells/mL. Each bacterial isolate was mixed with RSE cells at a bacteria:cell ratio of 10:1. The mixture was incubated at 37 °C with aeration (110 rpm) for 4 h, pelleted, washed, and reconstituted in 100 µL of double-distilled water (dH_2_O). Drops of the suspension (2 µL) were placed on Polysine slides (Thermo Scientific/Pierce, Rockford, IL, USA), dried, fixed, and stained with fluorescence-tagged antibodies specific to the O157 antigen and cytokeratins of the RSE cells as described below and previously reported [20,23,24,26,27,33,35,50]. Phase-contrast images of the same slides were recorded prior to fluorescent staining to verify the presence of RSE cells and bacteria. Adherence patterns on RSE cells were qualitatively recorded as diffuse, aggregative, or nonadherent, and quantitatively as percentages of RSE cells with or without adhering bacteria [23]. The adherence of bacteria to RSE cells was categorized based on the percentage of RSE cells with adherent bacteria. The categories were defined as follows: hyper-adherent when more than 50% of RSE cells had 10 adherent bacteria; moderately adherent when 50% or less of the RSE cells had 5 to 10 adherent bacteria; and nonadherent when less than 50% of the RSE cells had only 1 to 5 adherent bacteria. RSE cells with no added bacteria were subjected to the assay procedure and used as negative controls to confirm the absence of pre-existing O157 bacteria.

Statistical analyses. Quantitative data from the RSE adherence assay were evaluated for statistical significance using one-way ANOVA with Dunnett’s test; *p* < 0.05 was considered significant (GraphPad Prism version 8.0.0, GraphPad Software, San Diego, CA, USA).

## 3. Results

### 3.1. RAJ-IVOC Maintains Tissue Integrity and Viability Following Incubation for 3 h at 39 °C

Histopathological analysis, examination of tissue markers, and viability assessments conducted on the RAJ-IVOCs in trials 1–4 demonstrated that the optimal preservation of tissue structural integrity occurred at 3 h post-incubation at 39 °C. This time point provided better results compared to 24 h of incubation. The observed benefits were consistent regardless of whether the IVOC was assembled using polystyrene or glass dishes, as described in the “Materials and Methods: RAJ-IVOC Assembly” section (Figure 1).

#### 3.1.1. Histopathology

**Figure 2:**
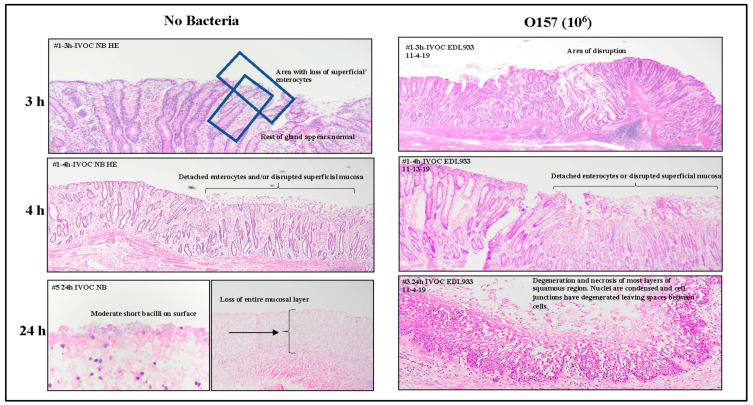
Representative histopathological reports from the adherence assay trials. The RAJ-IVOC were inoculated with either O157 (10^6^ CFU inoculum as shown in parenthesis) or not inoculated (no bacteria) and incubated at 39 °C for 3, 4, or 24 h. H&E-stained tissue section slides were scanned using the Aperio digital pathology system to obtain the eImages; observations of mucosal disruption or degenerations along with experimental conditions are noted on the images.

Sections of the OCT-frozen RAJ-IVOC collected after 3, 4, or 24 h of incubation, without any inoculum bacteria (no bacteria or NB) or with O157, were H&E stained and evaluated after each trial for histopathology (Figure 2, Supplementary Figures S1–S4, and Supplementary Histopathology Reports folder). All sections of the tissue samples exhibited clear visibility of the squamous (RSE cells) and columnar epithelial cell regions, as well as the junction between them. Additionally, lymphoid follicles were observed beneath some of the columnar epithelia, which are referred to as FAE cells or the lymphoepithelium/glandular region in certain pathology reports (Supplementary Histopathology Reports folder).

As shown in Figure 2, evaluation of the H&E-stained RAJ-IVOC sections indicated minimal to no disruption of the mucosa or cellular degeneration following 3–4 h of incubation at 39 °C, compared to 24 h. The tissue sections of both NB-RAJ-IVOC and O157-RAJ-IVOC, incubated for 3–4 h, exhibited similar appearances. They showed normal-looking squamous and columnar epithelia, with the exception of some areas in the glandular region where superficial disruption of the columnar epithelial cells closest to the lumen was observed (Figure 2). The observed foci of cellular disruption in the glandular region of the RAJ-IVOC sections extended for 5–50% of the region in trials 1–3. In some cases, the disruption extended into deeper regions of the gland and lamina propria, particularly with longer incubation times and in the O157-RAJ-IVOC samples (Figure 2, Supplementary Histopathology Reports folder). No disruption was observed in the sections from the NB-RAJ-IVOC in trial 4 after a 3 h incubation period. Both the columnar and squamous regions remained intact during these assays. However, only a few foci of superficial epithelial disruption were observed in the glandular region (Supplementary Histopathology Reports folder).

These observations were in complete contrast to the RAJ-IVOC samples that were incubated for 24 h. Regardless of the inoculum used, these samples exhibited significant signs of tissue damage, including loss of mucosa, condensed nuclei, degeneration of cellular and cell junction, diffuse full-thickness autolysis, and necrosis throughout the squamous and columnar regions (Figure 2, Supplementary Histopathology Reports folder). 

#### 3.1.2. Cell Markers

**Figure 3:**
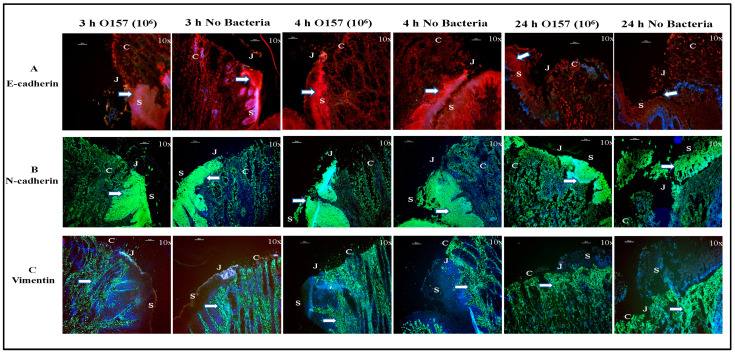
Histomorphology of the bovine RAJ-IVOC tissues from the adherence assay trials. The RAJ-IVOC were inoculated with either O157 (10^6^ CFU inoculum as shown in parenthesis) or not inoculated (no bacteria) and incubated at 39 °C for 3, 4, or 24 h. Tissue sections of the RAJ-IVOC were then stained with immunofluorescent antibodies targeting tissue markers (indicated with arrows), (**A**) E-cadherin (green), (**B**) N-cadherin (green), and (**C**) vimentin (green), and nuclei (blue). The squamous (S), junction (J), and columnar (C) regions of the RAJ are indicated along with a 100 µm scale bar. Images were captured with a 10× objective and shown at 100× magnification.

**Figure 4:**
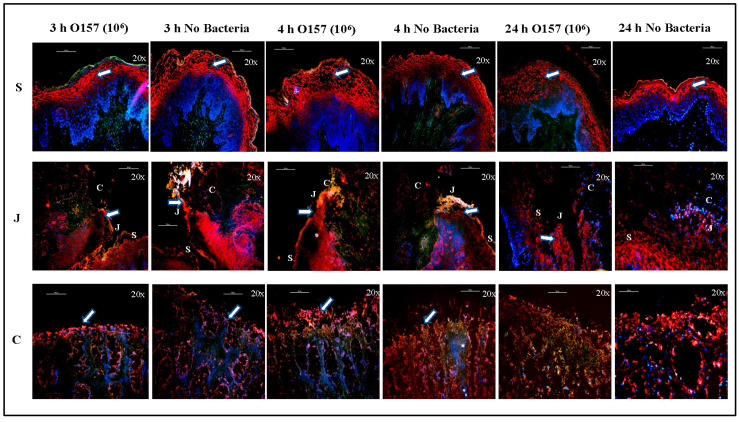
Distribution of occludin on RAJ-IVOC tissue post-3-, 4-, and 24-h incubation. The RAJ-IVOC were inoculated with either O157 (10^6^ CFU inoculum as shown in parenthesis) or not inoculated (no bacteria) and incubated at 39 °C for 3, 4, or 24 h. Tissue sections of the RAJ-IVOC were then stained with immunofluorescent antibodies targeting the tissue marker (indicated with arrows), occludin (red), and nuclei (blue). The squamous (S), junctional (J), and columnar (C) regions of the RAJ are indicated along with a 100 µm scale bar. Images were captured with a 10× objective and shown at 100× magnification.

The distribution of cell and mesenchymal markers additionally verified that the cellular architecture of the RAJ-IVOC was better maintained at 3–4 h incubation at 39 °C compared to 24 h (Figure 3 and Figure 4). After incubation for 3–4 h, as previously reported, E- and N-cadherins were identified using specific antibodies on all epithelial cells lining the RAJ-IVOC. N-cadherin was found to be more abundant in the squamous epithelial cells (Figure 3; [44]). Vimentin was identified in the fibroblasts lining the submucosa as reported in the earlier studies (Figure 3; [44,47,48]). Additionally, occludin at the tight junctions distinctly outlined both the squamous and columnar epithelia (Figure 4; [49]). However, these structural and junctional markers were loosely compartmentalized in tissue sections from RAJ-IVOC incubated for 24 h, further highlighting the extensive cellular degeneration and autolysis in the same (Figure 3 and Figure 4).

#### 3.1.3. Viability Test

**Figure 5:**
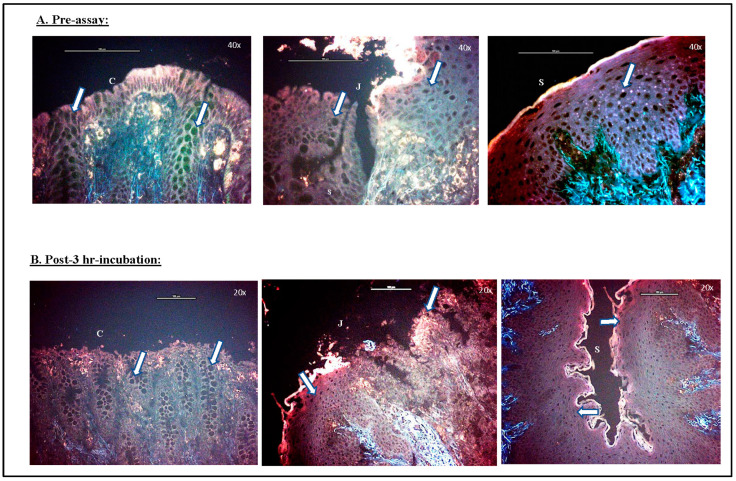
RAJ-IVOC viability test using the RedDot2 nuclei staining method. The unfixed RAJ-IVOC tissue was stained with RedDot2 dye (**A**) pre-assay and (**B**) post-3 h incubation. The absence of nuclear staining (indicated with arrows) reflects good tissue integrity and viability. Images were captured at 400–200× magnification; the objective used is shown on the images along with a 100 µm scale bar. The squamous (S), junction (J), and columnar (C) regions of the RAJ are also indicated.

The RedDot2 nuclear staining method was used to ascertain the viability of cells within the RAJ-IVOC before and after incubation for 3 h (Figure 5). This time point was selected based on the histopathology reports, the distribution of cell markers discussed above, and the bacterial adherence patterns described below. The representative images of this test, as shown in Figure 5, depict the absence of nuclear staining in the unfixed RAJ-IVOC tissues pre- and post-3 h incubation. This observation confirmed that the RAJ tissue collection method and the RAJ-IVOC setup procedures were conducive to maintaining tissue viability throughout the process.

To verify the reliability of the RedDot2 nuclear staining method, fixed RAJ tissue sections were stained with the RedDot2 dye, and sections of the same tissues were stained with the other commonly used nuclear staining dye, DAPI. As seen in Supplementary Figure S5, in tissues lacking cellular integrity, the RedDot2 dye stains nuclei red, and the nuclei can be verified with the blue DAPI-stained images (Supplementary Figure S5). 

### 3.2. O157 Produces Distinct Adherence Patterns on the RAJ-IVOC Following 3 h Incubation at 39 °C

The O157 strain EDL933 (ATCC 43895; O157) was specifically chosen for this study based on its established ability to adhere to RSE cells in a distinct aggregative pattern [20,23,24,26,27,35,51]. Initially, two different O157 concentrations were used to inoculate the RAJ-IVOC: 10^6^ CFU for trials 1–3 and 10^8^ for trial 4. Using a lower inoculum concentration in the first three trials allowed for better visualization of tissue structure while determining optimal methods and incubation times. However, to obtain clarity on the bacterial adherence phenotype on the RAJ-IVOC and subsequently standardize the adherence assay, a higher inoculum concentration was used.

**Figure 6:**
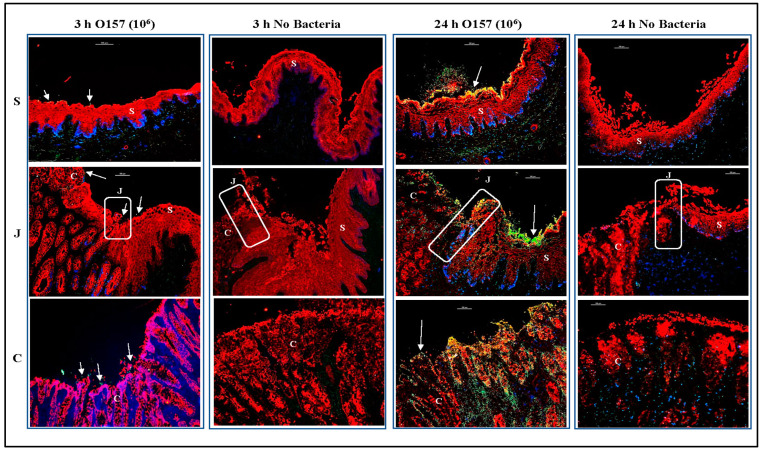
Immunofluorescent images of tissue sections from RAJ-IVOC adherence assay trial 1. The RAJ-IVOC were inoculated with either O157 (10^6^ CFU inoculum as shown in parenthesis) or not inoculated (no bacteria) and incubated at 39 °C for 3 or 24 h. Tissue sections of the RAJ-IVOC were then stained with immunofluorescent antibodies targeting the RAJ cells’ cytokeratins and O157, and images were recorded at 100× magnification. The adherent bacteria (shown with arrows), RAJ cells’ cytokeratins, and the nuclei have green, orange–red, and blue fluorescence, respectively. The squamous (S), junction (J, boxed), and columnar (C) regions of the RAJ are indicated along with a 100 µm scale bar.

**Figure 7:**
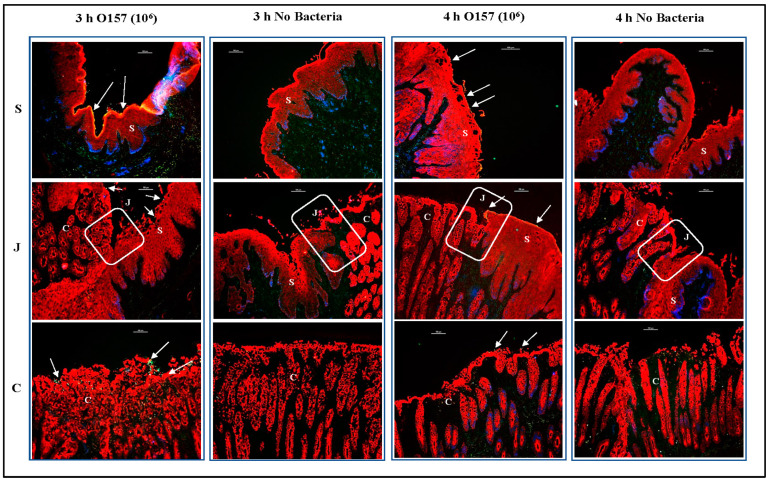
Immunofluorescent images of tissue sections from RAJ-IVOC adherence assay trials 2 and 3. The RAJ-IVOC were inoculated with either O157 (10^6^ CFU inoculum as shown in parenthesis) or not inoculated (no bacteria) and incubated at 39 °C for 3 or 4 h. Tissue sections of the RAJ-IVOC were then stained with immunofluorescent antibodies targeting the RAJ cells’ cytokeratins and O157, and images were recorded at 100× magnification. The adherent bacteria (shown with arrows), RAJ cells’ cytokeratins, and the nuclei have green, orange–red, and blue fluorescence, respectively. The squamous (S), junction (J, boxed), and columnar (C) regions of the RAJ are indicated along with a 100 µm scale bar.

**Figure 8:**
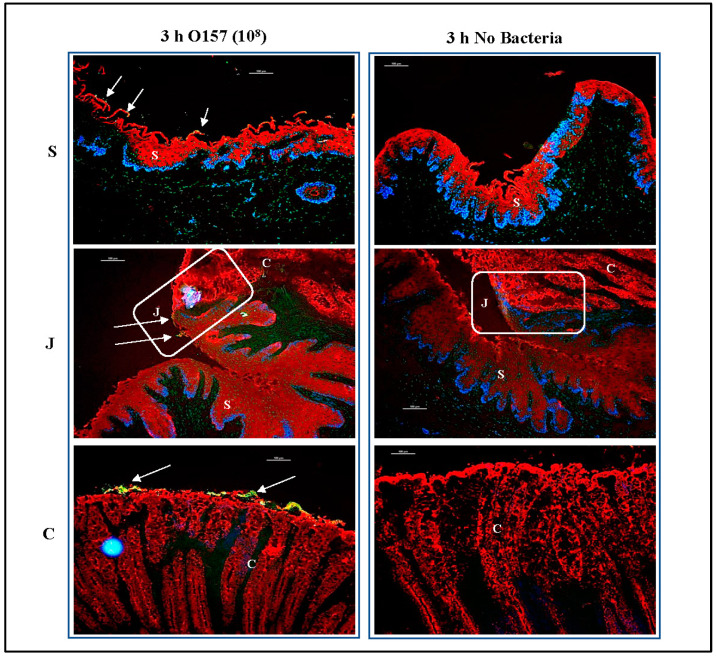
Immunofluorescent images of tissue sections from RAJ-IVOC adherence assay trial 4. The RAJ-IVOC were inoculated with either O157 (10^8^ CFU inoculum as shown in parenthesis) or not inoculated (no bacteria) and incubated at 39 °C for 3 h. Tissue sections of the RAJ-IVOC were then stained with immunofluorescent antibodies targeting the RAJ cells’ cytokeratins and O157, and images were recorded at 100× magnification. The adherent bacteria (shown with arrows), RAJ cells’ cytokeratins, and the nuclei have green, orange–red, and blue fluorescence, respectively. The squamous (S), junction (J, boxed), and columnar (C) regions of the RAJ are indicated along with a 100 µm scale bar.

Immunofluorescence imaging of tissue sections of the RAJ-IVOCs used in trials 1–4 allowed for these focused evaluations. Salient observations from the four trials, as represented in Figure 6, Figure 7 and Figure 8, can be summarized as follows: (i) Tissue degeneration (Figure 2, Supplementary Histopathology Reports) occurs after 24 h incubation, resulting in the extensive proliferation of the inoculated bacteria regardless of the initial concentration. This proliferation makes it difficult to determine the adherence phenotype (Figure 6), (ii) In contrast, after 3 or 4 h incubation, the adherence phenotype is observable and consistently reproduced at both 10^6^ CFU and 10^8^ CFU inoculum concentrations (Figure 6, Figure 7 and Figure 8). The differential adherence pattern of O157 on RAJ cells was clearly observed on the RAJ-IVOC during these incubation times. O157 exhibited adherence in small aggregates along the squamous region and formed microcolonies along the columnar epithelia, especially along the FAE cells (Figure 6, Figure 7 and Figure 8, Supplementary Figures S1–S4). Increased focal disruption of epithelia, as described under “Histopathology” above, could be associated with the presence of O157, especially when at the higher inoculum concentration of 10^8^ CFU (Figure 8, Supplementary Histopathology Reports). No O157 bacteria were detected in any of the NB-RAJ-IVOC tissues, which served as both tissue and method controls in each of the assays (Figure 6, Figure 7 and Figure 8). This result eliminates the possibility of any pre-existing or cross-contaminating O157 in the samples. Based on the adherence, histopathology, and cell marker results that validated the RAJ-IVOC assembly and assay methods, an incubation time of 3 h was selected for all subsequent RAJ-IVOC assays.

### 3.3. O157 Adherence Patterns Observed In Vitro and In Vivo Are Best Reproduced at the 10^7^ Inoculum Concentration

Comparing two bacteria with demonstrated differences in adherence to the RAJ cells was required to determine an optimal inoculum concentration. For this, the non-STEC *E. coli* K12 was tested against O157 in the RSE cell adherence assay (Supplementary Figure S6) [20,23,24,26,27,33,35,50]. The qualitative and quantitative data generated, as shown in Supplementary Figure S6, demonstrated a diffuse, moderate adherence for *E. coli* K12, whereas O157 exhibited an aggregative, moderate adherence. There was a statistically significant difference in quantitative adherence (*p* < 0.014), further supporting the use of *E. coli* K12 in the comparative RAJ-IVOC adherence assays. Two pilot trials to verify the adherence of *E. coli* K12 (inoculum at 10^8^ CFU) to the RAJ-IVOC were conducted, and the same diffuse adherence was observed on the tissue with no histopathology (Supplementary Figure S7).

**Figure 9:**
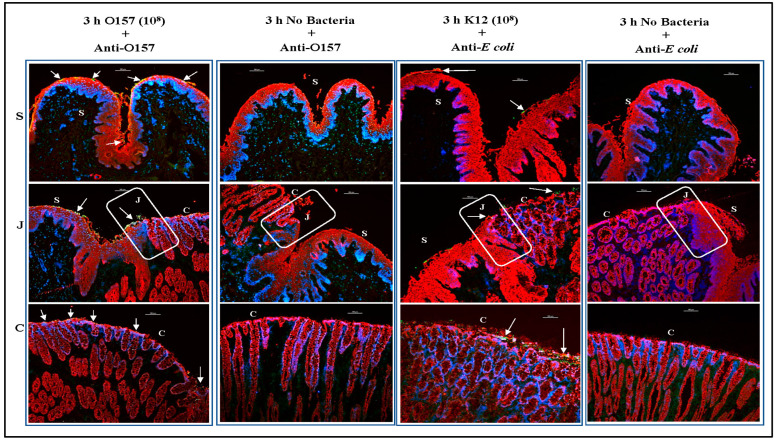
Immunofluorescent images of tissue sections from a RAJ-IVOC comparative adherence assay. The RAJ-IVOC were inoculated with either O157 (10^8^ CFU inoculum as shown in parenthesis) or *E. coli* K12 (10^8^ CFU inoculum as shown in parenthesis) or not inoculated (no bacteria) and incubated at 39 °C for 3 h. Tissue sections of the RAJ-IVOC were then stained with immunofluorescent antibodies targeting the RAJ cells’ cytokeratins and O157 or *E. coli*, and images were recorded at 100× magnification. The adherent bacteria (shown with arrows), RAJ cells’ cytokeratins, and the nuclei have green, orange–red, and blue fluorescence, respectively. The squamous (S), junction (J, boxed), and columnar (C) regions of the RAJ are indicated along with a 100 µm scale bar.

The O157 and *E. coli* K12 strains were subsequently evaluated in comparative assays with different inoculum concentrations. Assays with 10^8^ or 10^7^ CFU inoculum concentrations were conducted in triplicate, on separate days, with RAJ tissues from various animals (Supplementary Table S1). Likewise, the 10^6^ CFU inoculum concentration was evaluated in duplicate as this concentration had been evaluated in earlier trials (trials 1–3) and the comparative assay was only confirmatory (Supplementary Table S1). In the first set of comparative assays, a 10^8^ CFU inoculum was used, and as shown in Figure 9, the two bacteria adhered to the RAJ-IVOC after 3 h incubation in distinctive patterns. O157 exhibited consistent adherence patterns as observed in previous assays (Figure 6, Figure 7 and Figure 8), forming small aggregates along the squamous region and microcolonies along the columnar epithelia. On the other hand, *E. coli* K12 demonstrated diffuse adherence along the entire length of the RAJ-IVOC (Figure 9, Supplementary Figures S8–S11). No adherent bacteria were detected on the NB-RAJ-IVOC tissue sections in this set of comparative assays using the primary antibodies targeting O157 and *E. coli* (Figure 9, Supplementary Figures S8–S11).

**Figure 10:**
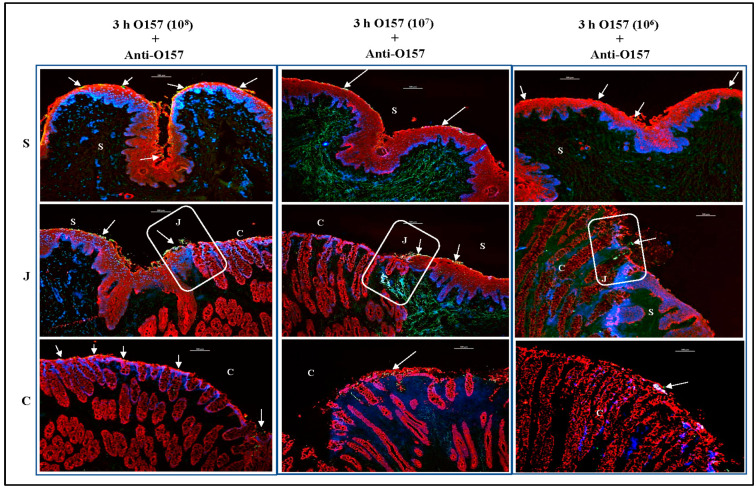
Immunofluorescent images of tissue sections from RAJ-IVOC comparative adherence assays. The tissue sections from RAJ-IVOC inoculated with either 10^8^, 10^7^, or 10^6^ CFU O157 (inoculum concentrations are shown in parentheses) and incubated at 39 °C for 3 h are shown. Tissue sections were stained with immunofluorescent antibodies targeting the RAJ cells’ cytokeratins and O157, and images were recorded at 100× magnification. The adherent bacteria (shown with arrows), RAJ cells’ cytokeratins, and the nuclei have green, orange–red, and blue fluorescence, respectively. The squamous (S), junction (J, boxed), and columnar (C) regions of the RAJ are indicated along with a 100 µm scale bar.

**Figure 11:**
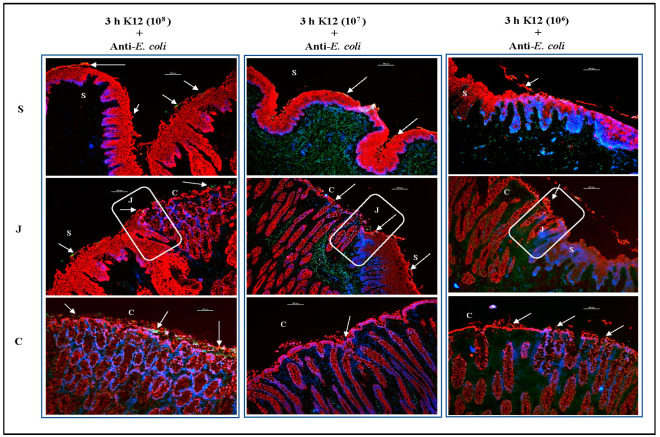
Immunofluorescent images of tissue sections from RAJ-IVOC comparative adherence assays. The tissue sections from RAJ-IVOC inoculated with either 10^8^, 10^7^, or 10^6^ CFU *E. coli* K12 (inoculum concentrations are shown in parentheses) and incubated at 39 °C for 3 h are shown. Tissue sections were stained with immunofluorescent antibodies targeting the RAJ cells’ cytokeratins and *E. coli*, and images were recorded at 100× magnification. The adherent bacteria (shown with arrows), RAJ cells’ cytokeratins, and the nuclei have green, orange–red, and blue fluorescence, respectively. The squamous (S), junction (J, boxed), and columnar (C) regions of the RAJ are indicated along with a 100 µm scale bar.

In the second and third sets of comparative assays, the 10^7^ and 10^6^ CFU inocula were evaluated, respectively. As shown in Figure 10 and Figure 11, O157 and *E. coli* K12 adhered in optimal numbers following inoculation with 10^7^ bacteria. Similar to the results of trials 1–4, a higher inoculum of 10^8^ CFU resulted in a greater number of adherent bacteria for both O157 and *E. coli* K12, while a lower inoculum of 10^6^ CFU resulted in fewer adherent bacteria (Figure 10 and Figure 11). Although overall adherence patterns were similar across inoculum levels, the number of bacteria on the tissue was closer to that observed in vivo when 10^7^ CFU inoculum was applied to the RAJ-IVOC (Figure 10 and Figure 11). Occasionally, O157 could be observed in the short crypts along the mucosal side of the junction in all assays. However, this association was not consistent, as the majority of the bacteria were distributed mostly outside the crypts. We cannot rule out the possibility that the 3 h incubation period may have limited any prolonged interactions with crypt cells. No adherent bacteria were detected on the NB-RAJ-IVOC tissue sections in all comparative assays.

**Figure 12:**
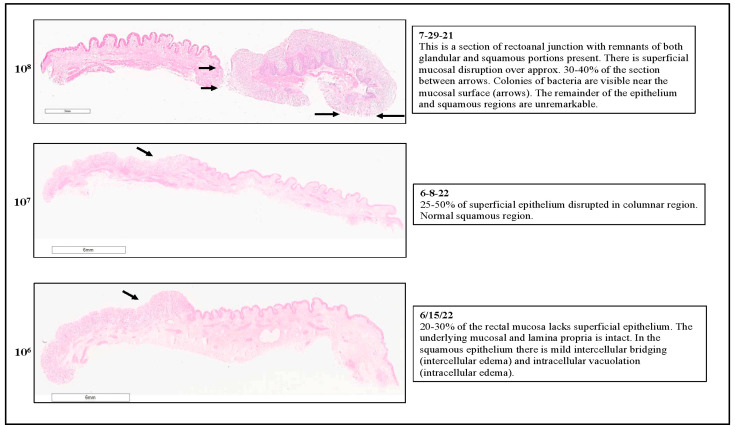
Representative histopathological reports of the comparative adherence assays using 10^8^, 10^7^, or 10^6^ CFU O157 as inoculum. The RAJ-IVOC were inoculated and incubated at 39 °C for 3 h. H&E-stained tissue section slides were scanned using the Aperio digital pathology system to obtain the eImages. Histopathological observations made are shown against each eImage. A 3 mm scale bar is shown.

**Figure 13:**
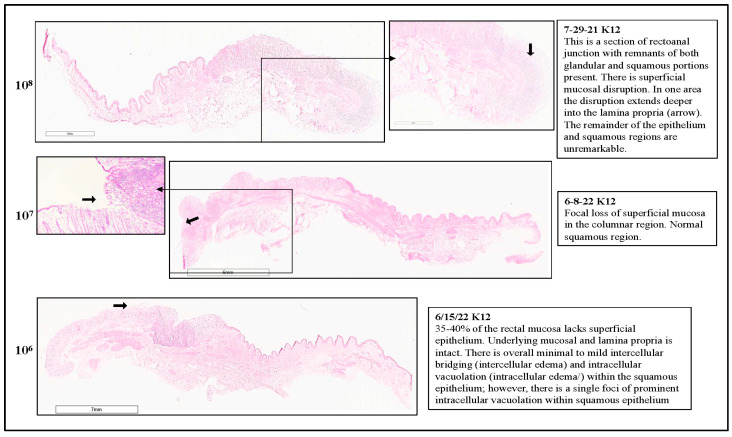
Representative histopathological reports of the comparative adherence assays using 10^8^, 10^7^, or 10^6^ CFU *E. coli* K12 as inoculum. The RAJ-IVOC were inoculated and incubated at 39 °C for 3 h. H&E-stained tissue section slides were scanned using the Aperio digital pathology system to obtain the eImages. Histopathological observations made are shown against each eImage. A 3 mm scale bar is shown.

Histopathological evaluations of the RAJ-IVOC tissues were conducted after each assay (Supplementary Histopathology Reports folder). A comparative sample of the reports is shown in Figure 12 and Figure 13 for the RAJ-IVOC tissues inoculated with 10^8^, 10^7^, and 10^6^ CFU O157 or *E. coli* K12. Corresponding details are also in the Supplementary Data section (Supplementary Figures S12–S17, Supplementary Histopathology Reports folder). Most pre-assay RAJ tissues demonstrated excellent tissue integrity in all regions at collection, except in one instance (Figure 12 and Figure 13, Supplementary Figures S12–S17, Supplementary Histopathology Reports folder). The glandular and squamous regions of the NB-RAJ-IVOC tissues remained unremarkable or had few disruptions consistent with those observed at collection or with tissue handling (Supplementary Figures S12–S17, Supplementary Histopathology Reports folder). Overall, considering the number of assay replicates per inoculum, the following histopathological observations were made:

#### 3.3.1. For RAJ-IVOC Inoculated with 10^8^ O157

Small to Significant Superficial Mucosal Disruption was Observed with Colonies of Bacteria Often Visible Near the Mucosal Surface. The remainder of the epithelium and squamous regions were mostly unremarkable (Figure 12 and Figure 13, Supplementary Figures S12 and S13, Supplementary Histopathology Reports folder). For RAJ-IVOC inoculated with 10^8^ *E. coli* K12, small regions of superficial mucosal disruption were observed. Deeper disruptions, when observed, were mostly located at the tissue edge and likely due to tissue handling during the experiment. The remainder of the epithelium and squamous regions were unremarkable (Figure 12 and Figure 13, Supplementary Figures S12 and S13, Supplementary Histopathology Reports folder).

#### 3.3.2. For RAJ-IVOC Inoculated with 10^7^ O157

Most tissues exhibited multiple small areas of superficial mucosal disruption in the columnar region, except for one (O157-6-8-22; Supplementary Figure S15, Supplementary Histopathology Reports folder), which had 25–50% mucosal disruption and normal squamous regions (Figure 12 and Figure 13, Supplementary Figures S14 and S15, Supplementary Histopathology Reports folder). For RAJ-IVOC inoculated with 10^7^ *E. coli* K12, a few small superficial mucosal disruptions were observed in the columnar region, with the squamous region remaining normal (Figure 12 and Figure 13, Supplementary Figures S14 and S15, Supplementary Histopathology Reports folder).

#### 3.3.3. For RAJ-IVOC Inoculated with 10^6^ O157 or *E. coli* K12

Disruption of the superficial mucosal epithelium was consistently observed, with the underlying mucosa and lamina propria remaining intact. The squamous epithelium had mild intercellular bridging (intercellular edema) and intracellular vacuolation (intracellular edema), which was also observed in the pre-assay tissue (Figure 12 and Figure 13, Supplementary Figures S16 and S17, Supplementary Histopathology Reports folder), likely due to post-mortem events and/or unknown underlying clinical conditions of the animal. Although this did not interfere with overall tissue viability or bacterial adherence, the greater number of non-STEC/non-*E. coli* K12 background flora was isolated from some of these tissues as discussed below.

The RAJ-IVOC tissue sections were also stained with labeled antibodies targeting *Salmonella* to demonstrate the specificity of the other antibodies used, and no signal was obtained from any of the sections, ruling out any possible cross-reactivity (Supplementary Figures S18 and S19). Considering all the observations and adherence results, the optimal condition for the RAJ-IVOC adherence assay was set at 39 °C for 3 h with a 10^7^ bacterial inoculum for a 2 cm × 4 cm piece of tissue.

### 3.4. Differences in O157 and E. coli K12 Recovered from the RAJ-IVOC Tissue Cultures Corresponded with the Adherence Patterns

**Figure 14:**
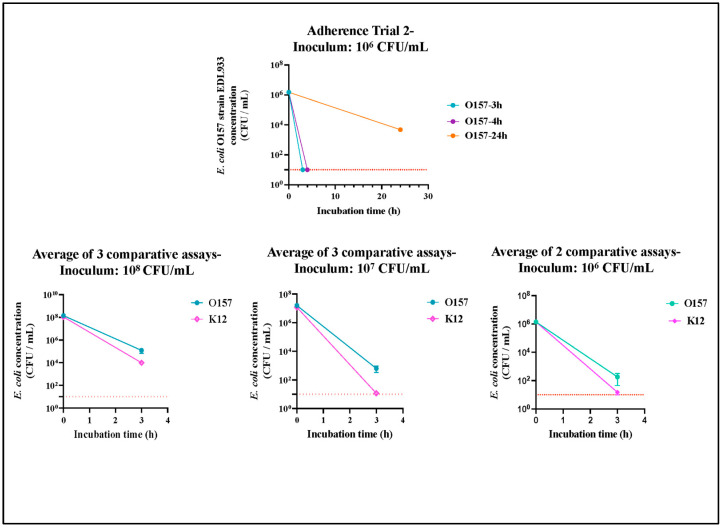
Graphs depicting the *E. coli* (O157 and K12) counts recovered from RAJ-IVOC tissues by culture. Results shown are from adherence trial 2 and the averages of comparative adherence assays conducted with different inoculum concentrations.

O157 produced typical sorbitol non-fermenting, colorless, MUG-non-utilizing, non-fluorescent colonies on all SMAC plates with MUG and readily agglutinated with the O157 latex agglutination reagent (Figure 1). *E. coli* K12 produced lactose-fermenting, pink, MUG-utilizing fluorescent colonies on all MAC plates with MUG (Figure 1). Following the culture of the RAJ-IVOC tissues, there was a variable reduction in recovered bacterial counts (CFU/mL; Supplementary Table S1) for assays using the 10^8^ or 10^6^ inocula. O157 demonstrated a 3- to 5-log reduction in counts, while *E. coli* K12 demonstrated a 3- to 6-log reduction in the two sets of comparative assays (Figure 14, Supplementary Table S1, Supplementary Figure S20A,B). On the other hand, with a 10^7^ inoculum, O157 counts were reduced by 4- to 5-logs and *E. coli* K12 counts were consistently reduced by 6-logs (Figure 14, Supplementary Table S1, Supplementary Figure S20A,B). The 10^7^ inoculums yielded more accurate bacterial counts that corresponded to the adherence patterns observed via microscopy on the RAJ-IVOC. It demonstrated optimal adherence to the tissue, even when considering the extrapolated bacterial counts per gram of tissue (Supplementary Table S1). Overall, O157 exhibited a higher recovery count by culture compared to *E. coli* K12, on the RAJ-IVOC tissue, likely due to its tendency to form microcolonies or small aggregates. However, these differences in counts between O157 and *E. coli* K12 were not statistically significant (*p* = 0.9).

### 3.5. PATS Verified Bacteria Recovered from the RAJ-IVOC Tissue Cultures

DNA fingerprinting using PATS enabled the verification of bacteria recovered from the RAJ-IVOC tissue cultures against the inoculated O157 and *E. coli* K12 strains. In addition, any non-pinpoint colonies isolated from the RAJ-IVOC inoculation media or tissues after incubation (Supplementary Table S1) were subjected to typing. The distinctive PATS profiles for O157 and *E. coli* K12 were determined before utilizing these strains in the comparative assays (Table 1, Supplementary Figure S21). Subsequent evaluation of bacteria recovered from O157 or *E. coli* K12 inoculated RAJ-IVOC media and tissue cultures matched the respective profiles (Table 1). Interestingly, the additional bacteria isolated from some RAJ-IVOC media or tissues, especially those used in the final comparative assays (Supplementary Table S1), exhibited five different PATS profiles. Some of these bacteria were found to carry STEC virulence genes, such as stx2 and/or hlyA (Table 1). None of these colonies were agglutinated with the O157 latex agglutination reagent.

## 4. Discussion

Establishing the RAJ as the site of STEC persistence, especially for the serogroup O157 [12,13,52], resulted in the development of more targeted and rapid animal sampling [53,54,55,56] and animal infecting methods [57], besides several RAJ-based host-bacterial studies. For instance, Wang et al. found that RAJ microbiota composition and gene expression differ between supershedders and non-supershedding cattle [31]. *E. coli* expressing the virulence factors intimin and the translocated intimin receptor were found to persist better at the RAJ [28]. Additionally, the upregulation of host genes, including polymeric immunoglobulin receptor, beta-catenin, and keratinocyte growth factor precursor, appeared to contribute to O157 adherence and persistence through cytoskeleton changes, an increase in cell surface area, and enhanced nutrient availability [29]. Upon O157 colonization, the cattle immune response was found to downregulate cortactin and upregulate wheat germ agglutinin (WGA)-lectin post-challenge to clear bacteria at the RAJ [29,32]. However, certain subpopulations of O157 could become internalized by rectal epithelial cells, aiding their survival and persistence at the RAJ [30].

Coinciding with the above RAJ studies, we have previously developed an in vitro adherence assay utilizing bovine RAJ squamous epithelial (RSE) cells. This assay was designed to compare the adherence patterns of O157 and non-O157 STEC strains, facilitating the identification of pathogen- and host-specific interactions at the RAJ. The primary goal of this assay was to identify molecular mechanisms underlying adherence [20]. Using this adherence assay, the locus of enterocyte effacement (LEE)-encoding proteins, which are known to be critical for adherence to FAE cells at the RAJ, did not play a role in adherence to bovine RSE cells. This finding highlights additional distinctions between O157 and non-O157 STEC adherence to the RAJ cells [23,24]. On the other hand, outer membrane protein A and curli modulated or tempered O157 adherence to RSE cells [25,26], and *iha* and *cah* were essential for the aggregative phenotype of O157 strain EDL933 but not for super-shed O157 strain SS17 [33,58]. The unique aggregative, strong adherence phenotype of super-shed O157 on bovine RSE cells could be contributing to their persistence in cattle [27,59]. Experimentally challenging cattle with O157 altered the bovine intestinal microbial community and promoted site-specific colonization at the RAJ [22]. In vaccinated cattle, however, locally produced interferon gamma impacted bacterial attachment to RAJ epithelial cells and possibly reduced fecal O157 shedding [60].

Even though all these studies, including ours, have provided some insights into O157 persistence at the RAJ, additional studies are needed to determine factors associated with differential adherence to the RAJ cell types and between O157/non-O157 STEC serogroups [23,24]. Although the RSE cell adherence assay is the closest to a host- and site-relevant assay available, a more comprehensive analysis of the entire RAJ-STEC interaction requires in vivo studies. Such studies often result in animal euthanasia, exposing the need for an in vitro assay that minimizes animal usage yet reflects the results observed in vivo. Hence, we decided to develop a RAJ in vitro organ culture (RAJ-IVOC) model system with both RSE and FAE cells, which would permit bacterial adherence studies with the same results as those observed in vivo in animals and in the in vitro RSE-cell assay.

Ex vivo organ cultures are often utilized for work with human and clinical tissue samples [61]. Several studies on human immunodeficiency virus (HIV) have utilized such organ cultures to study the early transmission of the virus in a combined human cervical and tonsil tissue system [62], to study infection in human cervicovaginal tissue [63], as well as to design models of oral transmission in human palatine tonsils [64]. Ex vivo organ cultures have also been used to study the interaction between *Mycobacteria* and mucins MUC5AC and MUC5B using human mucosal respiratory tissue [65]. Adherence of *Mycobacteria* and post-infection tissue mucus production were examined, which are important factors in understanding the pathogenicity of *Mycobacteria* in diseases such as cystic fibrosis and chronic obstructive pulmonary disease [65]. The pharyngeal tonsil tissue from Holstein steers was utilized to gain a better understanding of tonsillar function in pathogenesis and as a potential site for mucosal immune responses upon vaccination [66]. The epithelial surface of the tissue was examined via transmission electron microscopy to characterize the morphology, distribution, and cell type within the reticular epithelium, which is a key site for immune cells and antigen uptake and processing in cattle [66]. Additionally, the tonsil tissue, covered with variously sized latex beads, was used to examine microparticle uptake in M-cells, which help transport external antigens to immune cells in the underlying lymphoid tissue to initiate a host immune response [66]. Lastly, the relationship between enterohemorrhagic *E. coli* (EHEC) and intimin-related tissue tropism has been studied using human intestinal tissue cultures collected from children undergoing routine intestinal disorder examinations [67]. After collection, the tissue was infected with a transformed enteropathogenic *E. coli* strain O127:H7 expressing either intimin-α or intimin-γ and examined via scanning electron microscopy. Strains expressing intimin-α were found to adhere to the small intestine and follicle-associated epithelium of Peyer’s patch with minimal adhesion in the colon tissue, whereas strains expressing intimin-γ were limited to adhesion to Peyer’s patch and induced attaching and effacing lesions similar to those of EHEC O157:H7 [68].

Based on these reports, we addressed the need for a RAJ-IVOC model system that would allow for in vitro screening of O157 and the selection of relevant strains for limited in vivo studies. The RAJ-IVOC model system was standardized by (i) developing the basic assembly and incubation methods while monitoring tissue integrity and viability, and (ii) deriving optimal conditions for obtaining the required adherence results. The optimal conditions for the RAJ-IVOC model system include careful collection, transport, and rinsing of the RAJ tissue. Additionally, proper assembly of scaffold materials is essential. It is important to use DMEM-LG only below the agarose seal to maintain tissue viability, while DMEM-NG is used above the agarose seal to prevent inordinate growth of inoculated bacteria (Figure 1; see ‘Section 2. Material and Methods’). No variations between polystyrene plates/dishes and glass dishes were observed, which provides convenient options for the user. The ex vivo cultures described above [61,62,63,64,65,66,67,68] entailed infection for 1–2 h followed by extended incubation for a maximum of 20 days when inoculated with viruses, 7 days for *Mycobacterium tuberculosis,* and 8 h for enteropathogenic/enterohemorrhagic *E. coli*. However, in those instances, cells sloughed routinely over time, as observed with the RAJ-IVOCs incubated for 24 h in our study. Histopathological, cell marker, and viability evaluations indicated that RAJ-IVOC integrity and viability were best-maintained post-incubation for 3 h, with a possible extension to 4 h, at 39 °C. Further standardization with bacteria demonstrating contrasting adherence patterns confirmed the optimal test conditions, besides setting the optimal inoculation dose at a total of 10^7^ CFU, irrespective of the suspension volume. Following these standardized steps allowed for minimal contamination with background flora and reproduction of the results as those observed in vivo in animals and in the in vitro RSE-cell assay for the O157 and *E. coli* K12 bacteria tested.

Overall, the RAJ-IVOC model system allows for studying O157-RAJ interactions in situ on the organ, even under in vitro conditions. In addition, the model can be readily adapted to study other STEC/non-STEC bacterial interactions with the RAJ. More importantly, with this system, multiple bacterial strains and site-specific therapeutic modalities can be pre-screened prior to any in vivo studies, thereby reducing animal usage.

## Figures and Tables

**Table 1:** PATS profiles of isolates recovered from IVOC tissue cultures.

PATS Type	Polymorphic *Xba*I sites								Polymorphic *Avr*II sites							Virulence Genes				Strain
	IK8	IK25	IK114	IK118	IK123	IK127	IKB3	IKB5	IKNR3	IKNR7	IKNR10	IKNR12	IKNR16	IKNR27	IKNR33	*stx1*	*stx2*	*eaeA*	*hlyA*	
I	0	0	0	0	0	1	0	0	0	0	0	0	1	1	0	0	0	0	0	*E. coli* K12
II	0	0	1	1	1	1	1	1	2	2	2	2	2	2	2	1	1	1	1	O157 strain EDL933
III	0	0	0	0	0	0	0	0	0	1	1	0	1	1	0	0	1	0	0	A ^1^
IV	0	0	0	0	0	0	0	0	0	1	1	0	1	1	0	0	1	0	1	B ^2^
V	0	0	0	0	0	0	0	0	0	0	0	0	0	0	0	0	0	0	0	C ^3^
VI	0	0	0	0	1	1	0	0	0	0	0	0	1	1	2	0	0	0	0	D ^4^
VII	0	0	0	0	1	1	0	0	1	0	0	0	1	1	2	0	0	0	0	E ^5^

^1^ A: Isolates recovered on 15 June 2022 (Comparative assay with 10^6^ CFU/ml inoculum) from post assay media from (1) no bacteria inoculated IVOC on SMAC-MUG (S+M+O-) and (2) K12 inoculated IVOC on Mac-MUG (L+M+O-), and tissue culture-non-enrichment from (3) no bacteria inoculated IVOC on SMAC-MUG (S+M+O-) and 4) K12 inoculated IVOC on Mac-MUG (L+M+O-). ^2^ B: Isolates recovered on 30 March 2022 (Comparative assay with 10^7^ CFU/ml inoculum) from no bacteria inoculated IVOC, post assay media on SMAC-MUG (S+M+O-), and on 15 June 2022 (Comparative assay with 10^6^ CFU/ml inoculum) from no bacteria inoculated IVOC, tissue culture-enrichment on (1) Mac-MUG (L-M+O-), (2) SMAC-CTMV (S+M-O-), and (3) SMAC-CTMV (S-M-O-). ^3^ C: Isolates recovered on 29 June 2022 (Comparative assay with 10^6^ CFU/ml inoculum) from no bacteria inoculated IVOC, tissue culture-enrichment on (1) Mac-MUG (L-M-O-) and (2) SMAC-CTMV (S-M-O-). ^4^ D: Isolates recovered on 29 June 2022 (Comparative assay with 10^6^ CFU/ml inoculum) from no bacteria inoculated IVOC, tissue culture-enrichment on (1) Mac-MUG (L+M+O-) and (2) SMAC-CTMV (S+M+O-). ^5^ E: Isolate recovered on 29 June 2022 (Comparative assay with 10^6^ CFU/ml inoculum) from K12 inoculated IVOC, tissue culture-enrichment on Mac-MUG (L+M+O-). Acronyms Used in the Footnotes: MUG: 4-methylumbelliferyl-β-D-glucuronide; Mac-MUG: MacConkey agar with MUG; SMAC-MUG: Sorbitol MacConkey agar with MUG; SMAC-CTMV: Sorbitol MacConkey agar supplemented with cefixime, potassium tellurite, MUG, and vancomycin; S: Sorbitol; L: Lactose; M: MUG; O: O157 latex agglutination test.

## Data Availability

All relevant data has been included in the manuscript, either in the main or Supplementary Materials.

## Supplementary Materials

The following supporting information can be downloaded at: https://www.mdpi.com/article/10.3390/microorganisms11051289/s1, Figure S1. eImages of RAJ-IVOC tissue sections from Trial 1; Figure S2. eImages of RAJ-IVOC tissue sections from Trial 2; Figure S3. eImages of RAJ-IVOC tissue sections from Trial 3; Figure S4. eImages of RAJ-IVOC tissue sections from Trial 4; Figure S5. Images of a fixed-RAJ-IVOC tissue stained with DAPI (Panel 1) and REdDot2 (Panel 2); Figure S6. Qualitative (Panel A) and quantitative (Panel B) data from the RSE cell adherence assay; Figure S7. H&E-stained eImages and immunofluorescent (IF) images of tissue sections from two RAJ-IVOC pilot trials, A and B, with *E. coli* K12; Figure S8. Stitched immunofluorescent images of a tissue section from a representative ‘no bacteria-RAJ-IVOC’ control used in the comparative adherence assay; Figure S9. Stitched immunofluorescent images of a tissue section from a representative ‘no bacteria-RAJ-IVOC’ control used in the comparative adherence assay; Figure S10. Stitched immunofluorescent images of a tissue section from a representative RAJ-IVOC inoculated with 10^8^ CFU O157 in the comparative adherence assay; Figure S11. Stitched immunofluorescent images of a tissue section from a representative RAJ-IVOC inoculated with 10^8^ CFU *E. coli* K12 in the comparative adherence assay; Figure S12. eImages of RAJ-IVOC tissue sections from a comparative adherence assay using the 10^8^ CFU inoculum; Figure S13. Representative histopathological report of a comparative adherence assay using the 10^8^ CFU inoculum; Figure S14. eImages of RAJ-IVOC tissue sections from a comparative adherence assay using the 10^7^ CFU inoculum; Figure S15. Representative histopathological report of a comparative adherence assay using the 10^7^ CFU inoculum; Figure S16. eImages of RAJ-IVOC tissue sections from a comparative adherence assay using the 10^6^ CFU inoculum; Figure S17. Representative histopathological report of a comparative adherence assay using the 10^6^ CFU inoculum; Figure S18. Immunofluorescent images of tissue sections from a representative RAJ-IVOC inoculated with 10^8^ CFU O157 or *E. coli* K12 in a comparative adherence assay; Figure S19. Immunofluorescent images of tissue sections from a representative RAJ-IVOC inoculated with 10^7^ or 10^6^ CFU O157 or *E. coli* K12 in a comparative adherence assay; Figure S20A. Graphs representing O157 counts recovered from RAJ-IVOC tissues by culture in adherence trials 1 to 4; Figure S20B. Graphs representing *E. coli* (O157 and K12) counts recovered from RAJ-IVOC tissues by culture in comparative adherence assays conducted with different inoculum concentrations; Figure S21. Electrophoretic patterns of amplicons comprising representative PATS profiles on 3% agarose gels; Supplementary Table S1. RAJ-IVOC assay data; Supplementary Data: Histopathology Reports, S1–S6.
