# Supplementary material for: Bovine Rectoanal Junction In Vitro Organ Culture Model System to Study Shiga Toxin-Producing Escherichia coli Adherence
Source: Microorganisms. 2023 May 15;11(5):1289. doi: 10.3390/microorganisms11051289 (PMC10220872; doi:10.3390/microorganisms11051289)
Supplement: Supplementary file 1 [file microorganisms-11-01289-s001.zip › Supp.Data-Histopathology Reports/Supp.Data-HistopathologyReport-S1-Trials 1-3-11-4-19_11-13-19_1-23-20.pdf]

Dr. Indira Kudva

Histopathology Results
IVOC samples, 11-4-19, 11-13-19, 1-23-20
by
Dr. Mitchell Palmer
4-6-2020

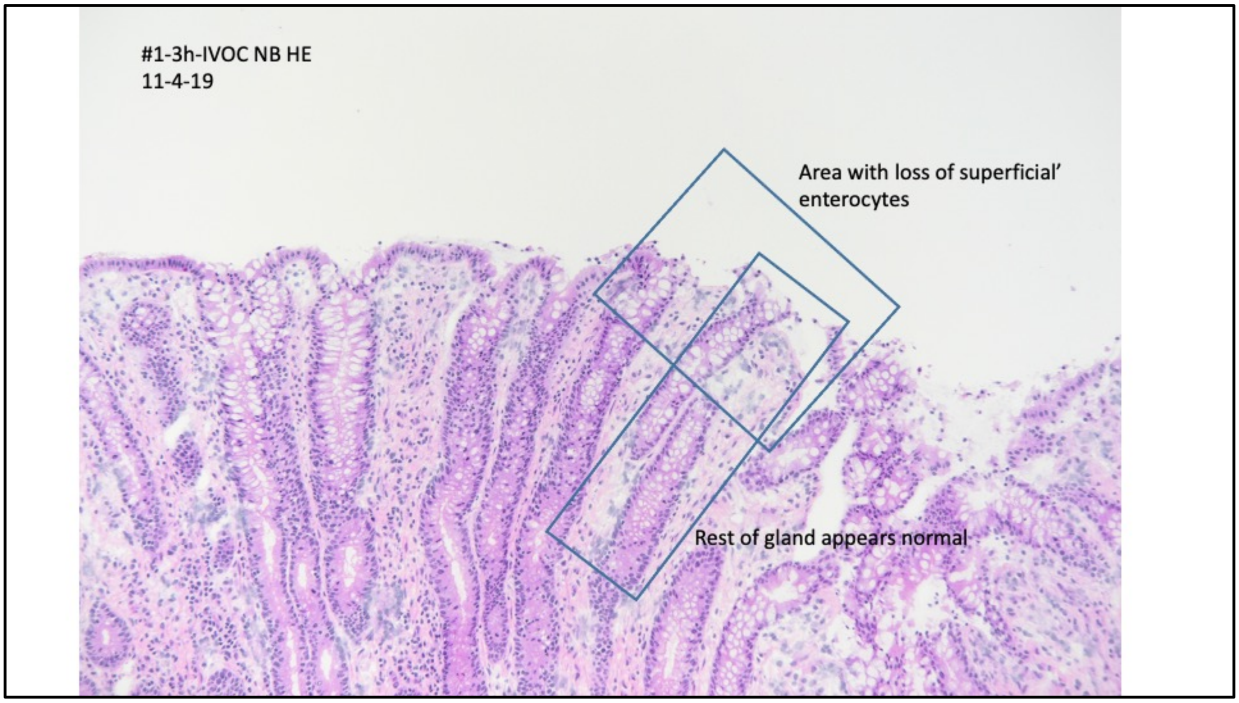

#1-3h-IVOC NB HE
11-4-19
This is a section of rectoanal junction with both glandular and squamous portions present. The glandular mucosa is mostly intact. In approx. 5-10% of the section the most apical enterocytes (those closest to the lumen) are absent. All deeper portions of the individual glands are intact and the lamina propria normal in appearance. The squamous region in non-remarkable.

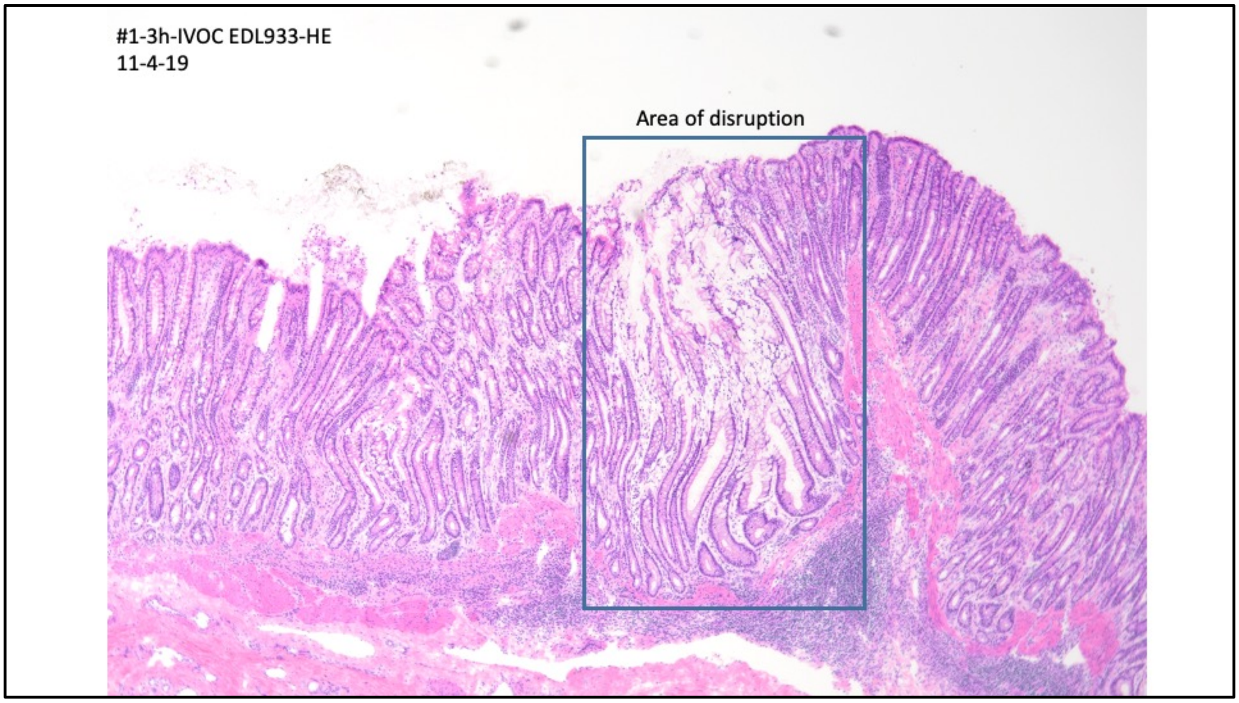

#1-3h-IVOC EDL933-HE
11-4-19
This is a section of rectoanal junction with both glandular and squamous portions present. The glandular mucosa is mostly intact. In approx. 5% of the section the mucosa is disrupted, lacking superficial enterocytes. Disruption extends into region of lamina propria. No bacteria were noted. The squamous portion is unremarkable.

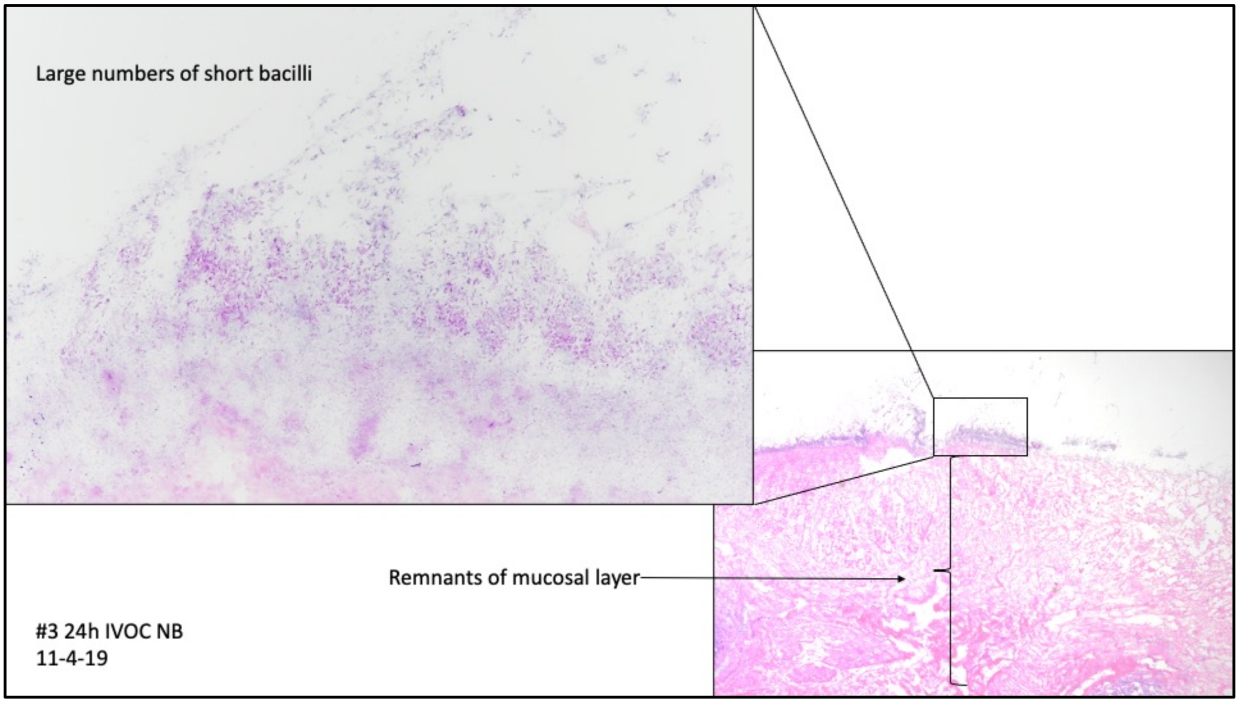

#3 24h IVOC NB
11-4-19
This is a section of rectoanal junction with remnants of both glandular and squamous portions present. There is entire loss of mucosa and in it's place is amorphous eosinophilic material. On the surface are large numbers of short bacilli. The squamous region is also characterized by cellular degeneration and necrosis.

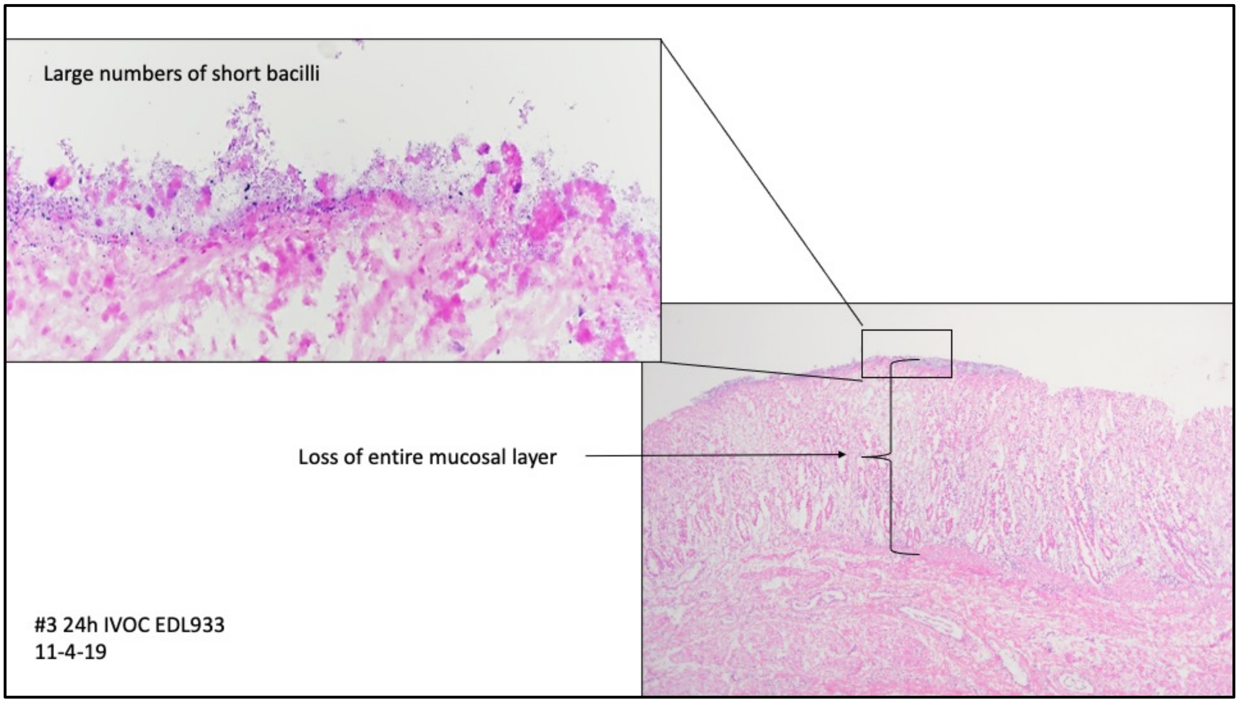

#3 24h IVOC EDL933
11-4-19
This is a section of rectoanal junction with remnants of both glandular and squamous portions present. There is entire loss of mucosa and in it’s place is amorphous eosinophilic material. On the surface are large numbers of short bacilli. The squamous region is also characterized by cellular degeneration and necrosis.

#3 24h IVOC EDL933
11-4-19

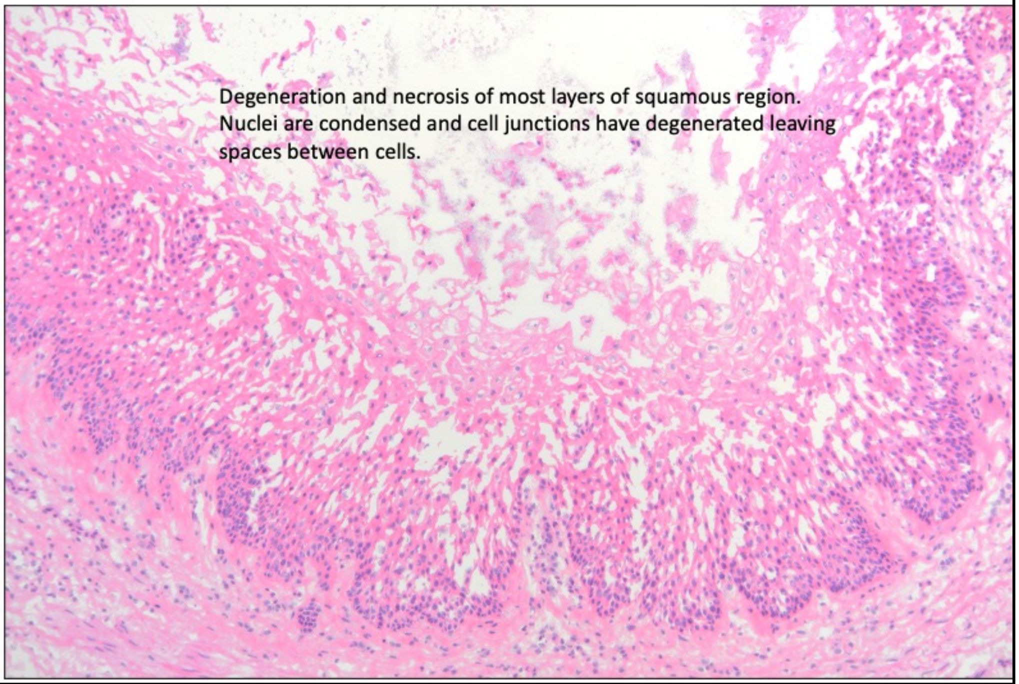

#3 24h IVOC EDL933
11-4-19
The squamous region is also characterized by cellular degeneration and necrosis.

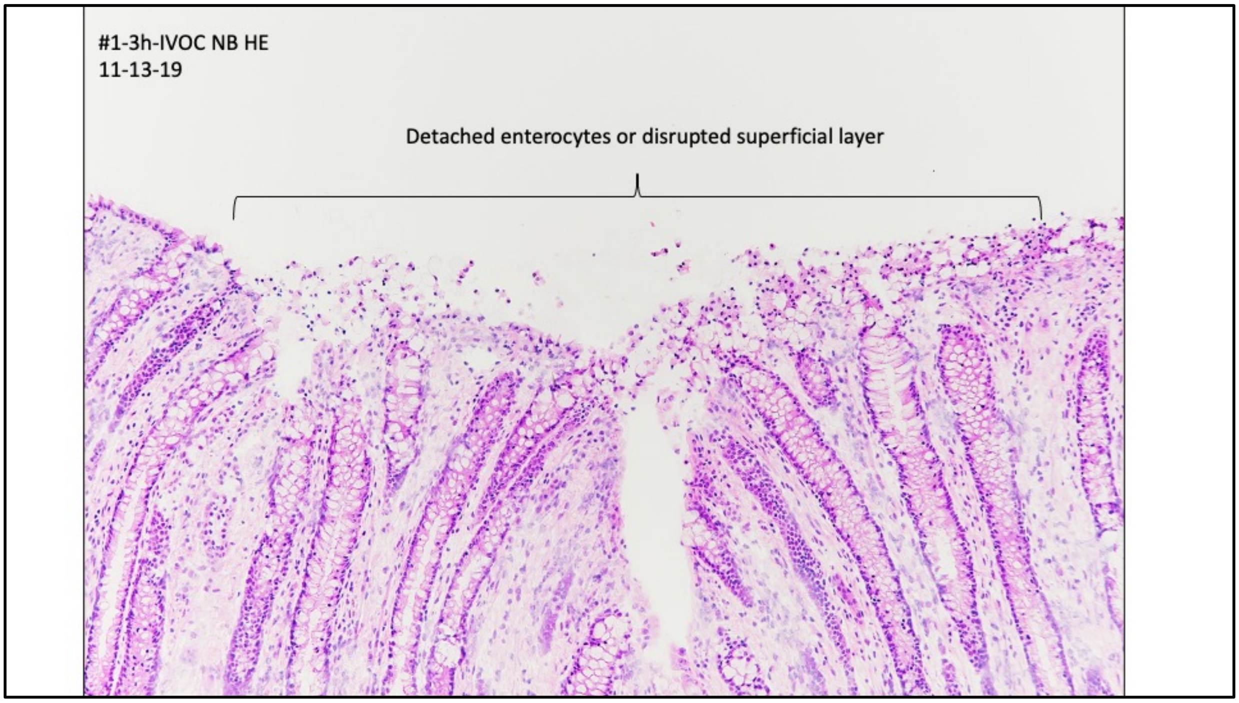

#1-3h-IVOC NB HE
11-13-19
This is a section of rectoanal junction with both glandular and squamous portions present. The glandular mucosa is mostly intact. In approx. 10-15% of the section the most apical enterocytes (those closest to the lumen) are absent. All deeper portions of the individual glands are intact. The squamous region in non-remarkable.

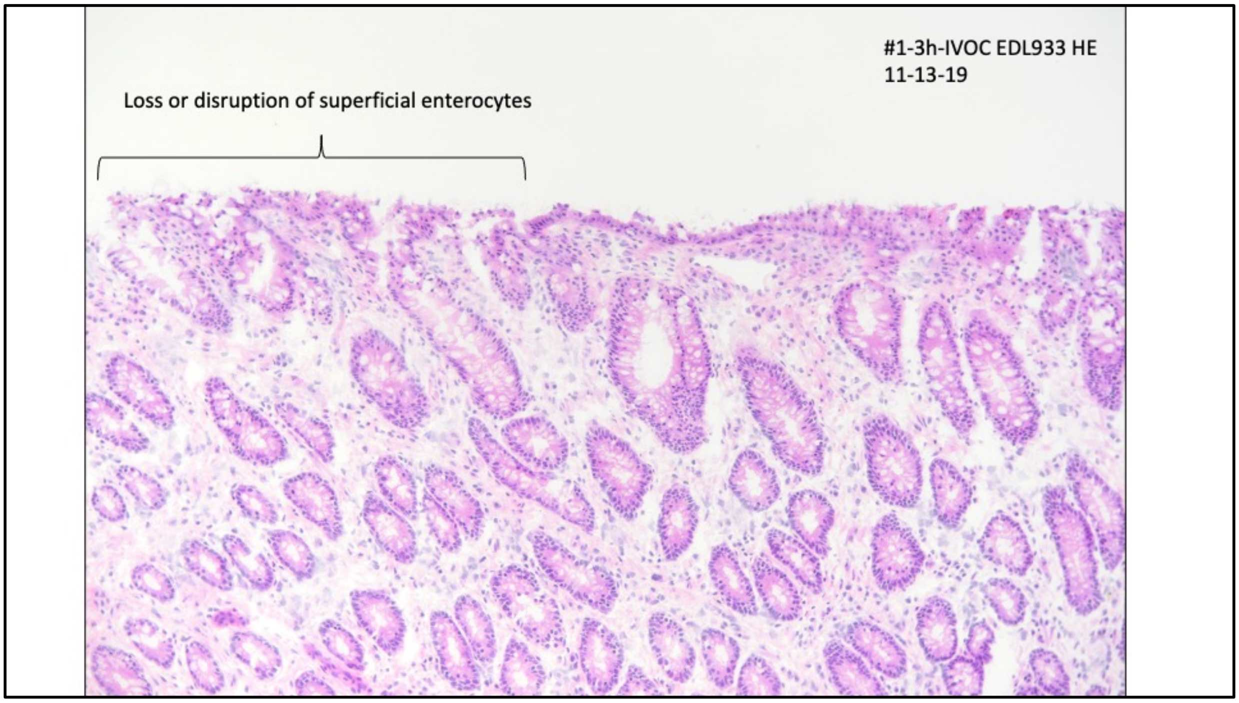

#1-3h-IVOC EDL933 HE
11-13-19
This is a section of rectoanal junction with both glandular and squamous portions present. The glandular mucosa is mostly intact. In approx. 20-25% of the section the most apical enterocytes (those closest to the lumen) are absent. All deeper portions of the individual glands are intact. The squamous region in non-remarkable.

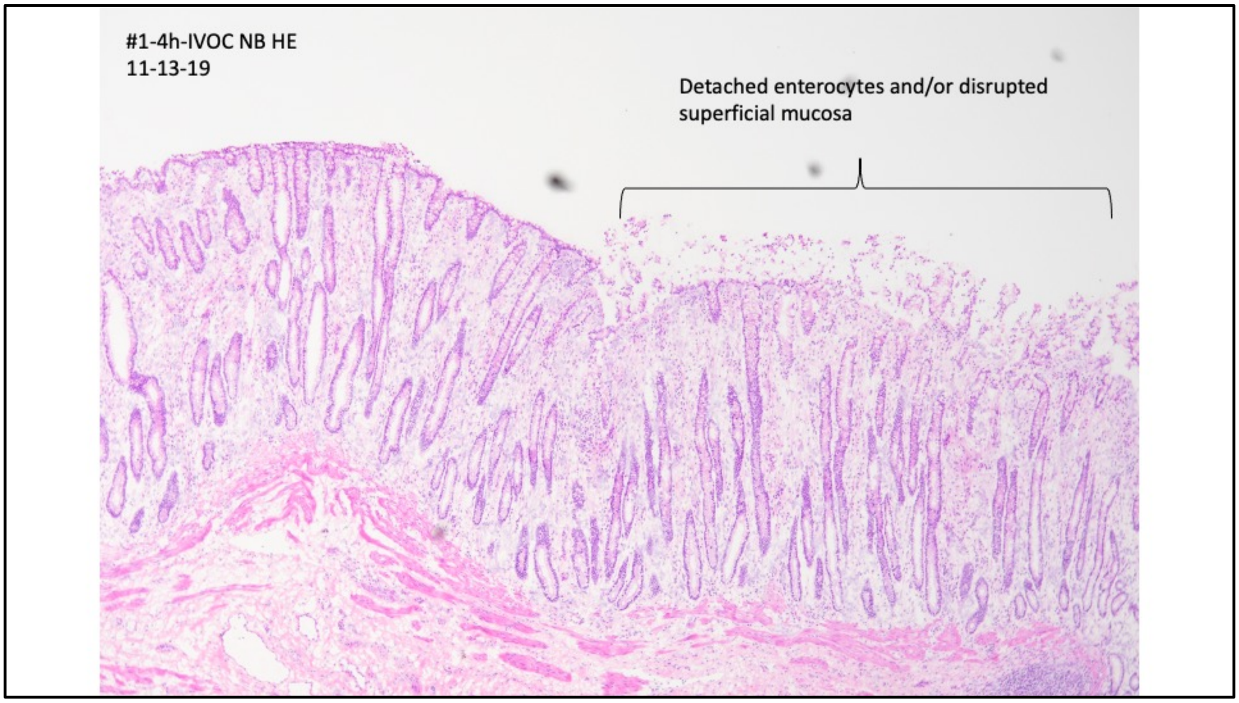

#1-4h-IVOC NB HE
11-13-19
This is a section of rectoanal junction with both glandular and squamous portions present. The glandular mucosa is mostly intact. In approx. 25-30% of the section the most apical enterocytes (those closest to the lumen) are absent or the superficial mucosa is disrupted. In some regions the lamina propria between glands is expanded and rarefied (less dense). The squamous region in non-remarkable.

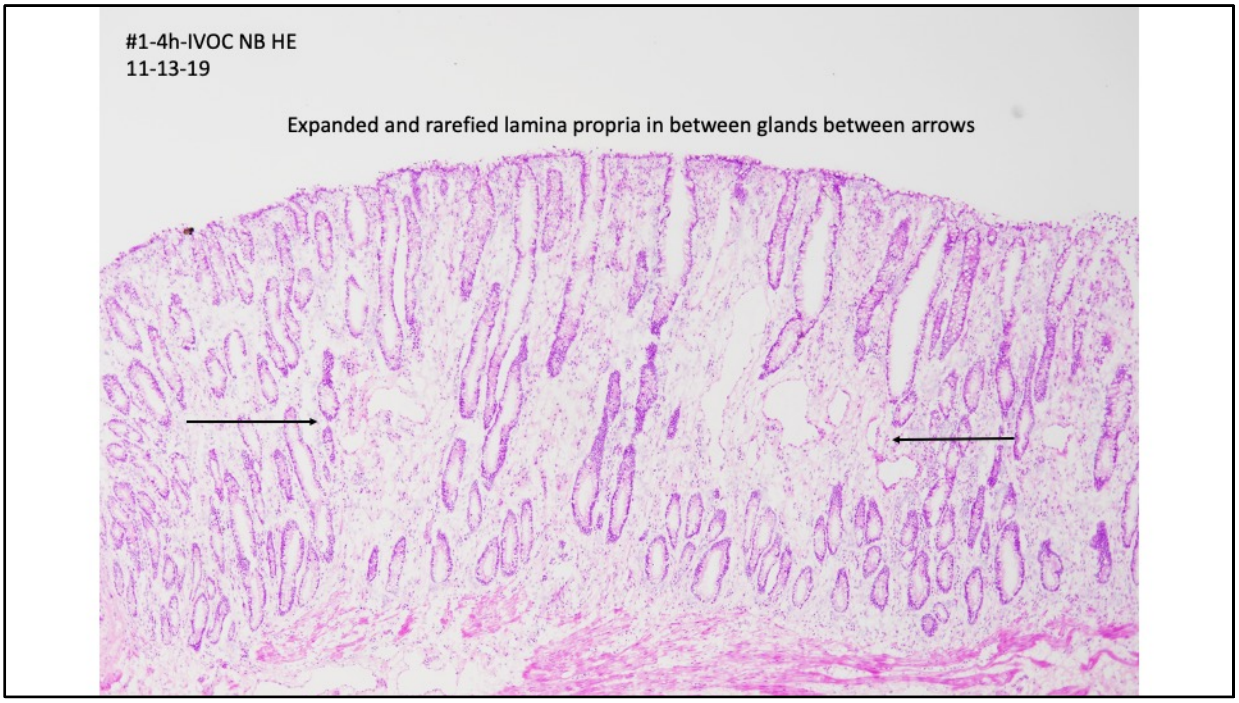

#1-4h-IVOC NB HE
11-13-19
In some regions the lamina propria between glands is expanded and rarefied (less dense).

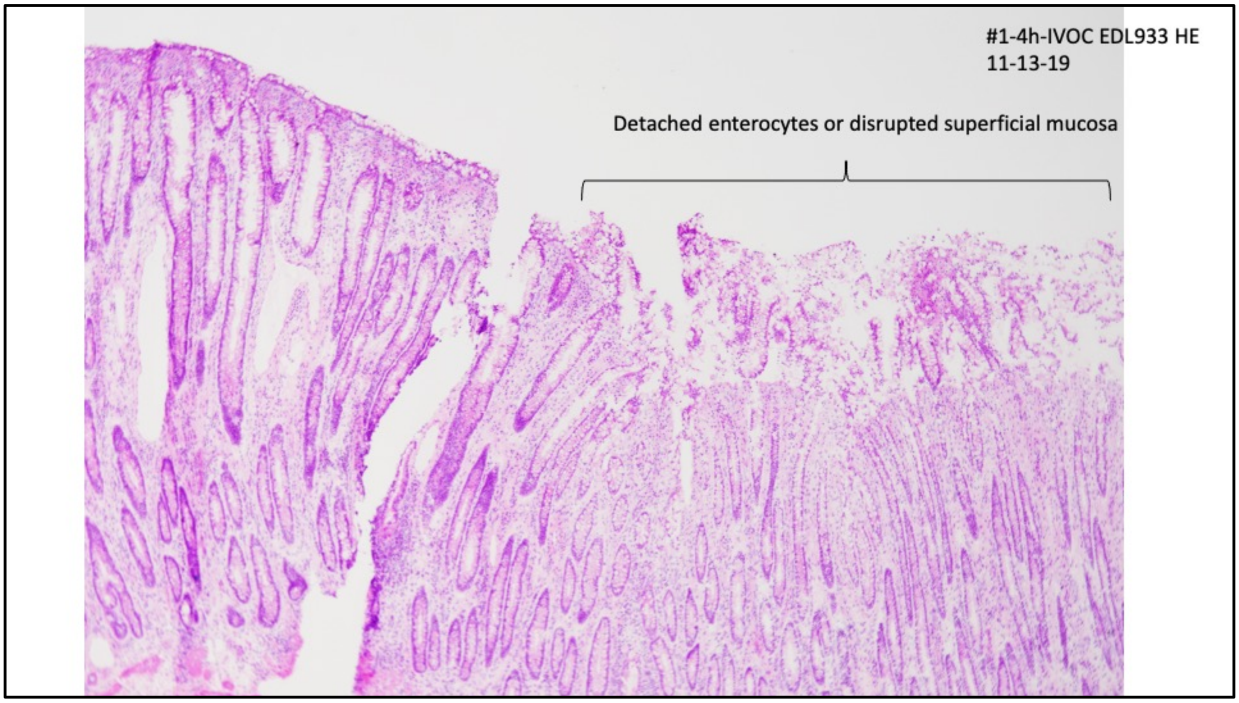

#1-4h-IVOC EDL933 HE
11-13-19
This is a section of rectoanal junction with both glandular and squamous portions present. The glandular mucosa is mostly intact. In approx. 25-30% of the section the most apical enterocytes (those closest to the lumen) are absent or the superficial mucosa is disrupted. Deeper areas of the gland appear normal. The squamous region in non-remarkable.

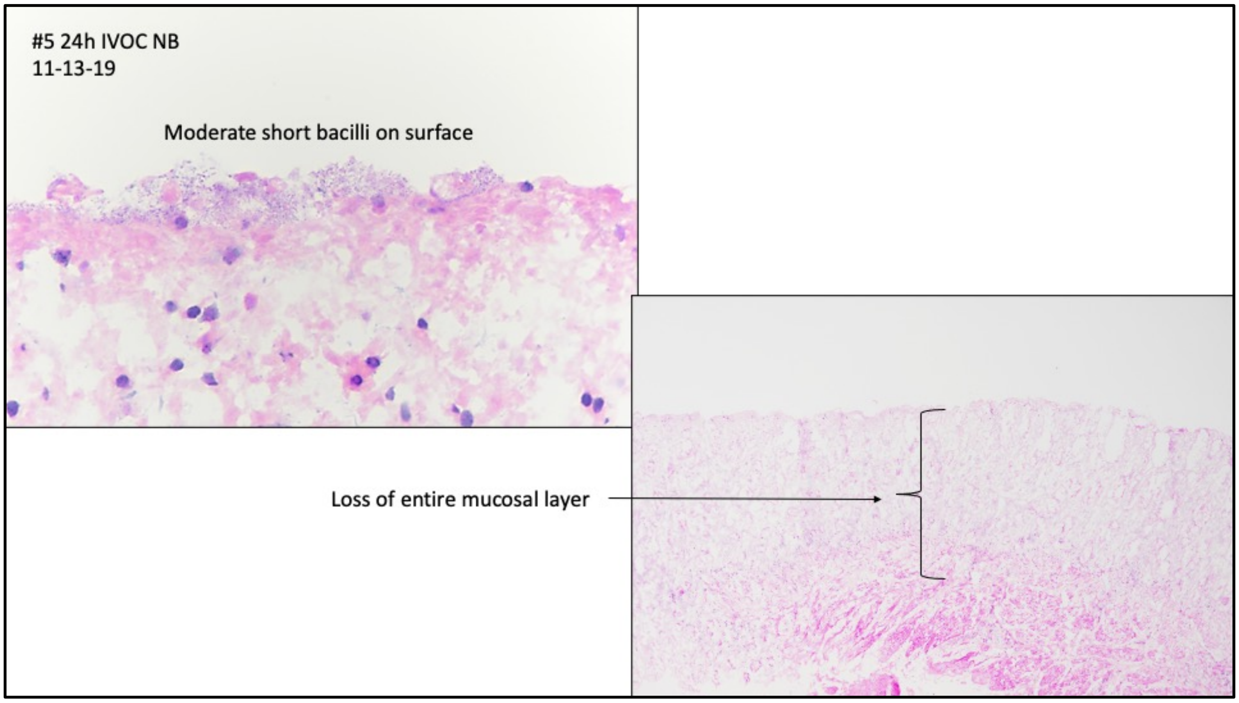

#5 24h IVOC NB
11-13-19
This is a section of rectoanal junction with remnants of both glandular and squamous portions present. There is entire loss of mucosa and in it’s place is amorphous eosinophilic material. On the surface are large numbers of short bacilli. The squamous region is also characterized by cellular degeneration and necrosis.

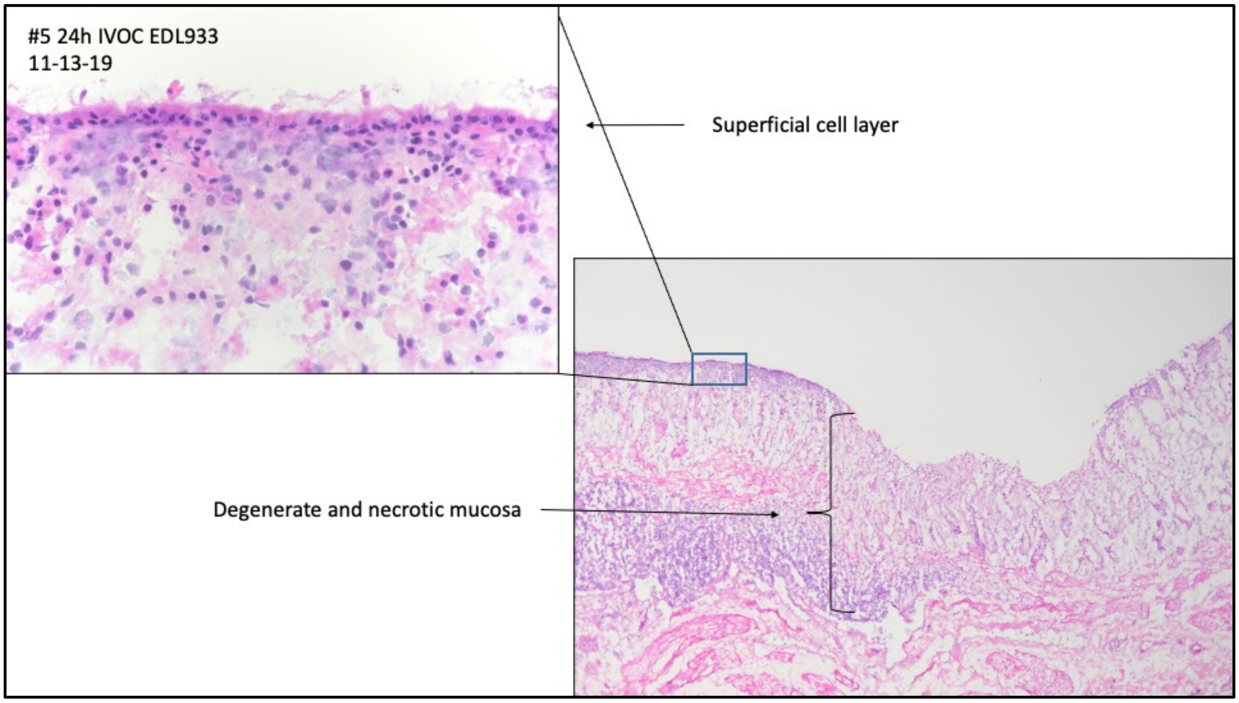

#5 24h IVOC EDL933
11-13-19
This is a section of rectoanal junction with remnants of both glandular and squamous portions present. There is near entire loss of mucosa and in it's place is amorphous eosinophilic material. In some regions a superficial cell layer remains on the luminal surface; however, deeper areas of the gland and lamina propria are degenerate and necrotic. On the surface are low numbers of short bacilli. The squamous region is also characterized by cellular degeneration and necrosis.

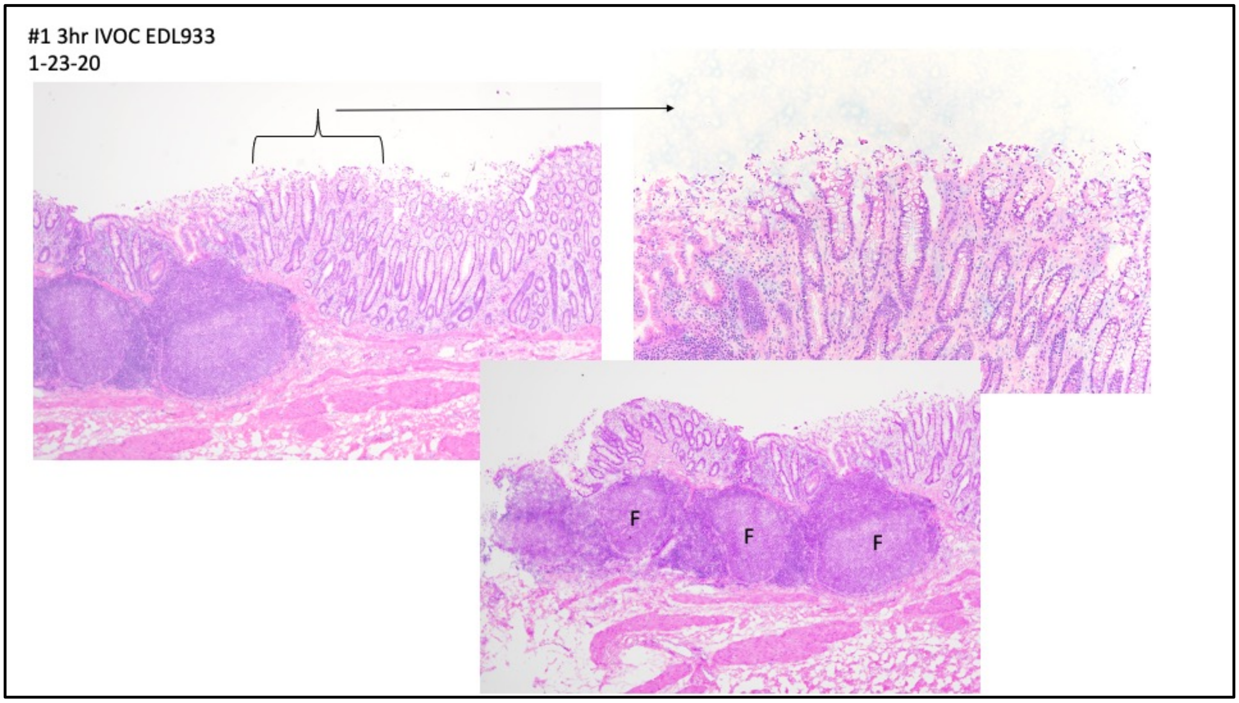

#1 3hr IVOC EDL933
1-23-20
This is a section of rectoanal junction with both glandular and squamous portions present. There is a focal area of mucosal disruption in the glandular region. The remaining glandular region and the squamous region are normal in appearance. A portion of the glandular region has underlying lymphoid follicles (F) and therefore represents lymphoepithelium.

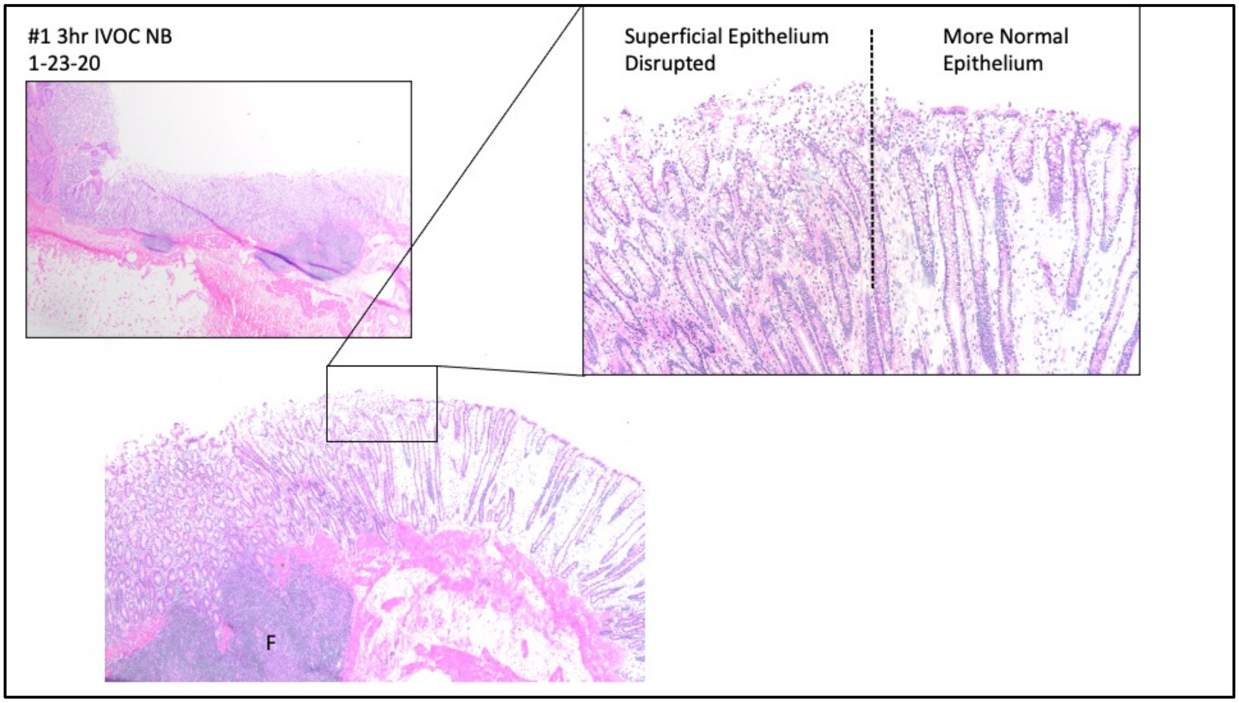

#1 3hr IVOC NB
1-23-20
This is a section of rectoanal junction with both glandular and squamous portions present. Approximately 50% of the glandular portion is characterized by superficial mucosal disruption. The remaining glandular region and the squamous region are normal in appearance. A portion of the glandular region has underlying lymphoid follicles (F) and therefore represents lymphoepithelium.

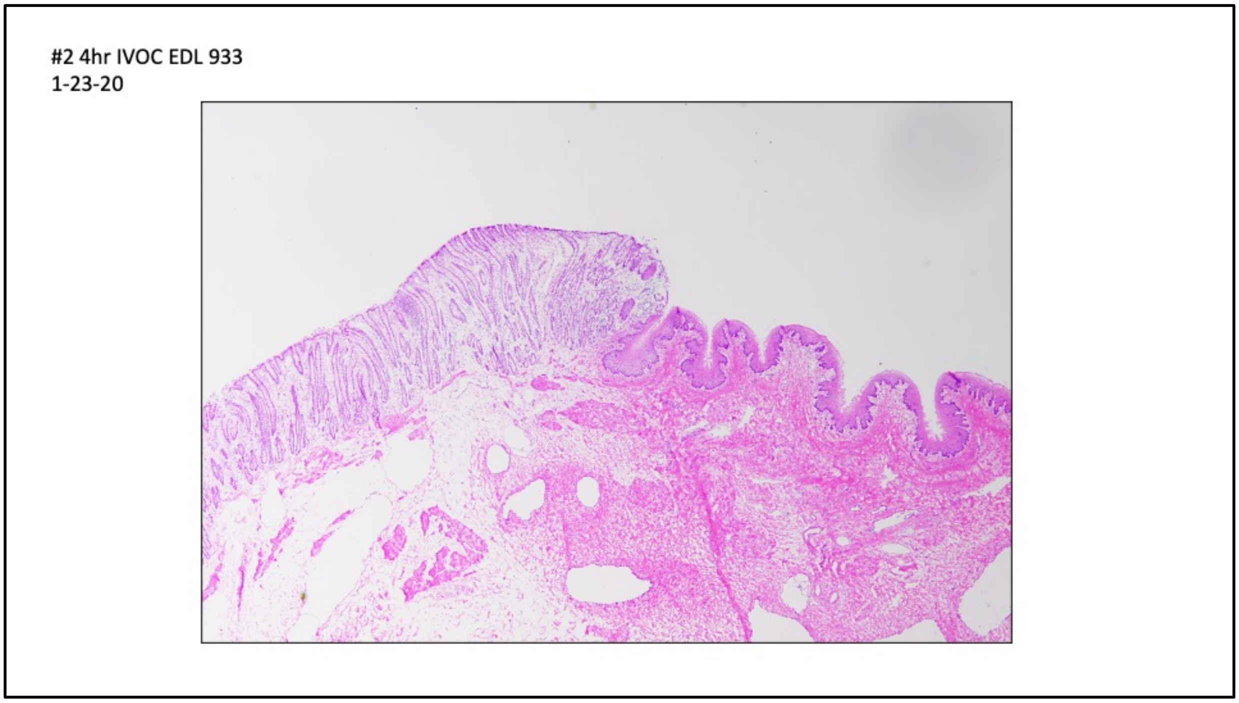

#2 4hr IVOC EDL 933
1-23-20
This is a section of rectoanal junction with both glandular and squamous portions present. Epithelium of both portions appears normal.

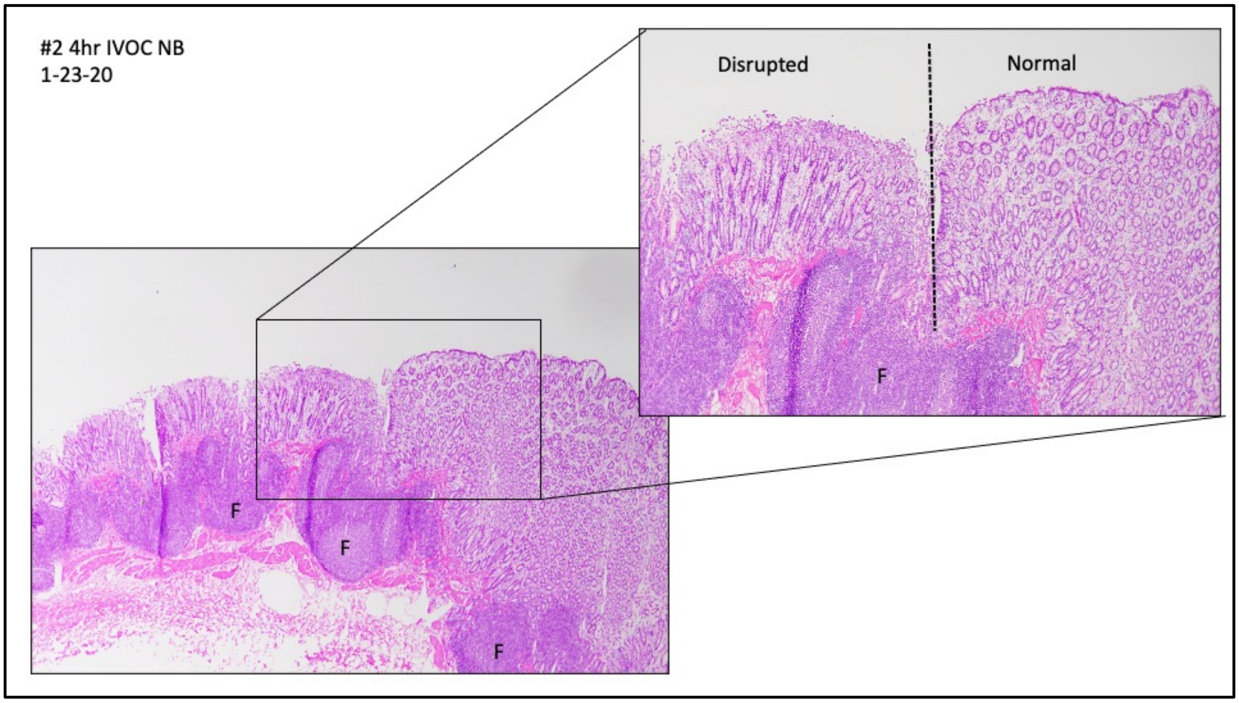

#2 4hr IVOC NB
1-23-20
This is a section of rectoanal junction with both glandular and squamous portions present. Approximately 50% of the glandular epithelium is disrupted. The remaining glandular epithelium and the squamous epithelium appear normal. A portion of the glandular region has underlying lymphoid follicles (F) and therefore represents lymphoepithelium.
