# Supplementary material for: Bovine Rectoanal Junction In Vitro Organ Culture Model System to Study Shiga Toxin-Producing Escherichia coli Adherence
Source: Microorganisms. 2023 May 15;11(5):1289. doi: 10.3390/microorganisms11051289 (PMC10220872; doi:10.3390/microorganisms11051289)
Supplement: Supplementary file 1 [file microorganisms-11-01289-s001.zip › Supp.Data-Histopathology Reports/Supp.Data-HistopathologyReport-S2-Trials 4-Comp10^8-7-22-21 and 7-29-21-II.pdf]

Dr. Indira Kudva

Histopathology Results
IVOC samples, 7-22-21 and 7-29-21
NB-A, B and C, ATCC 43895, ATCC 43895- A and B,
K12-A and B
by
Dr. Mitchell Palmer
8-26-2021

Summary

- All NB samples of rectoanal junction have both squamous and glandular portions present. All have mucosa that is normal in appearance with intact epithelium.
- The primary and only change in all other specimens was mucosal disruption of the glandular region epithelium to varying degrees.
- Squamous regions on all slides were unremarkable.
- ATCC 43895B had small colonies of short bacilli or mixed bacterial morphology associated with mucus or other debris, which was present near or attached to the mucosal surface.

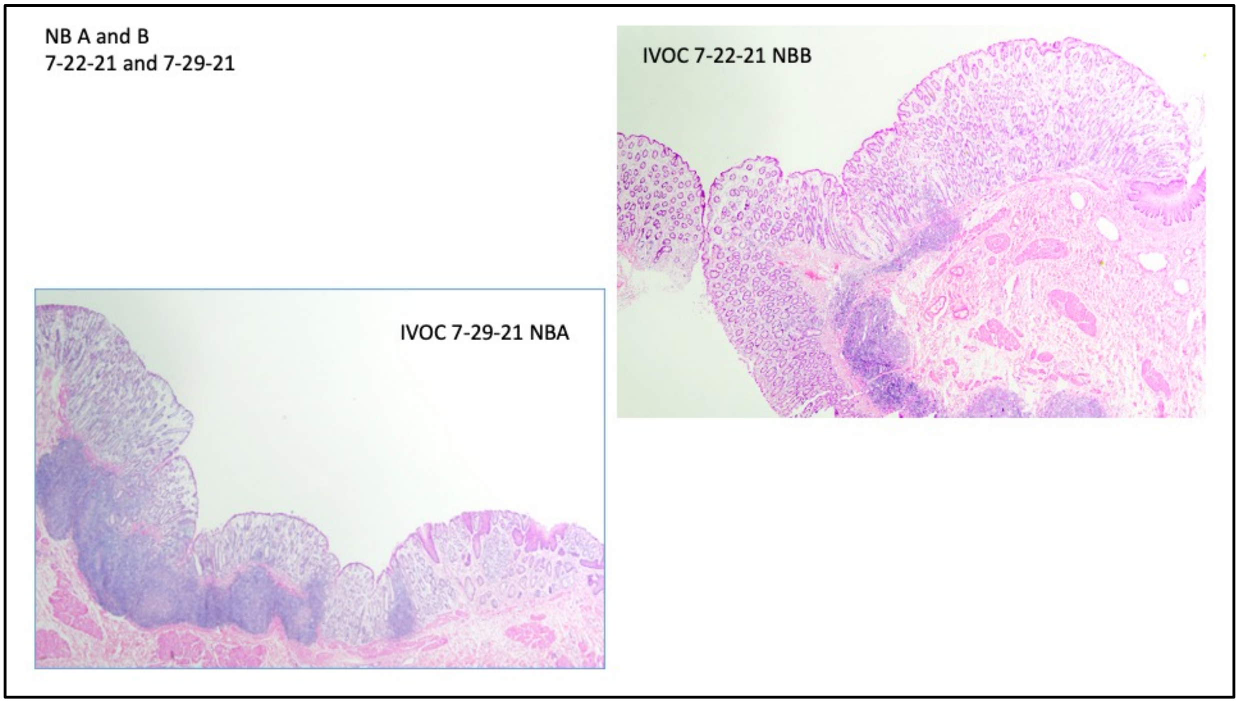

NB A and B 7-22-21 and 7-29-21
These are sections of rectoanal junction with both glandular and squamous regions present. The glandular mucosa is intact. The squamous portion is unremarkable.

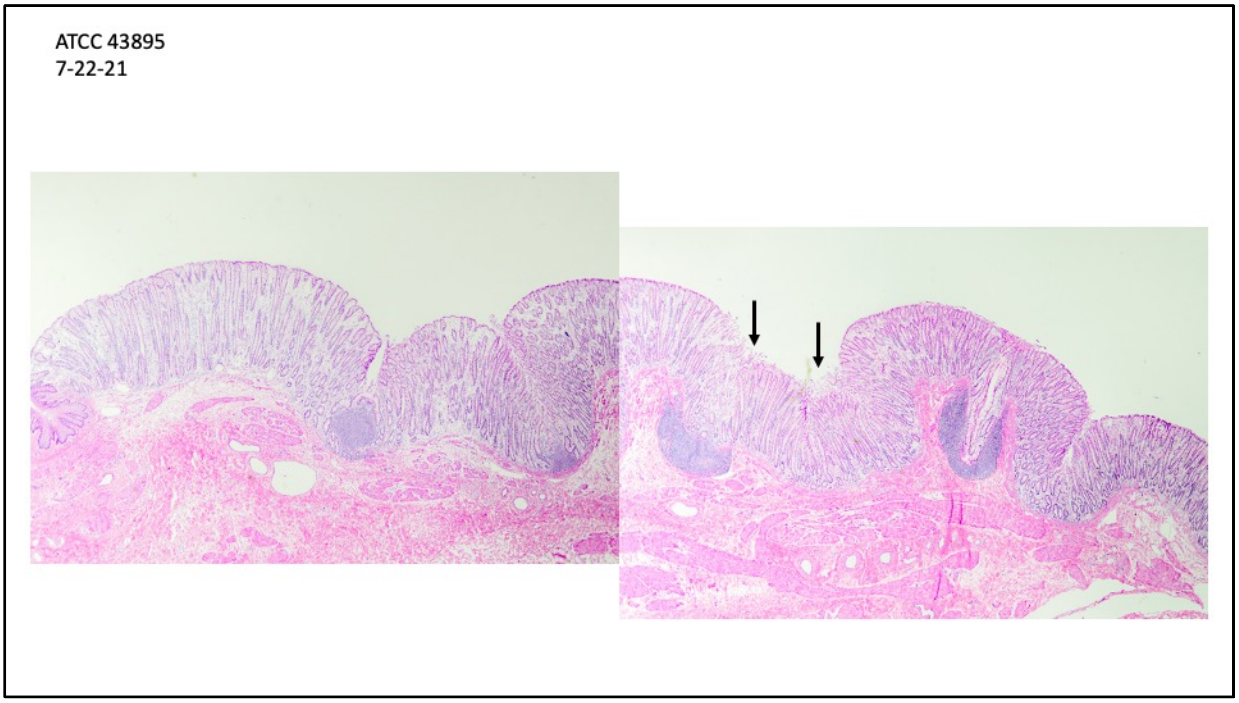

ATCC 43895 (7-22-21)
This is a section of rectoanal junction with remnants of both glandular and squamous portions present. Two very small regions of superficial mucosal disruption are present (arrows). The remainder of the epithelium and squamous regions are unremarkable.

ATCC 43895 A
7-29-21

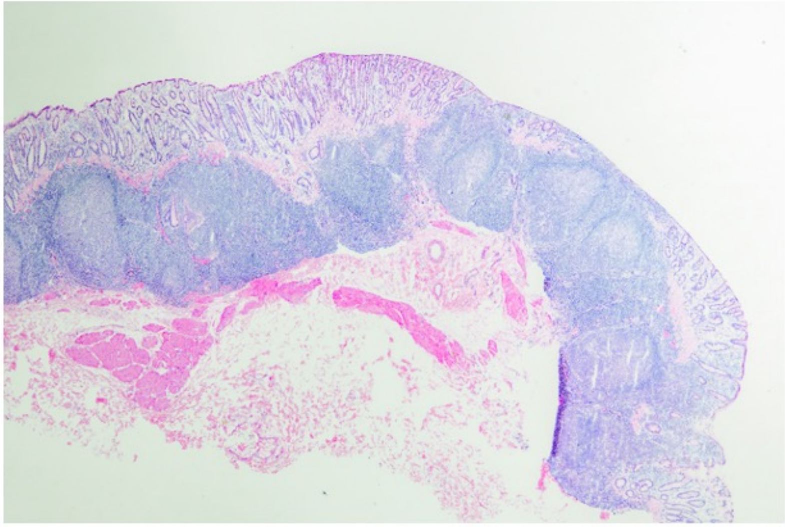

ATCC 43895 A (7-29-21)
This is a section of rectoanal junction with remnants of both glandular and squamous portions present. Both glandular and squamous regions are unremarkable.

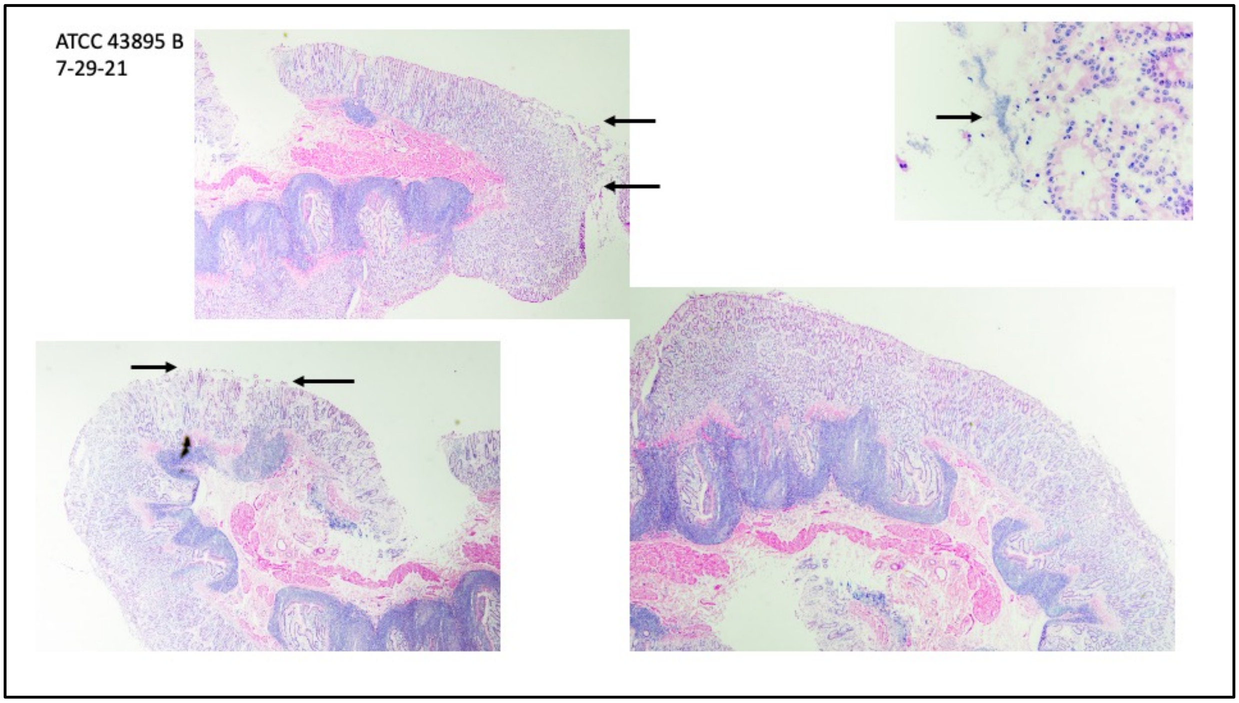

ATCC 43895 B (7-29-21)
This is a section of rectoanal junction with remnants of both glandular and squamous portions present. There is superficial mucosal disruption over approx. 30-40% of the section (between arrows). Colonies of bacteria are visible near the mucosal surface (arrows). The remainder of the epithelium and squamous regions are unremarkable.

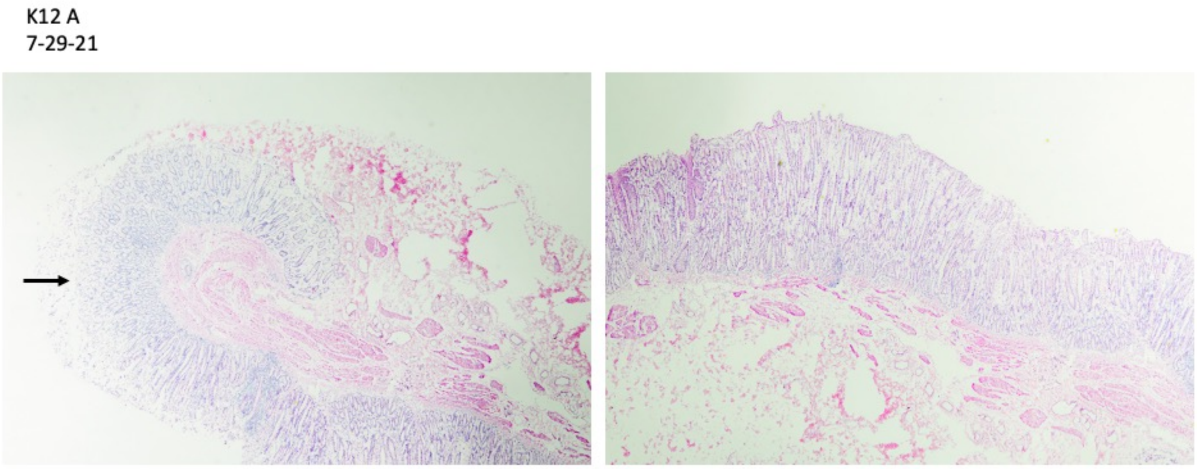

K12 A (7-29-21)
This is a section of rectoanal junction with remnants of both glandular and squamous portions present. There is superficial mucosal disruption over approx. In one area the disruption extends deeper into the lamina propria (arrow). The remainder of the epithelium and squamous regions are unremarkable.

K12 B
7-29-21

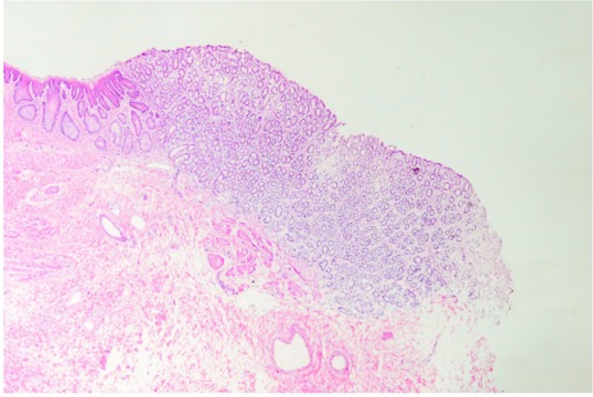

K12 B (7-29-21)
This is a section of rectoanal junction with remnants of both glandular and squamous portions present. There is only a small amount of tissue. The epithelium appears unremarkable.
