# Supplementary material for: Bovine Rectoanal Junction In Vitro Organ Culture Model System to Study Shiga Toxin-Producing Escherichia coli Adherence
Source: Microorganisms. 2023 May 15;11(5):1289. doi: 10.3390/microorganisms11051289 (PMC10220872; doi:10.3390/microorganisms11051289)
Supplement: Supplementary file 1 [file microorganisms-11-01289-s001.zip › Supp.Data-Histopathology Reports/Supp.Data-HistopathologyReport-S3-Comp10^8- 9-27-21.pdf]

Dr. Indira Kudva

Histopathology Results
IVOC samples, 9-27-21
NB A&B, K12 A&B, ATCC 43895 A and B
by
Dr. Mitchell Palmer

Summary

- All NB samples of rectoanal junction have both squamous and glandular portions present. All have mucosa that is normal in appearance with intact epithelium.
- All K12 samples of rectoanal junction have both squamous and glandular portions present. There is a very small area of mucosal disruption on K12B that could be due to tissue handling.
- All ATCC 43895 samples of rectoanal junction have both squamous and glandular portions present. There is a very small area of mucosal disruption on A that is likely due to tissue handling. There is a very small area of mucosal disruption on slide B.
- Squamous regions on all slides were unremarkable.

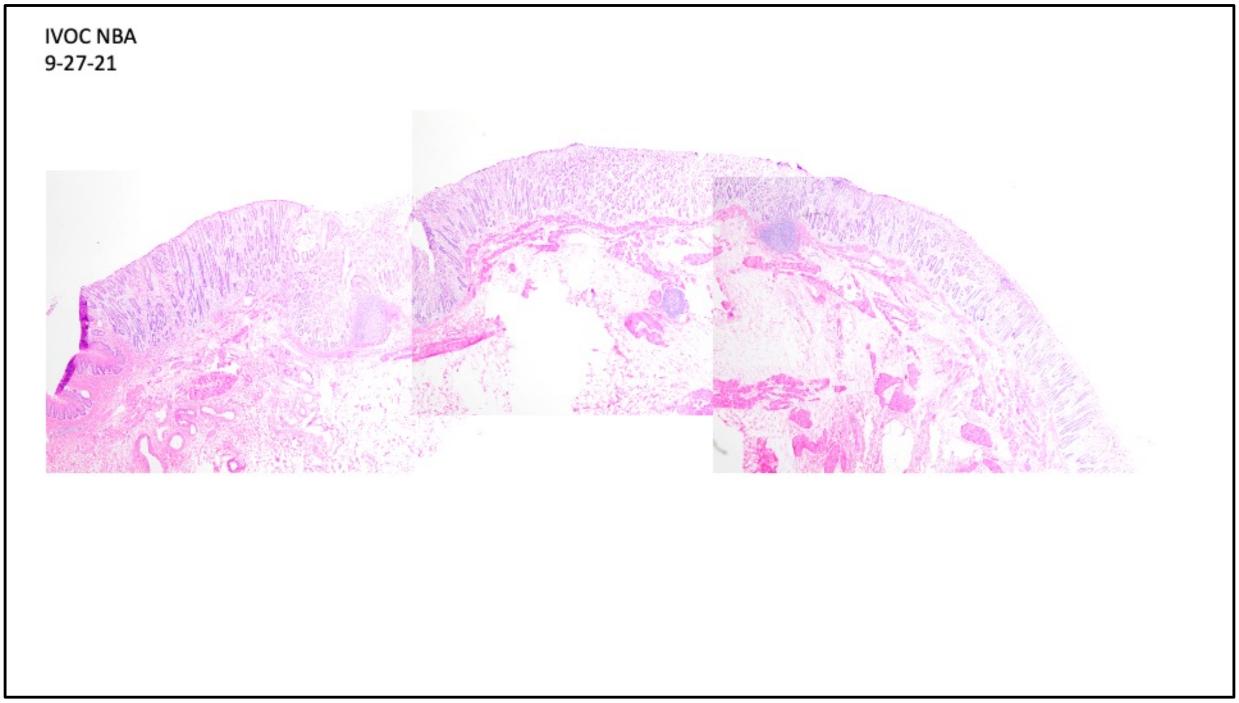

NBA 9-27-21

These are sections of rectoanal junction with both glandular and squamous regions present. The glandular mucosa is intact. The squamous portion is unremarkable.

IVOC NBB
9-27-21

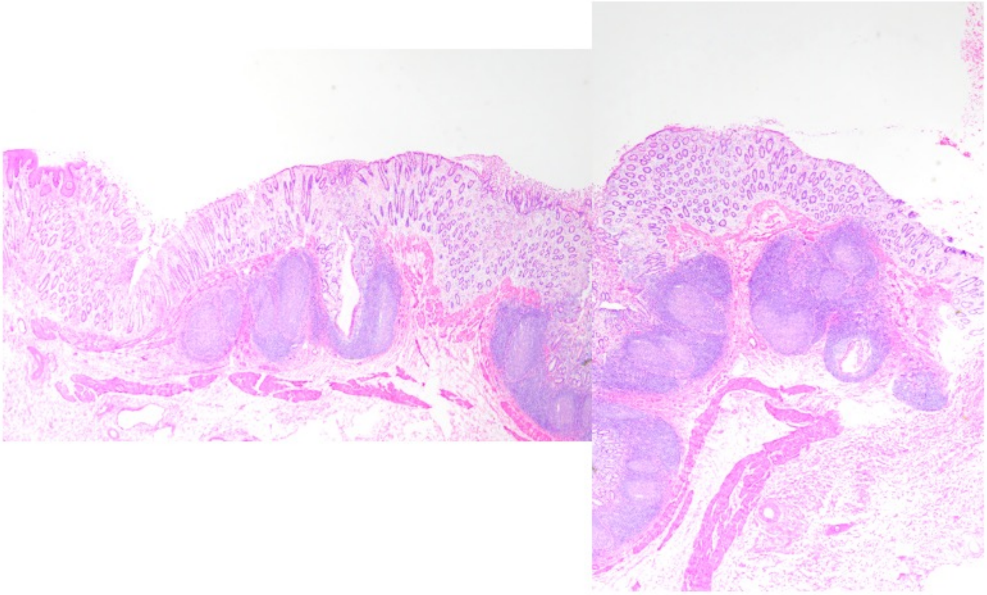

NBB 9-27-21
These are sections of rectoanal junction with both glandular and squamous regions present. The glandular mucosa is intact. The squamous portion is unremarkable.

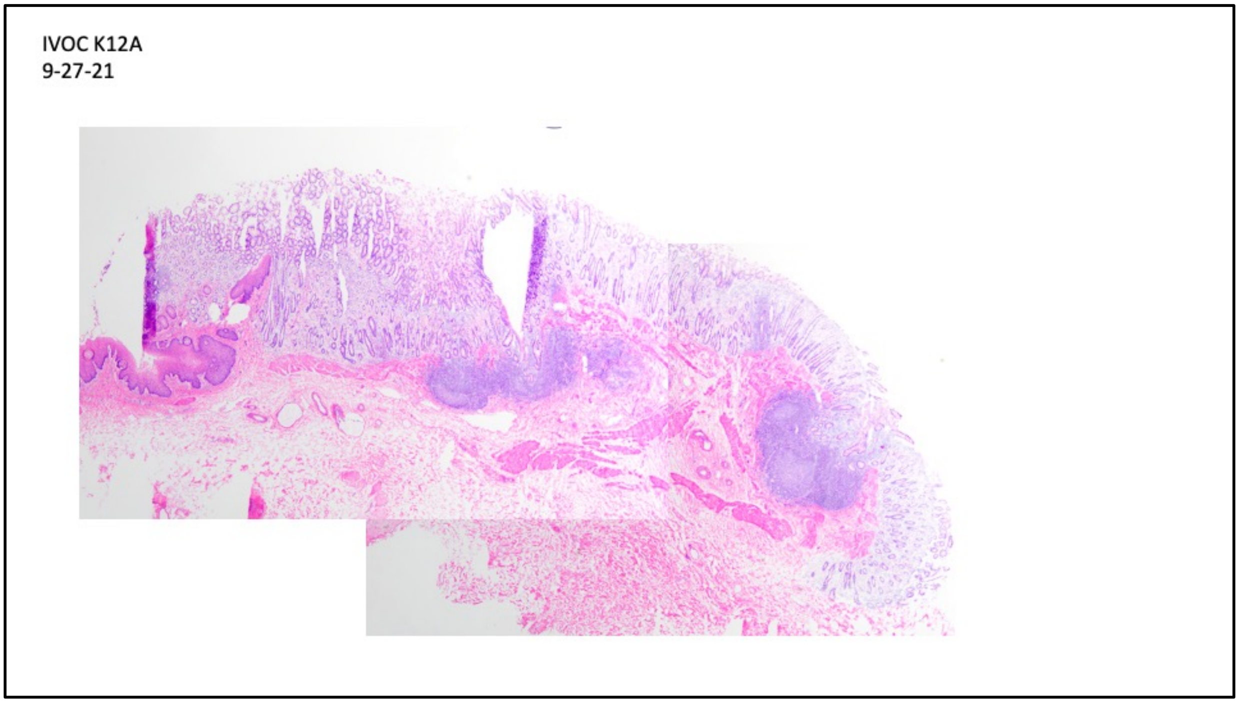

K12A (9-27-21)

These are sections of rectoanal junction with both glandular and squamous regions present. The glandular mucosa is intact. The squamous portion is unremarkable.

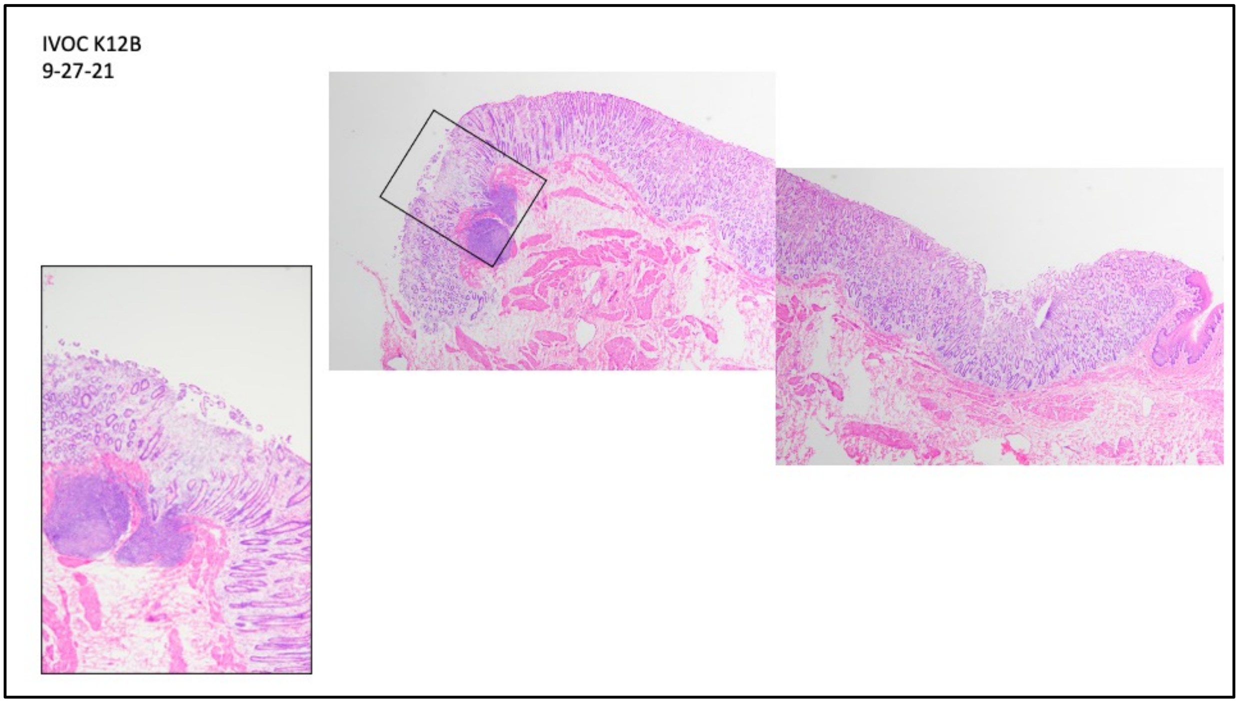

K12B (9-27-21)
These are sections of rectoanal junction with both glandular and squamous regions present. There is a small area of very mild disruption toward the end of the section. This could be artifact due to tissue handling. The squamous portion is unremarkable.

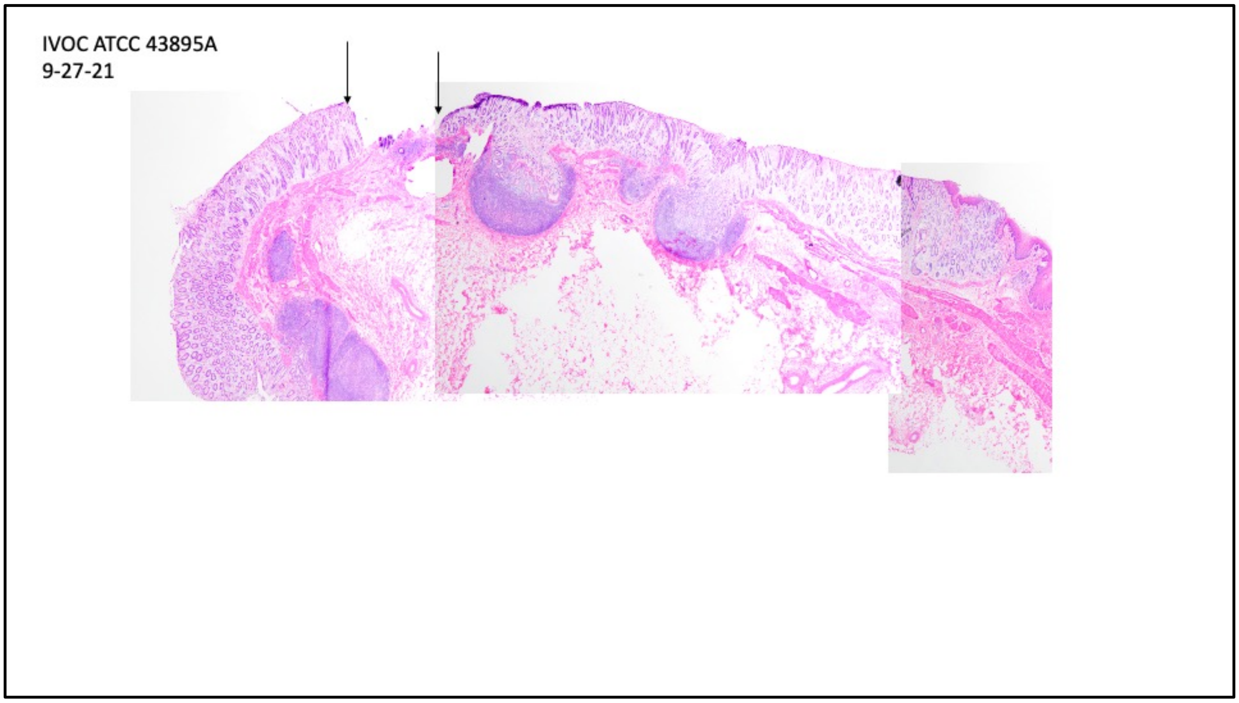

ATCC 43895A (9-27-21)
These are sections of rectoanal junction with both glandular and squamous regions present. There is a small area of very mild disruption (arrows). The sharply demarcated nature of this area suggests that this could be artifact due to tissue handling. The squamous portion is unremarkable.

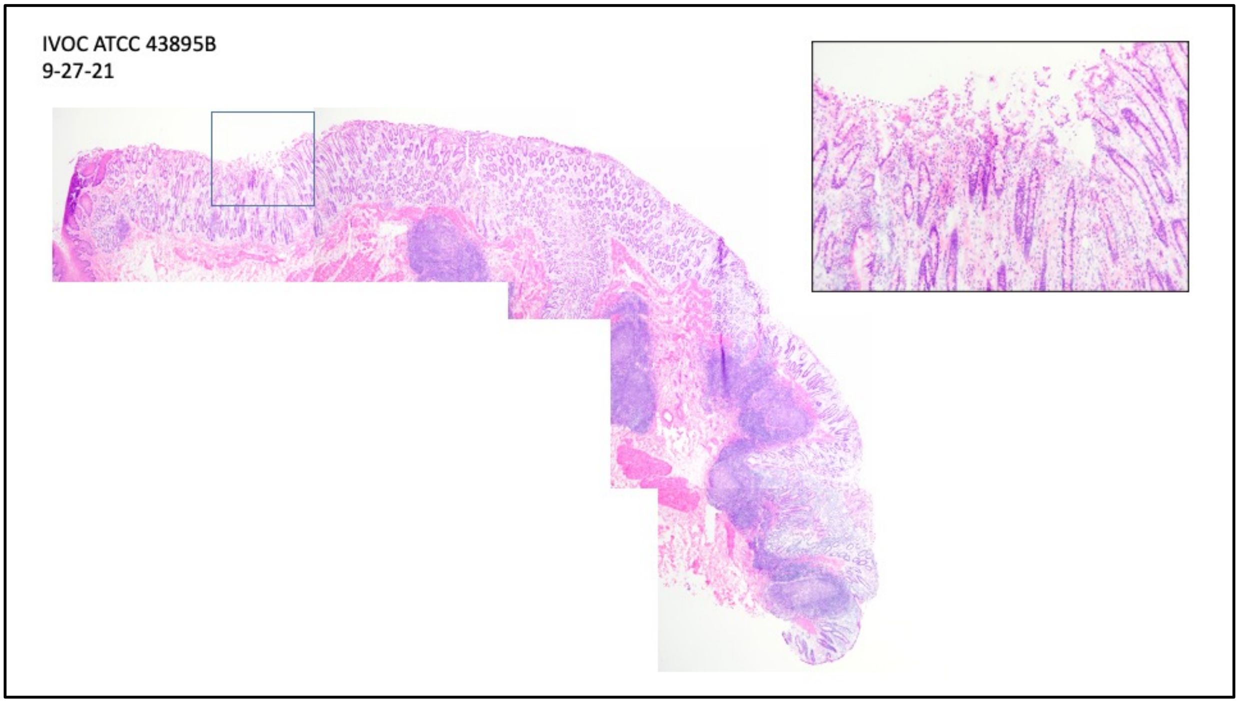

ATCC 43895B (9-27-21)
These are sections of rectoanal junction with both glandular and squamous regions present. There is a small area of very mild disruption (box). The squamous portion is unremarkable.
