# Supplementary material for: Bovine Rectoanal Junction In Vitro Organ Culture Model System to Study Shiga Toxin-Producing Escherichia coli Adherence
Source: Microorganisms. 2023 May 15;11(5):1289. doi: 10.3390/microorganisms11051289 (PMC10220872; doi:10.3390/microorganisms11051289)
Supplement: Supplementary file 1 [file microorganisms-11-01289-s001.zip › Supp.Data-Histopathology Reports/Supp.Data-HistopathologyReport-S4-Comp10^8-11-10-2021.pdf]

Dr. Indira Kudva

Histopathology Results
IVOC samples from 11-10-21
by
Dr. Mitchell Palmer

Summary- 11/10/21

- Slide set 11/10/21
  - Slides with GALT hyperplasia. In many cases where there were submucosal follicles, lymphocytes extended from the submucosa and in the lamina propria lymphocytes extended toward superficial mucosa.
  - All squamous regions were normal except EDL 933 B.
  - Pretty normal NB and K12 with NB A having a small region of mucosal disruption in columnar region.
  - EDL 933: A has significant disruption with part of the superficial layer detached in columnar region. B has an interesting are of attenuation and cell infiltrates in the squamous portion.

11/10/21: NB

- Squamous portion of RAJ (both A and B): normal
- Columnar portion of RAJ:
  - NB A- small focus of superficial mucosal disruption
  - NB B- normal
  - Both NB A and B have moderate to prominent GALT hyperplasia with lymphocytes extending from the submucosa superficially towards the superficial mucosal.

11/10/21: K12

- Squamous portion of RAJ (both A and B): normal
- Columnar portion of RAJ:
  - K12 A: normal mucosa. Prominent GALT hyperplasia with lymphocytes extending from the submucosa superficially towards the superficial mucosal. Single folding artifact.
  - K12 B: normal mucosa. Prominent GALT hyperplasia with lymphocytes extending from the submucosa superficially towards the superficial mucosal.

11/10/21 : EDL 933

- Squamous portion of RAJ:
  - EDL 933 A: normal
  - EDL 933 B: small region of attenuation with subjacent cellular infiltrates involving approximately 10% of the length of the mucosa.
- Columnar portion of RAJ:
  - EDL 933 A: there is superficial mucosal disruption involving approximately 40% of the length of the mucosa, with a significant portion of superficial mucosa lifted off from the remaining mucosal layer.
  - EDL 933 B: normal, GALT with lymphocytes extending superficially from submucosa towards mucosa.

NB A
11/10/21

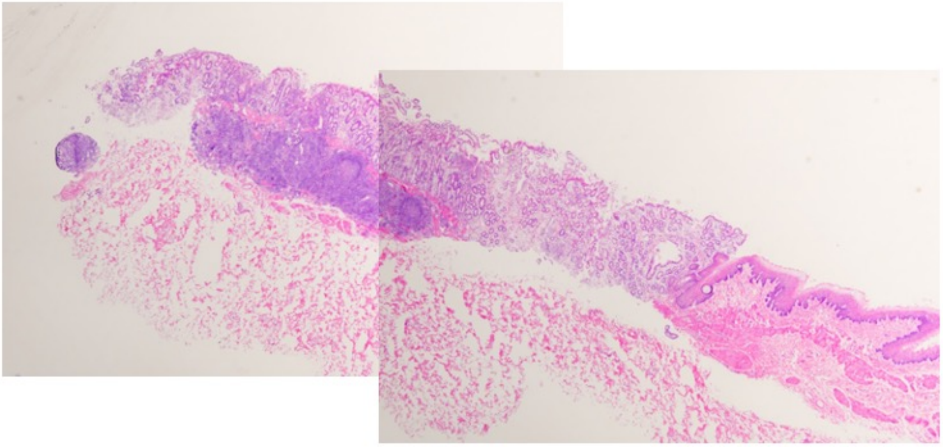

Squamous portion of RAL: normal
Columnar portion of RAJ: normal

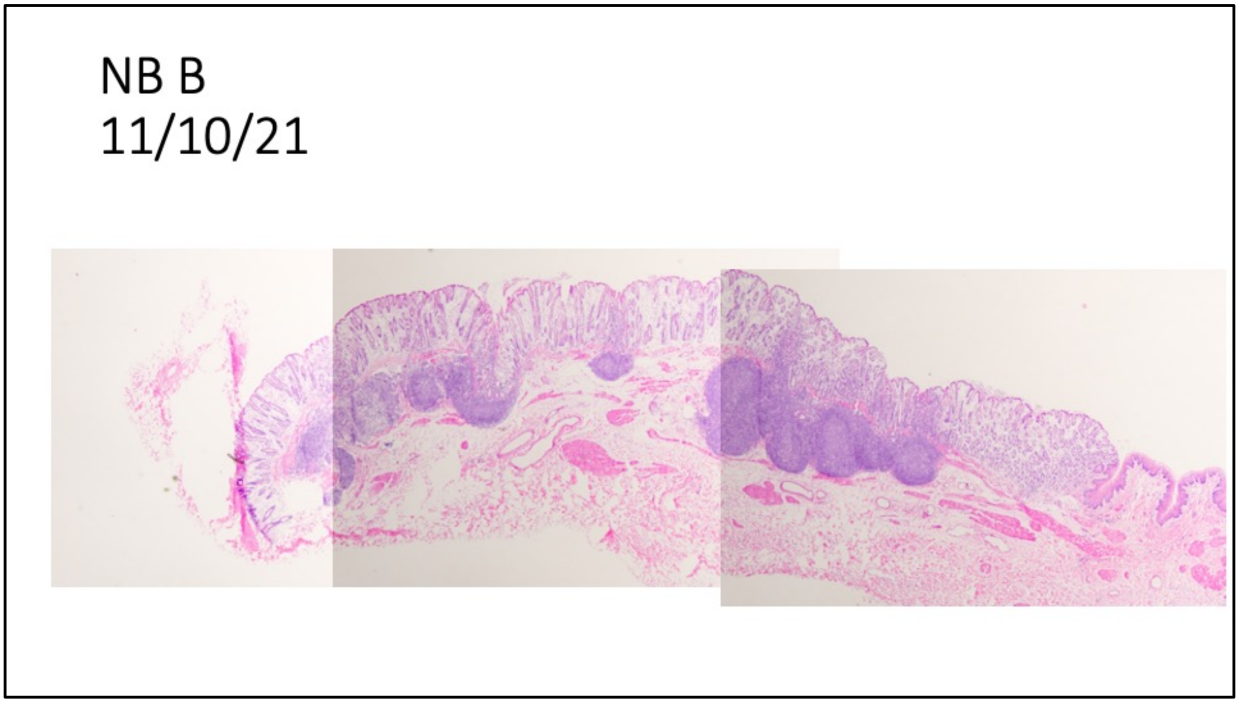

Squamous portion of RAL: normal
Columnar portion of RAJ: normal
Prominent GALT hyperplasia with multiple foci extending superficially through lamina propria

K12 A
11/10/21

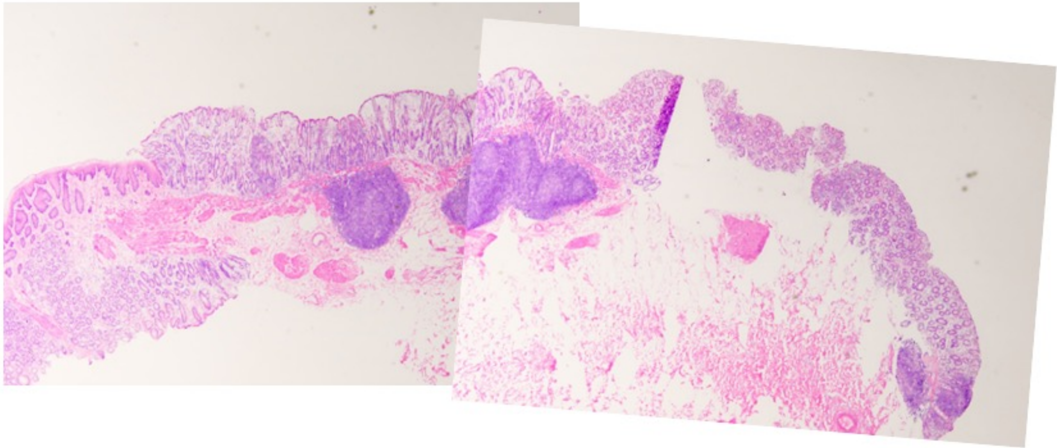

Squamous portion of RAL: normal
Columnar portion of RAJ: normal
Moderate GALT hyperplasia with foci extending superficially into lamina propria.
Single folding artifact.

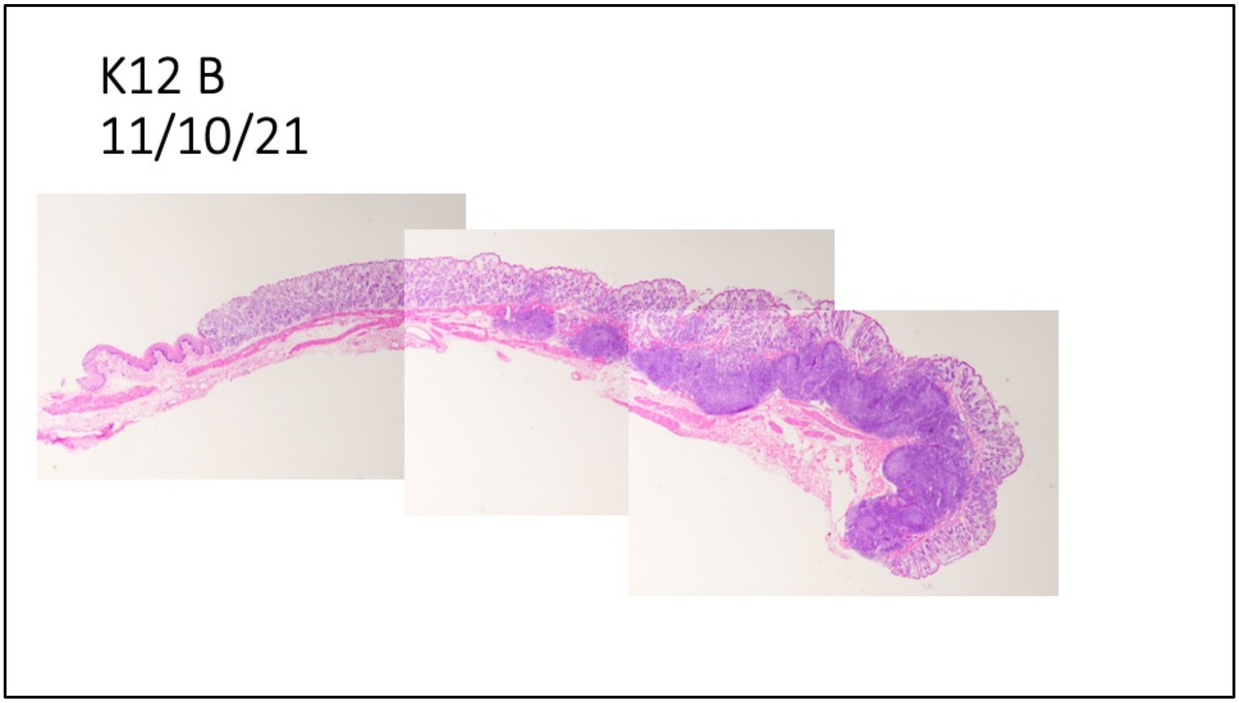

Squamous portion of RAL: normal
Columnar portion of RAJ: normal
Prominent GALT hyperplasia with foci extending superficially into lamina propria.

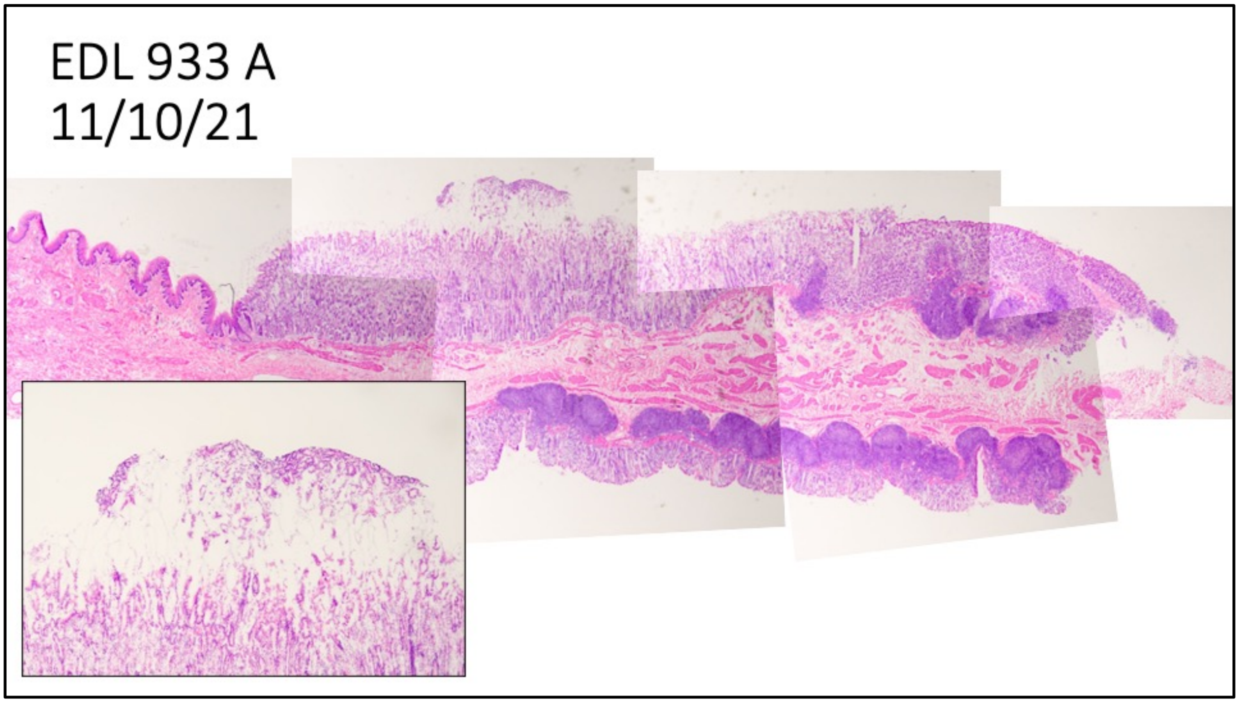

Squamous portion of RAJ: normal
Columnar portion of RAJ: significant mucosal disruption (40%), with some areas extending deeper into lamina propria. Sizeable portion of mucosa lifted off surface. Prominent GALT hyperplasia.

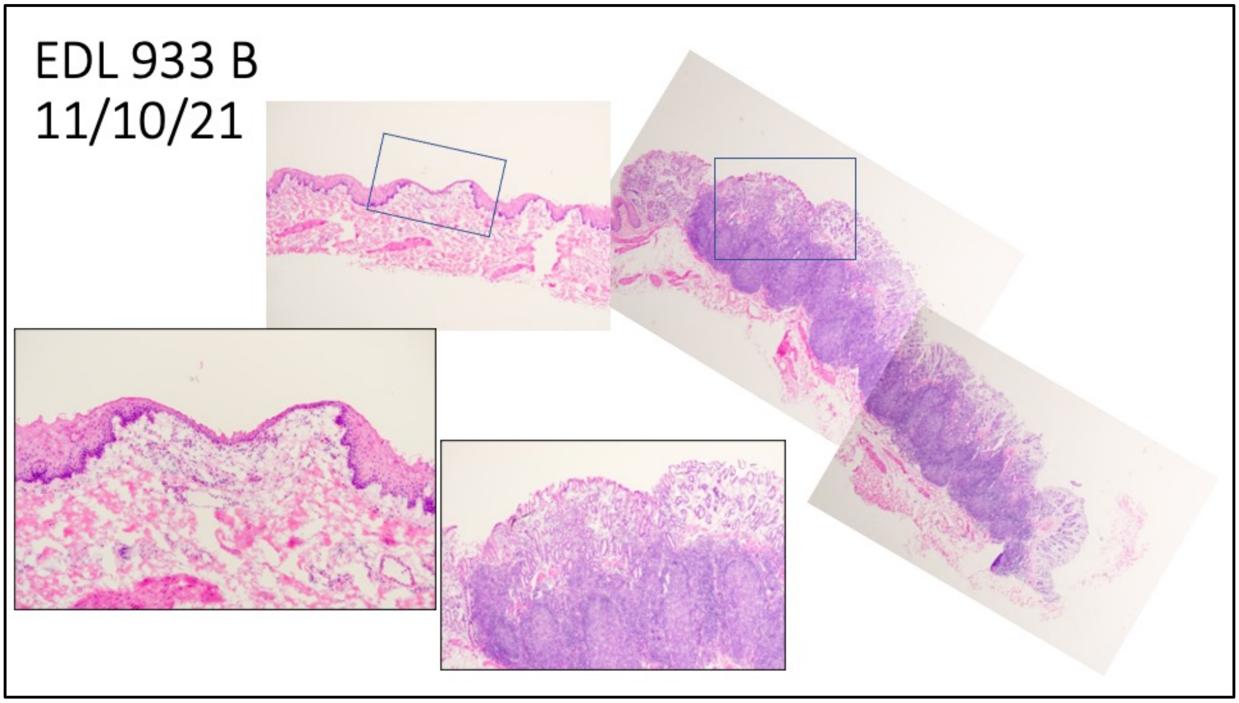

Squamous portion of RAJ: small focus of attenuated mucosa with subjacent, mild infiltrates of cells

Columnar portion of RAJ: multiple small foci of mucosal disruption

Prominent GALT hyperplasia with lymphocytes extending superficially towards mucosal layer
