# Supplementary material for: Bovine Rectoanal Junction In Vitro Organ Culture Model System to Study Shiga Toxin-Producing Escherichia coli Adherence
Source: Microorganisms. 2023 May 15;11(5):1289. doi: 10.3390/microorganisms11051289 (PMC10220872; doi:10.3390/microorganisms11051289)
Supplement: Supplementary file 1 [file microorganisms-11-01289-s001.zip › Supp.Data-Histopathology Reports/Supp.Data-HistopathologyReport-S5-Comp10^7-3.30.22_5.18.22_6 8 22 .pdf]

Dr. Indira Kudva

Histopathology Results
IVOC samples
3-30-22
5-18-22
6/8/22
by
Dr. Mitchell Palmer

Summary

- Pre-assay sections have excellent tissue integrity in all regions.
- Of the sections with no bacteria, 2 of them have one to several small areas of superficial mucosal disruption in the columnar region.
- Of the sections with E. coli K12, 3 of them have one to several small areas of superficial mucosal disruption in the columnar region.
- Two sections containing ATCC/EDL933 have significant superficial mucosal disruption affecting 25->50% of length of the mucosa; other sections have multiple superficial mucosal disruptions.
- Squamous regions on all slides were unremarkable.

3-30-22
Pre-assay

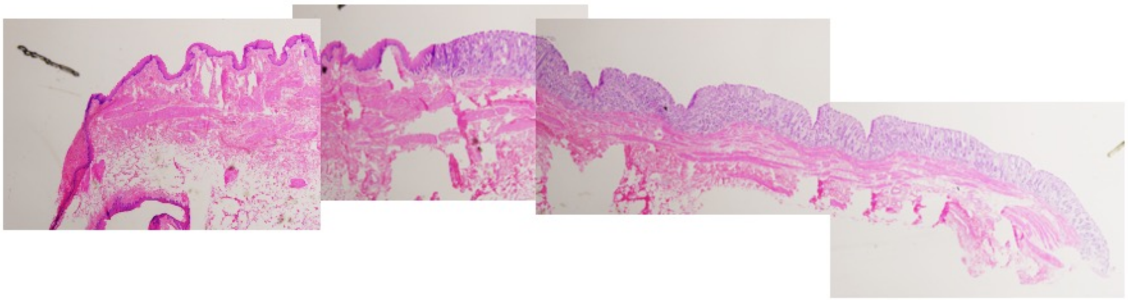

3-30-22
Pre-assay
Excellent tissue integrity in all regions.

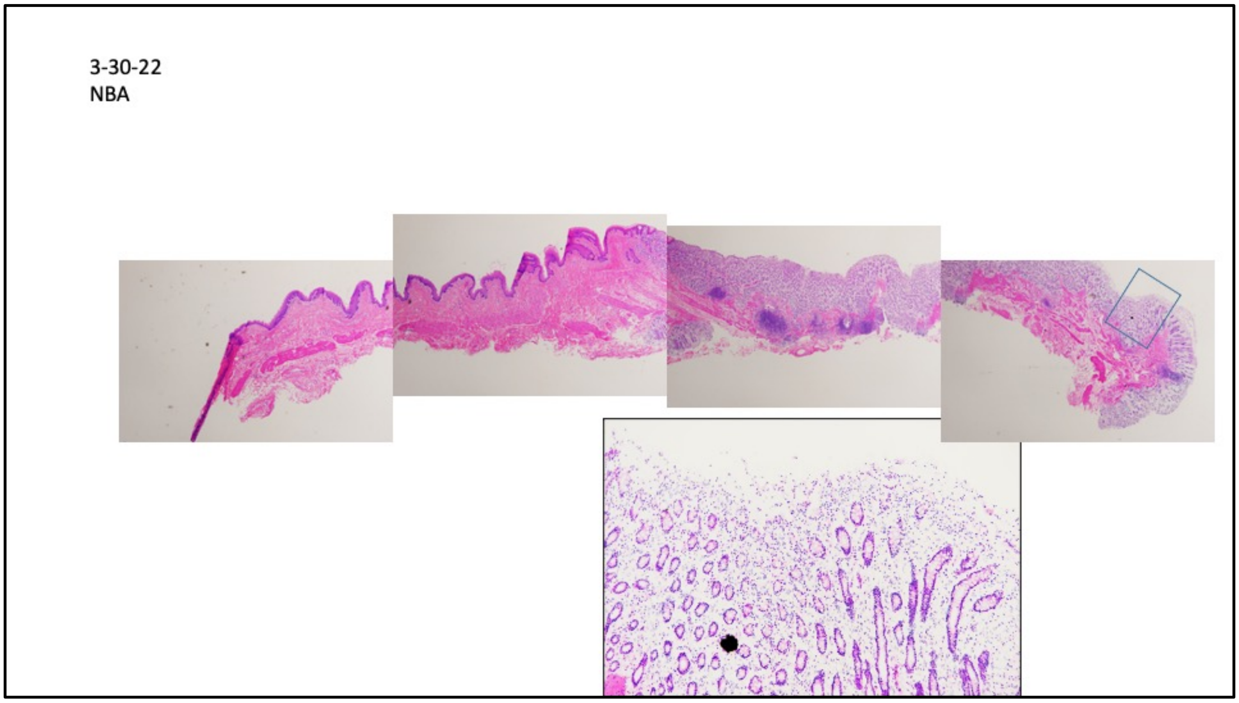

3-30-22
NBA
Small superficial disruption of mucosal epithelium.
Moderate amount of GALT.
Normal squamous region.

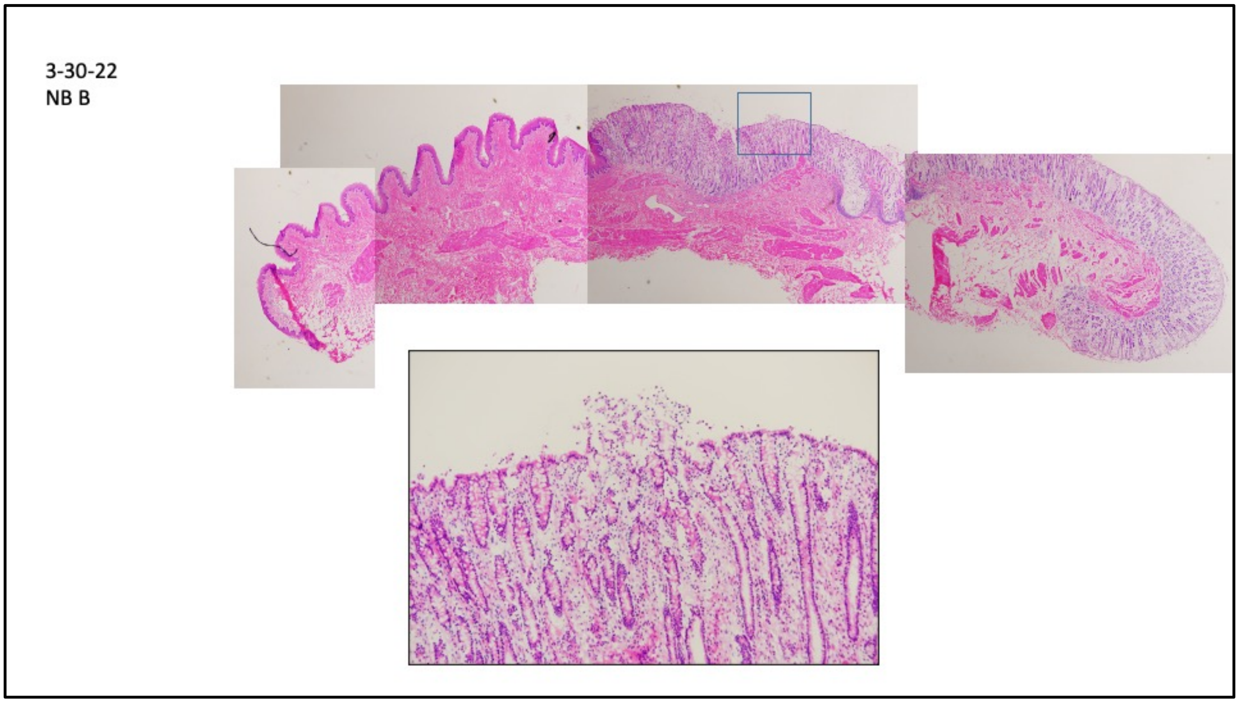

3-30-22
NB B
Several small superficial disruptions of mucosal epithelium in columnar region.
Moderate amounts of GALT.
Normal squamous region.

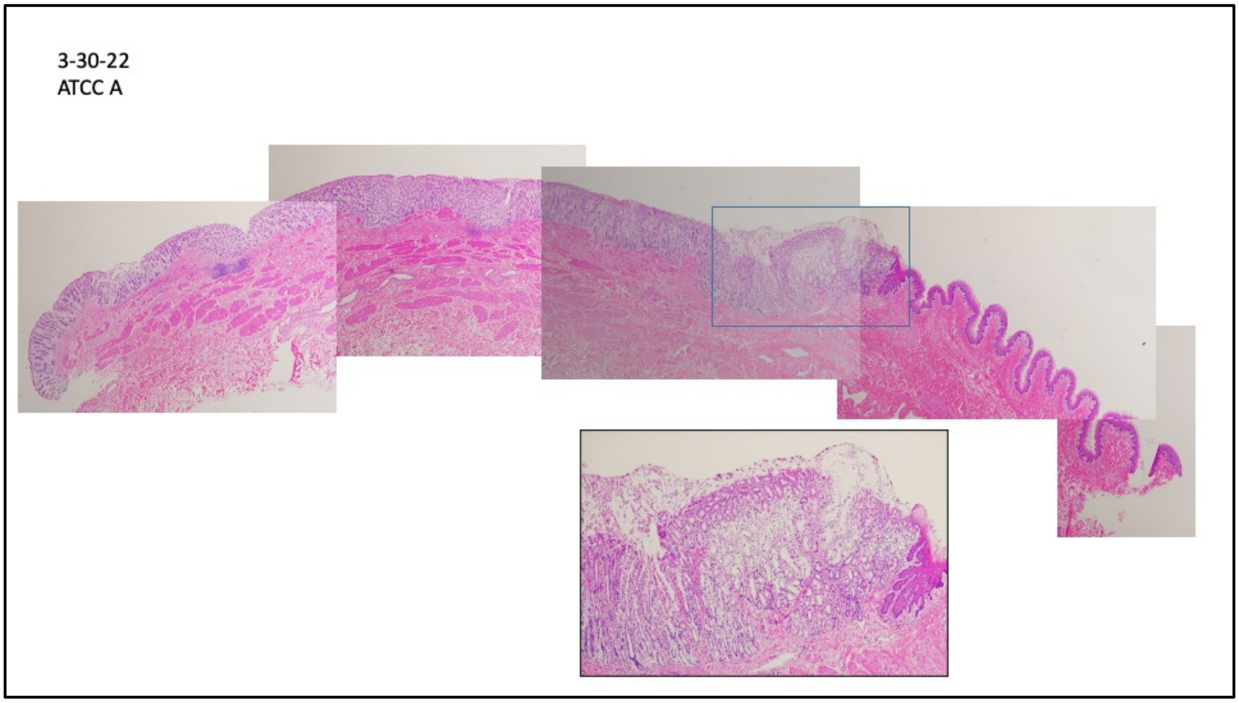

3-30-22
ATCC A
Multiple superficial to mid-level regions of mucosal disruption in columnar region.
Small amount of GALT.
Normal squamous region.

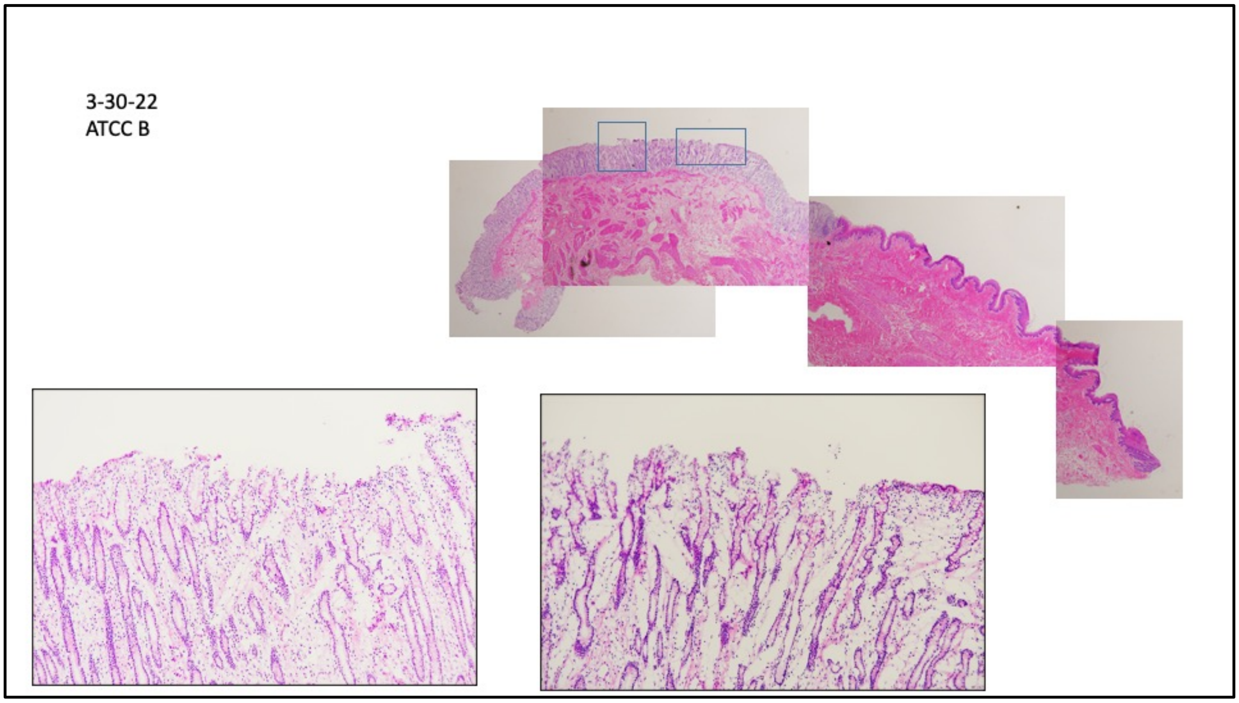

3-30-22
ATCC B
Multiple small areas of superficial mucosal disruption in columnar region.
Small amount of GALT.
Normal squamous region.

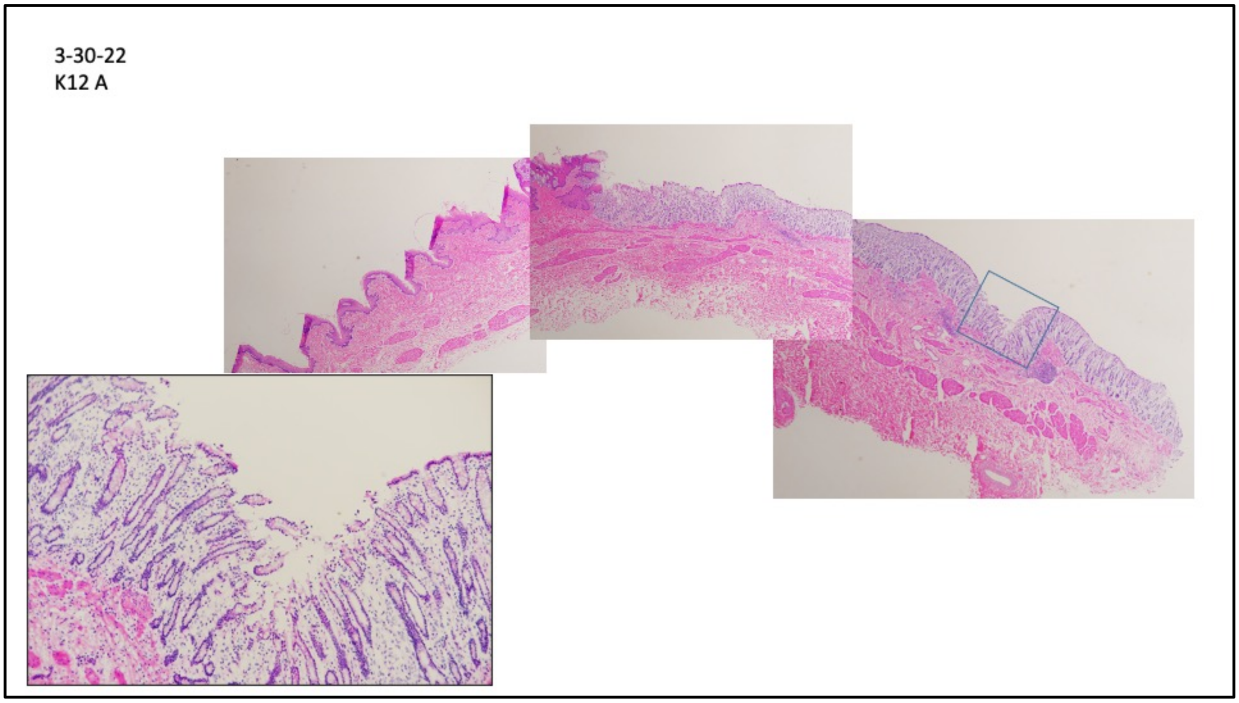

3-30-22
K12 A
Small superficial disruption of mucosa in columnar region.
Small amount GALT
Normal squamous region.

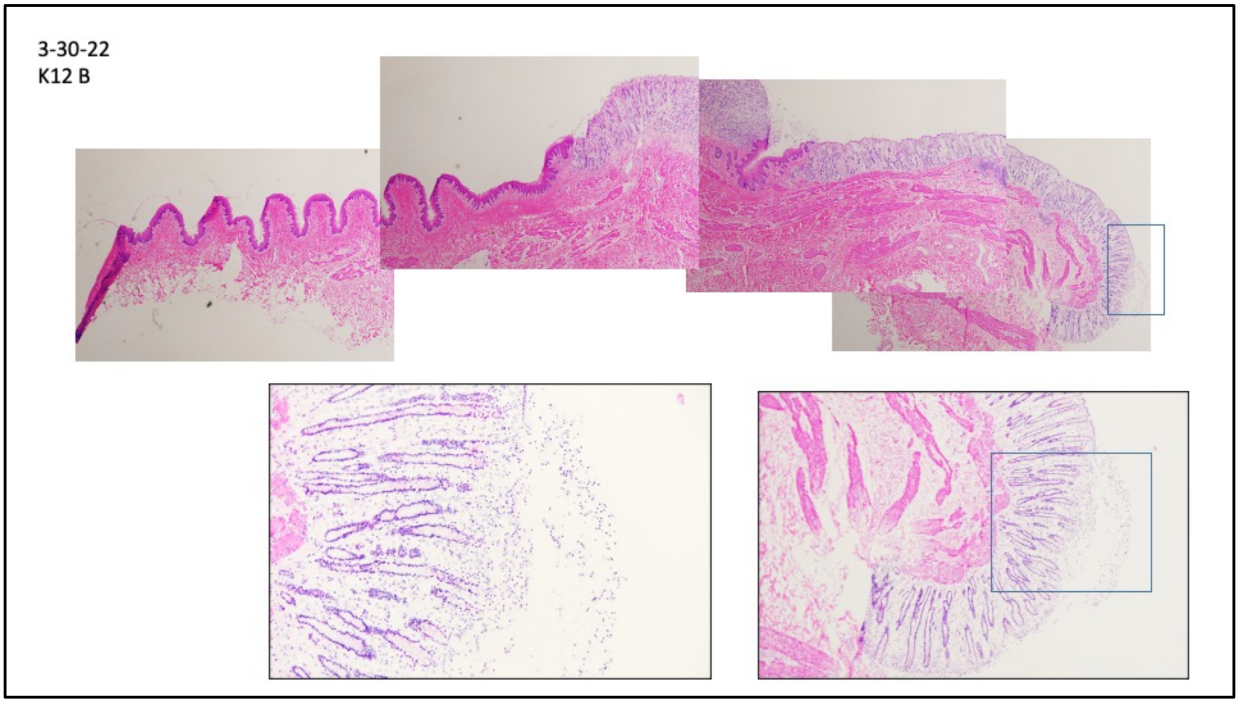

3-30-22
K12 B
Small superficial disruption of mucosa in columnar region.
Small amount of GALT.
Normal squamous region.

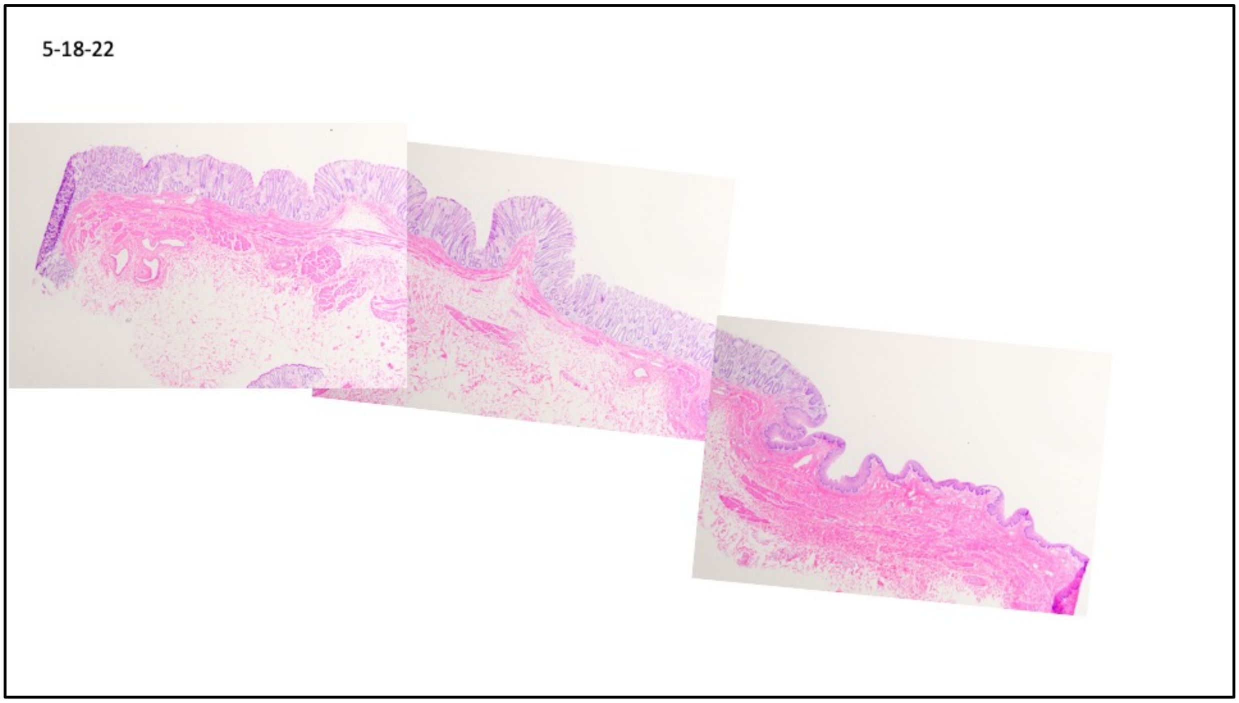

5-18-22
Pre-Assay
Excellent tissue morphology in all regions.

5-18-22
NB-A

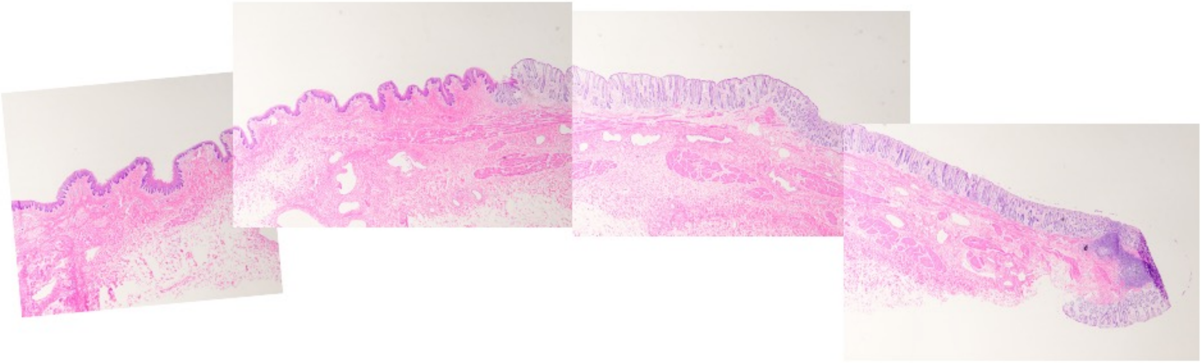

5-18-22
NB-A
Normal in all regions.
Small amount of GALT.

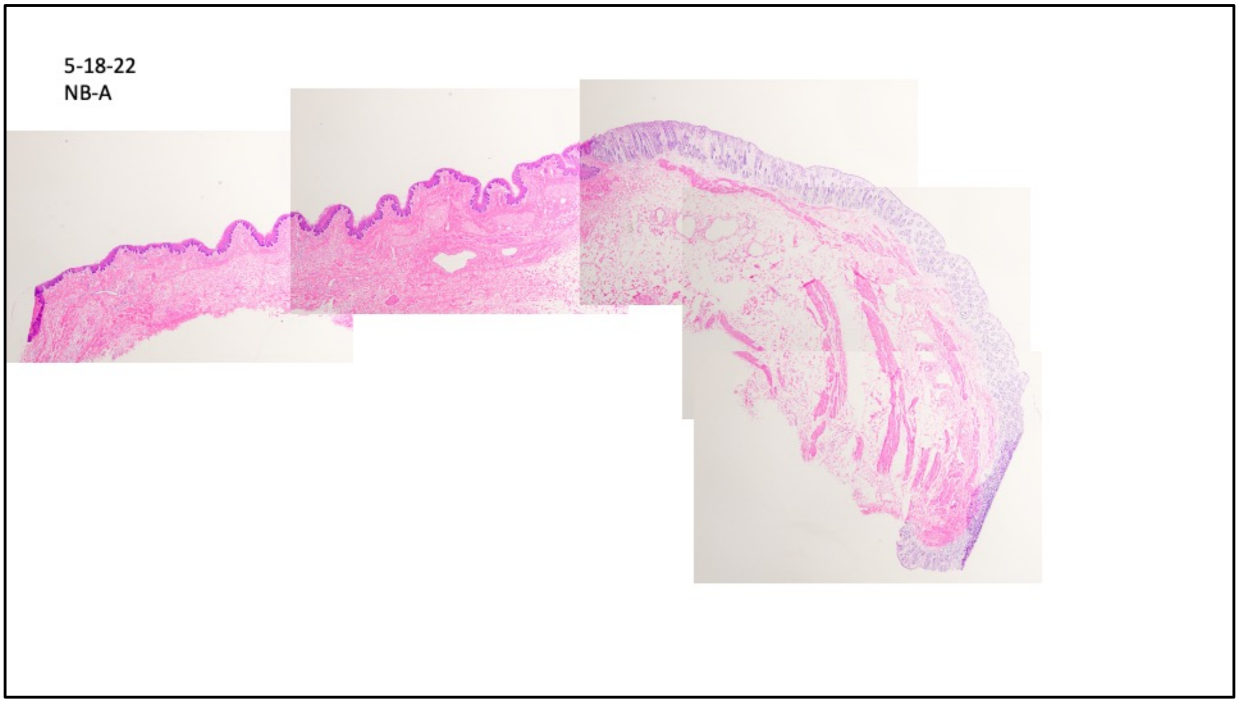

5-18-22
NB-B
Normal in all regions.
No GALT.

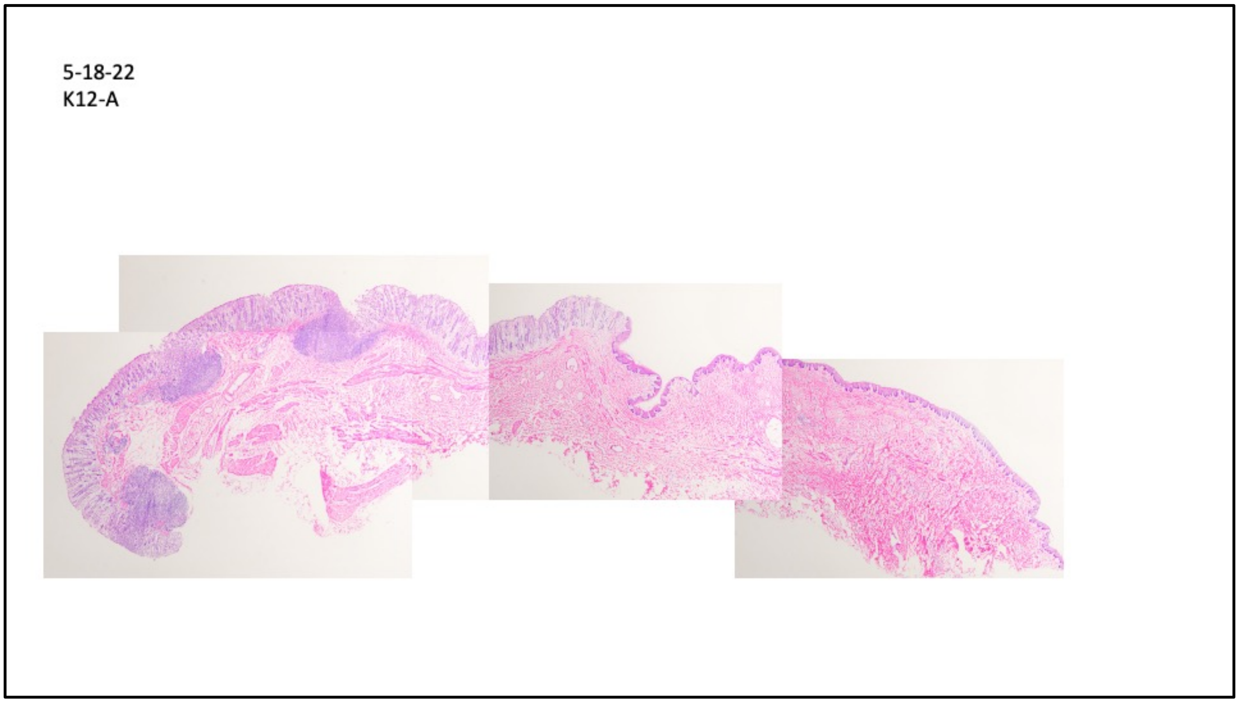

5-18-22
K12- A
Multiple small foci of superficial epithelial disruption.
Moderate to large amount of GALT.
Normal squamous region.

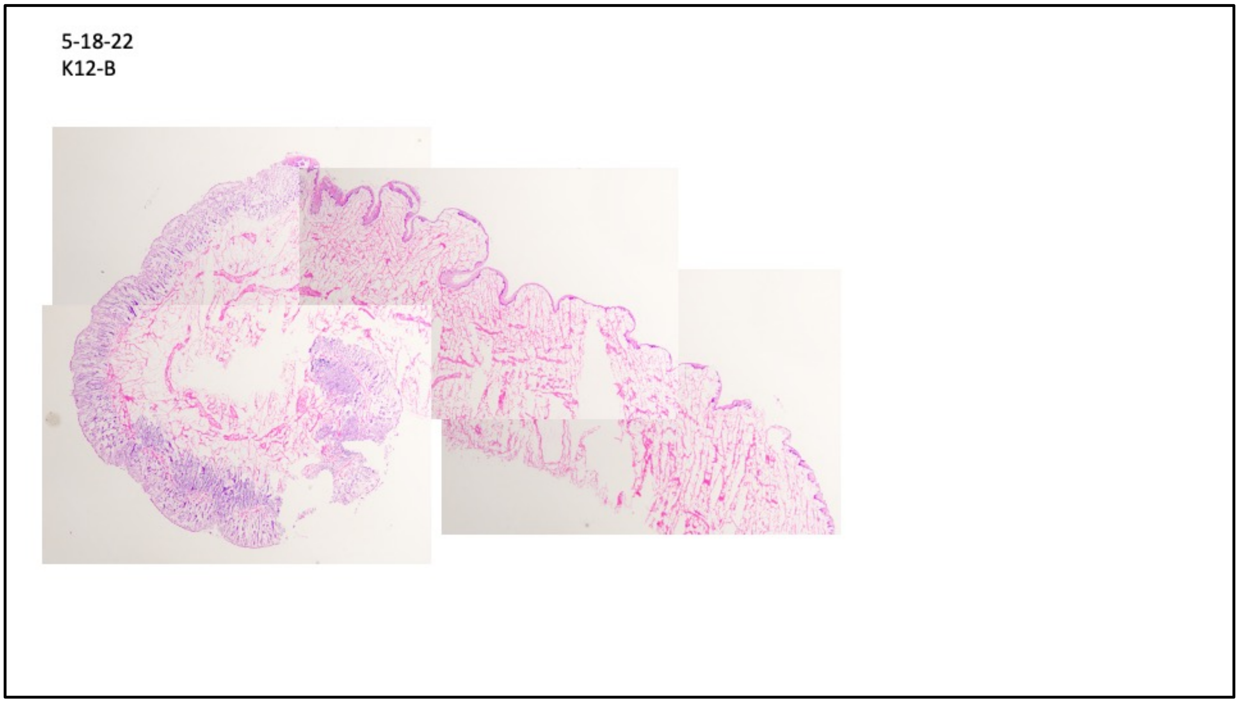

5-18-22
K12-B
Multiple foci of superficial epithelial disruption.
Moderate amount of GALT.
Freezing artifact.

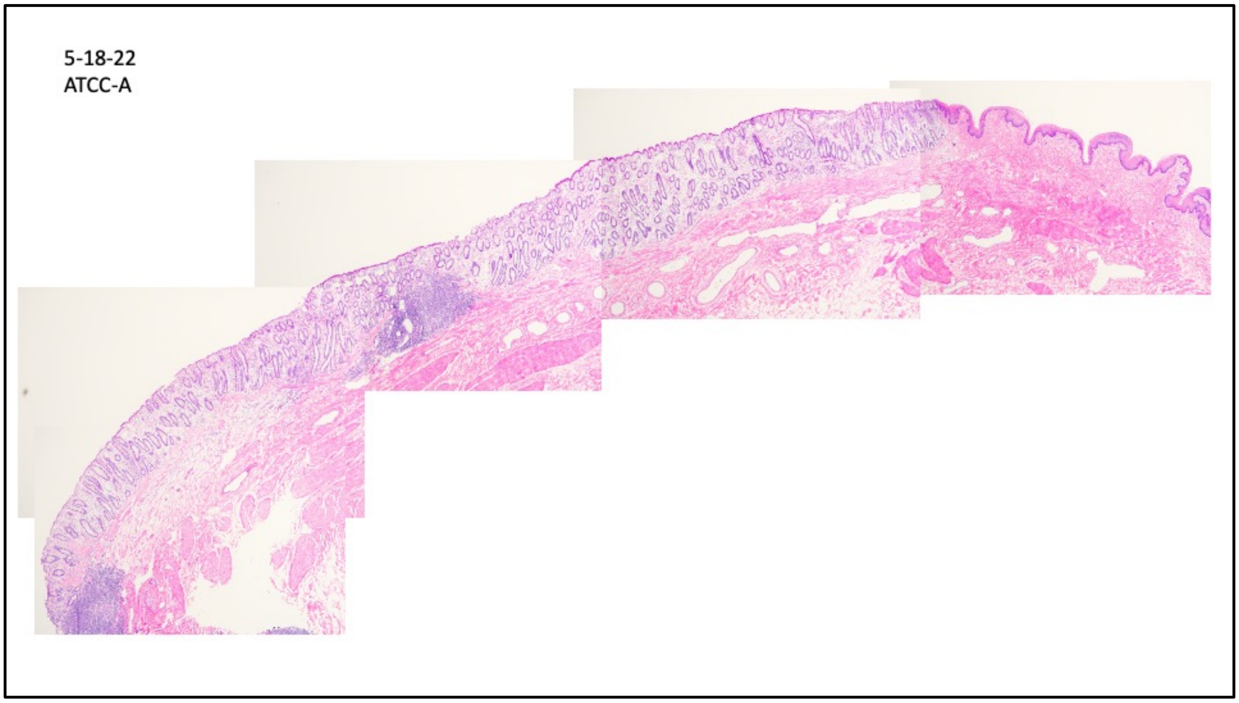

5-18-22
ATCC-A
Normal in all regions.
Small amount of GALT.

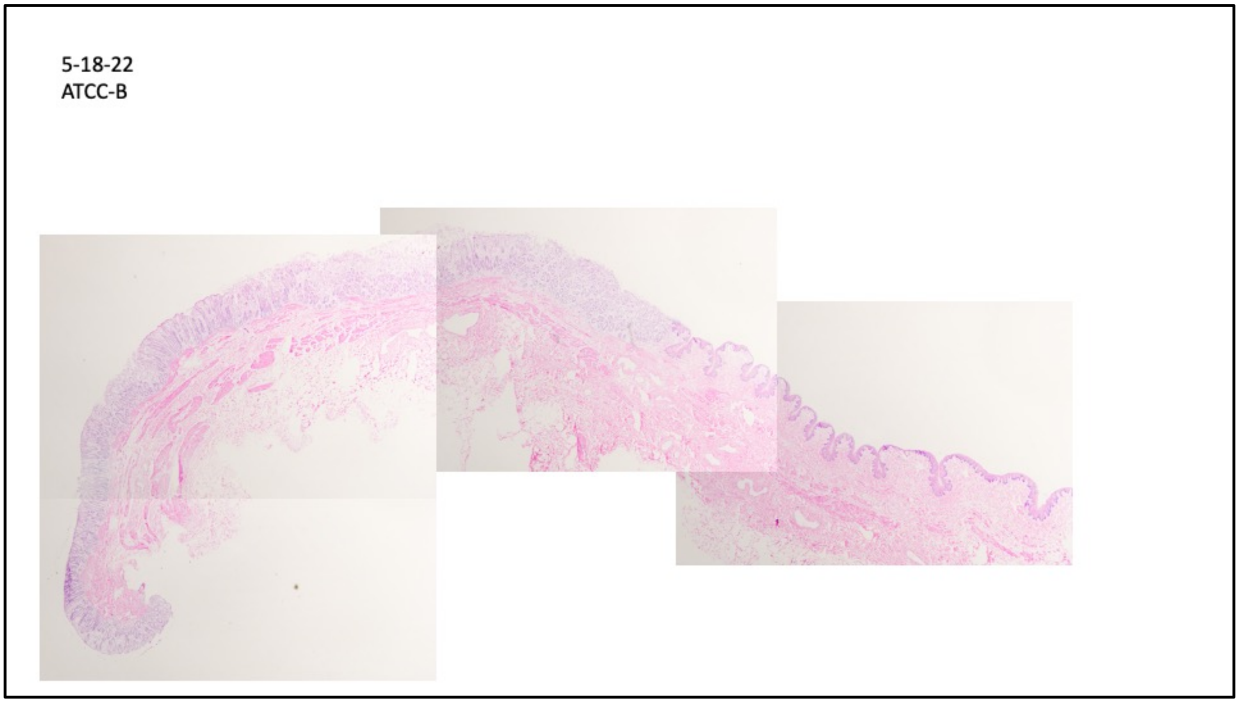

5-18-22
ATCC-B
Multiple foci of epithelial disruption.
No GALT.

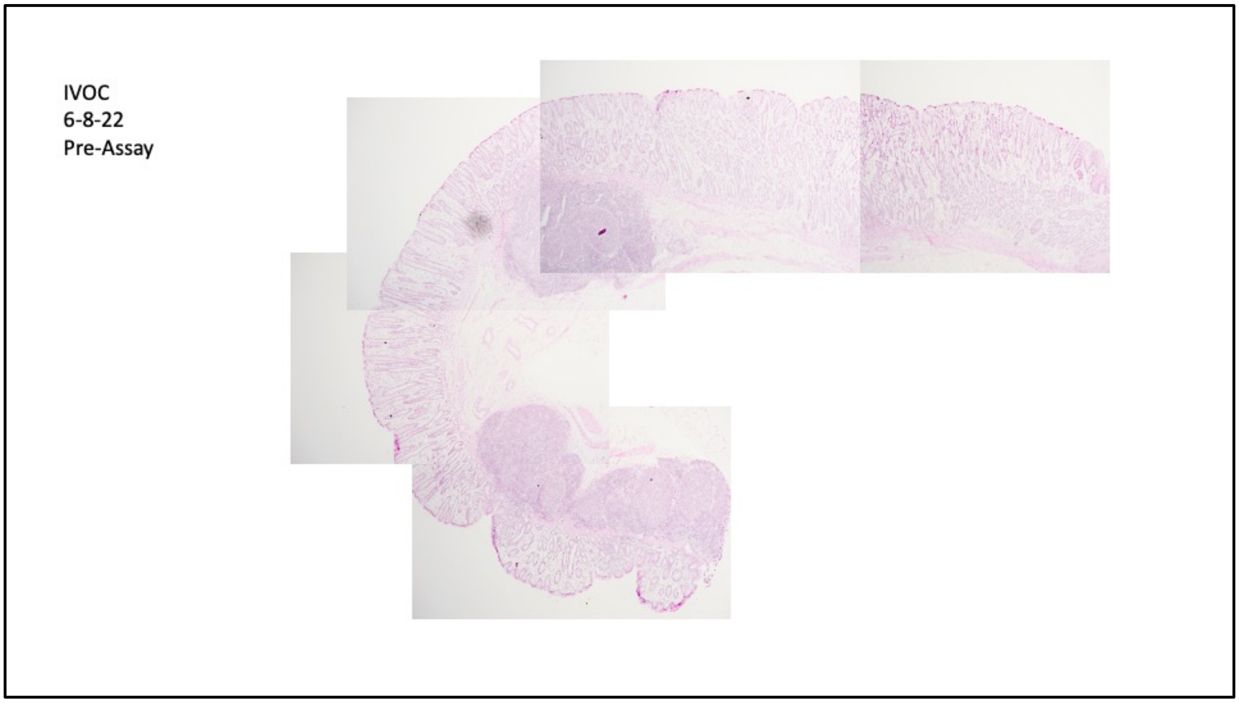

IVOC
6-8-22
Pre-Assay
Excellent tissue integrity in all regions.

IVOC
6-8-22
Pre-Assay

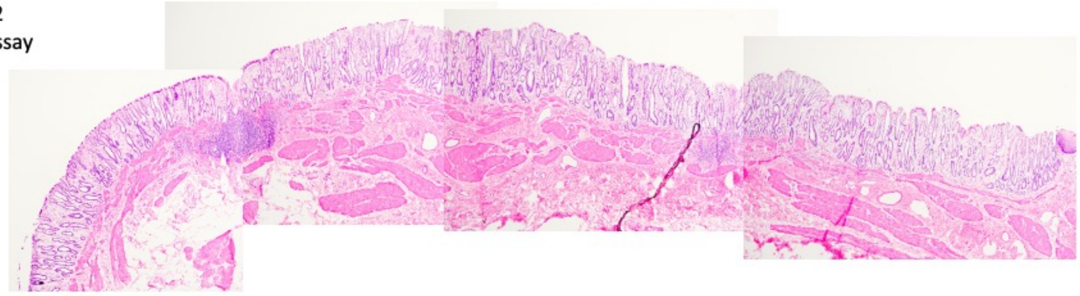

IVOC
6-8-22
Pre-Assay
Excellent tissue integrity in all regions.

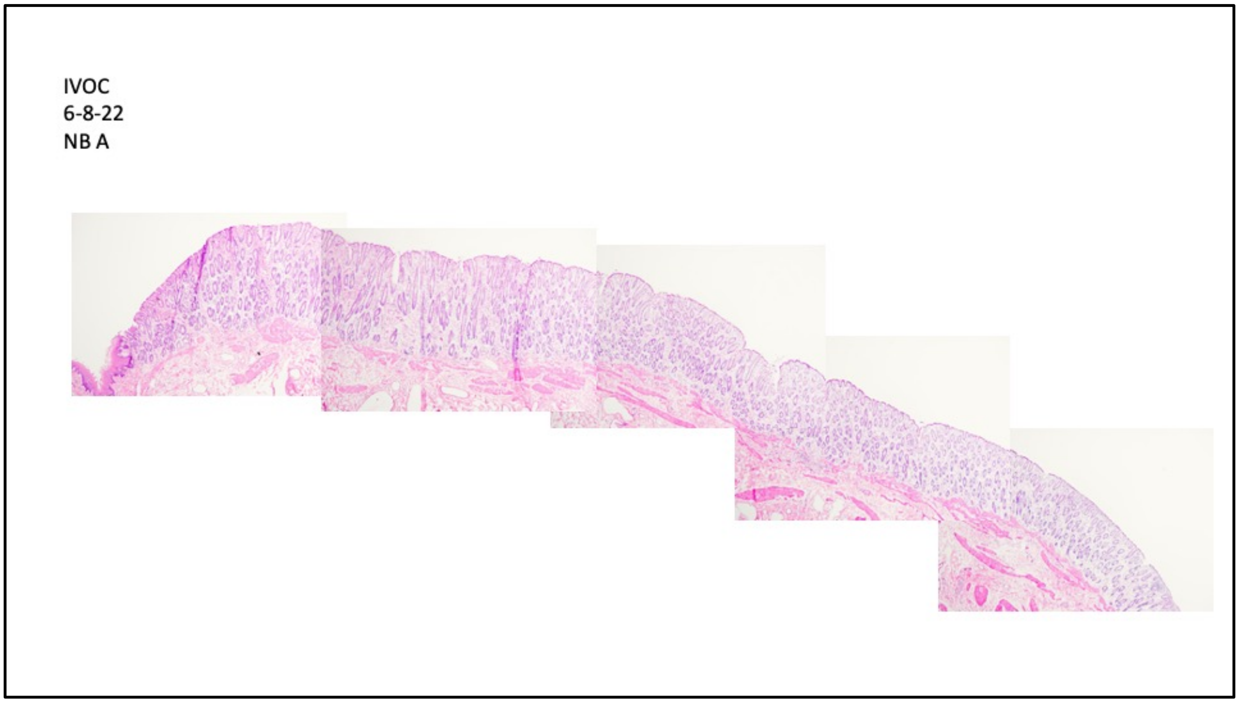

IVOC
6-8-22
NB A
Excellent tissue integrity in all regions.

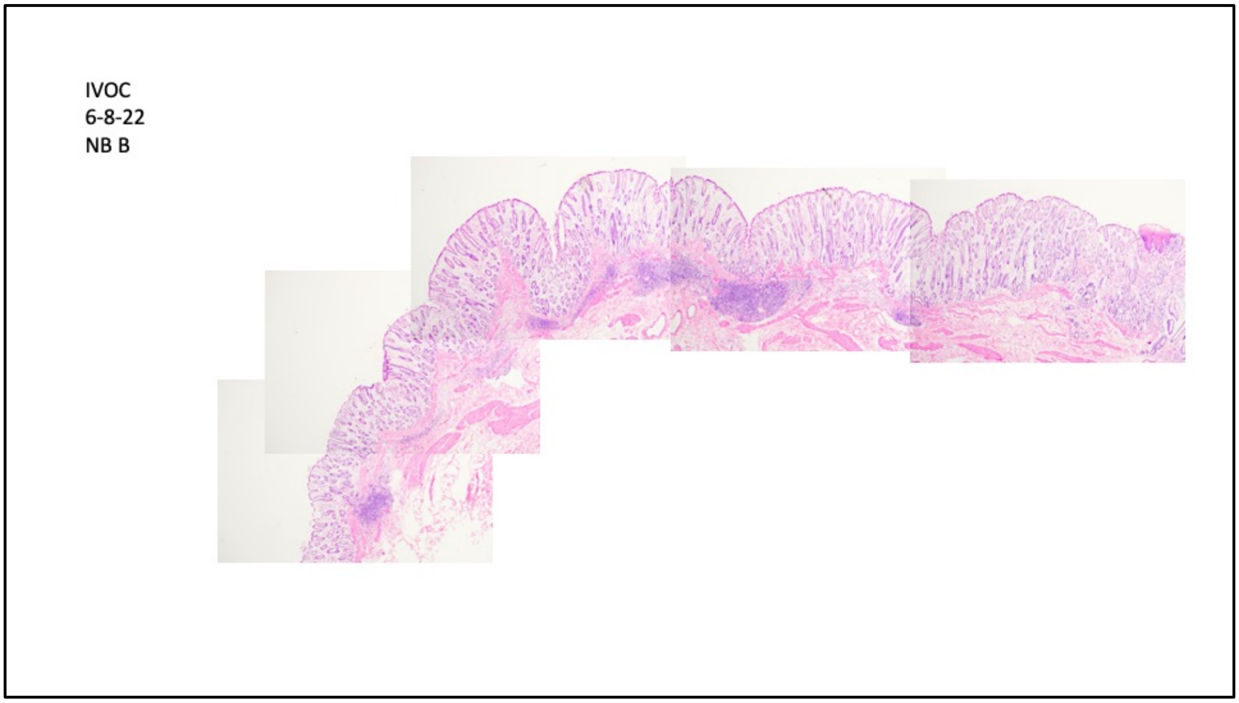

IVOC
6-8-22
NB B
Excellent tissue integrity in all regions.

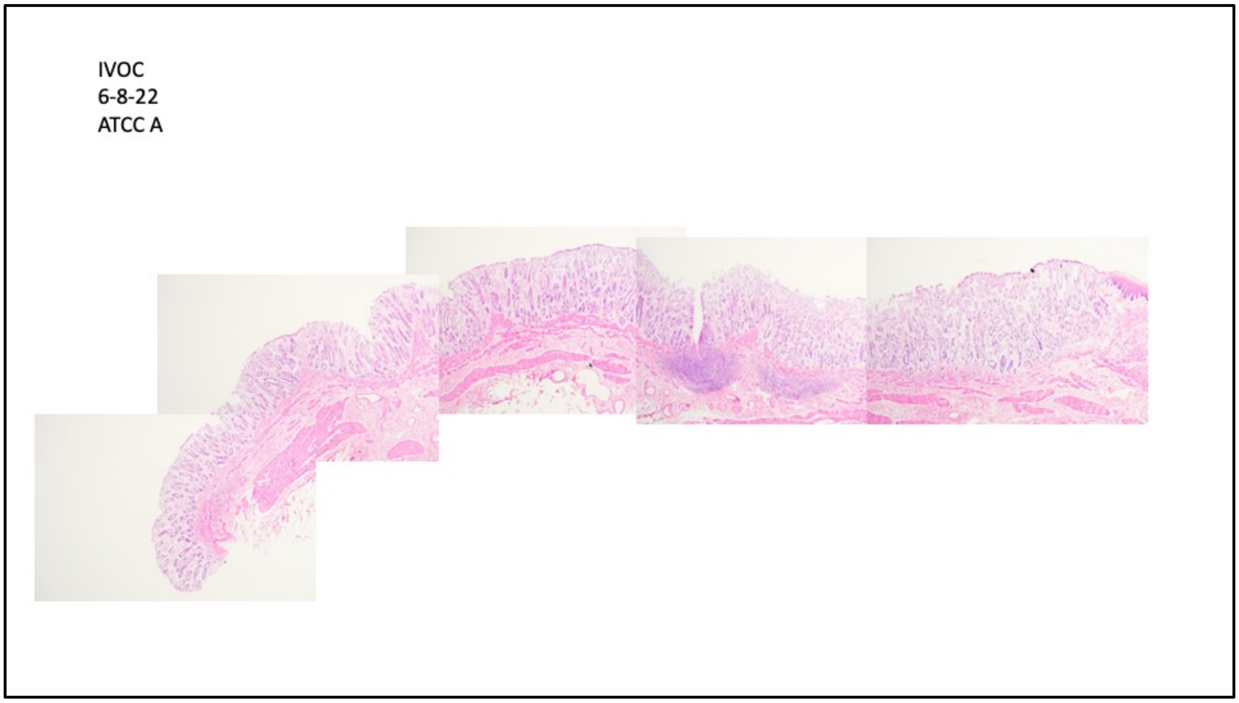

IVOC
6-8-22
ATCC A
>50% of superficial epithelium disrupted in columnar region.
Normal squamous region.

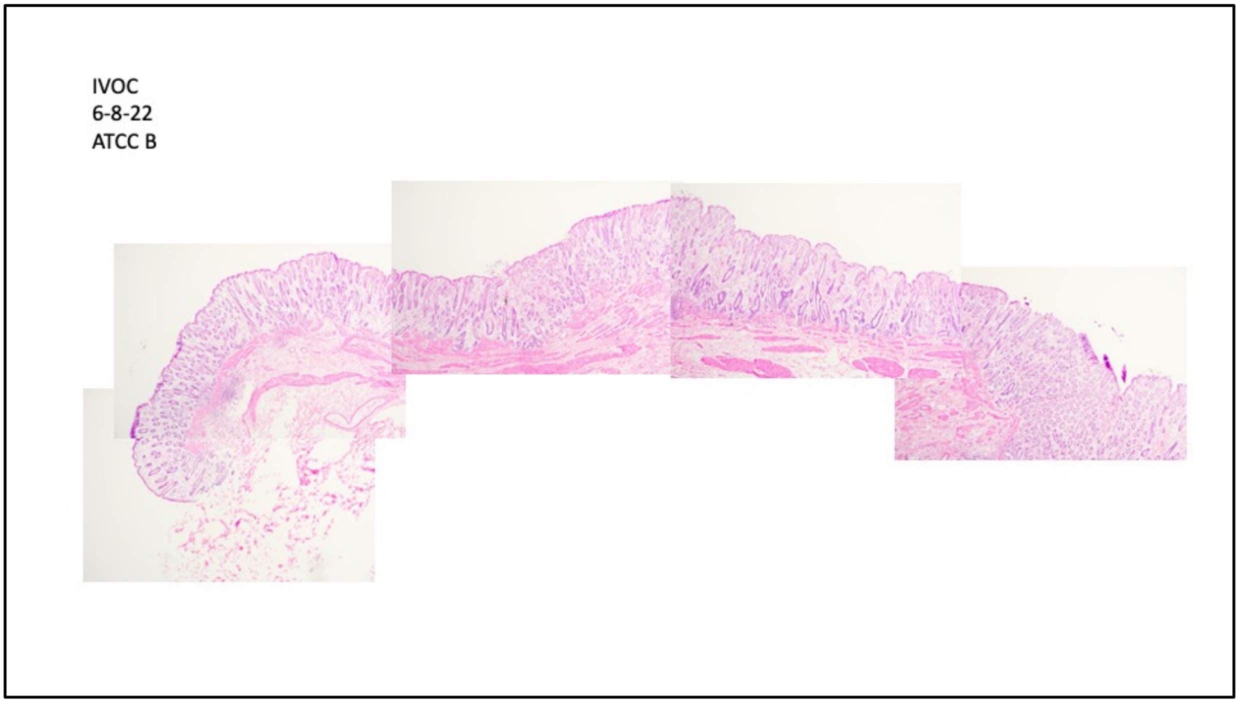

IVOC
6-8-22
ATCC B
Approx 25% of superficial mucosa disrupted in columnar region.
Normal squamous region.

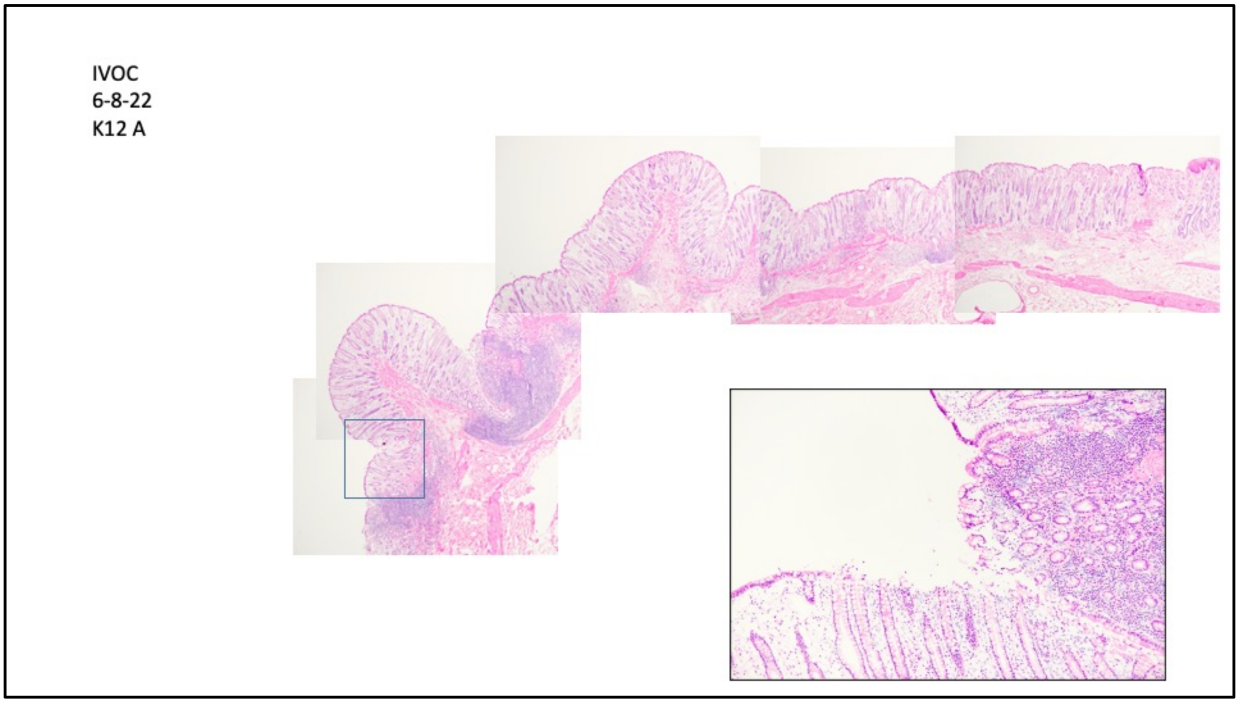

IVOC
6-8-22
K12 A
Focal loss of superficial mucosa.'
Normal squamous region.

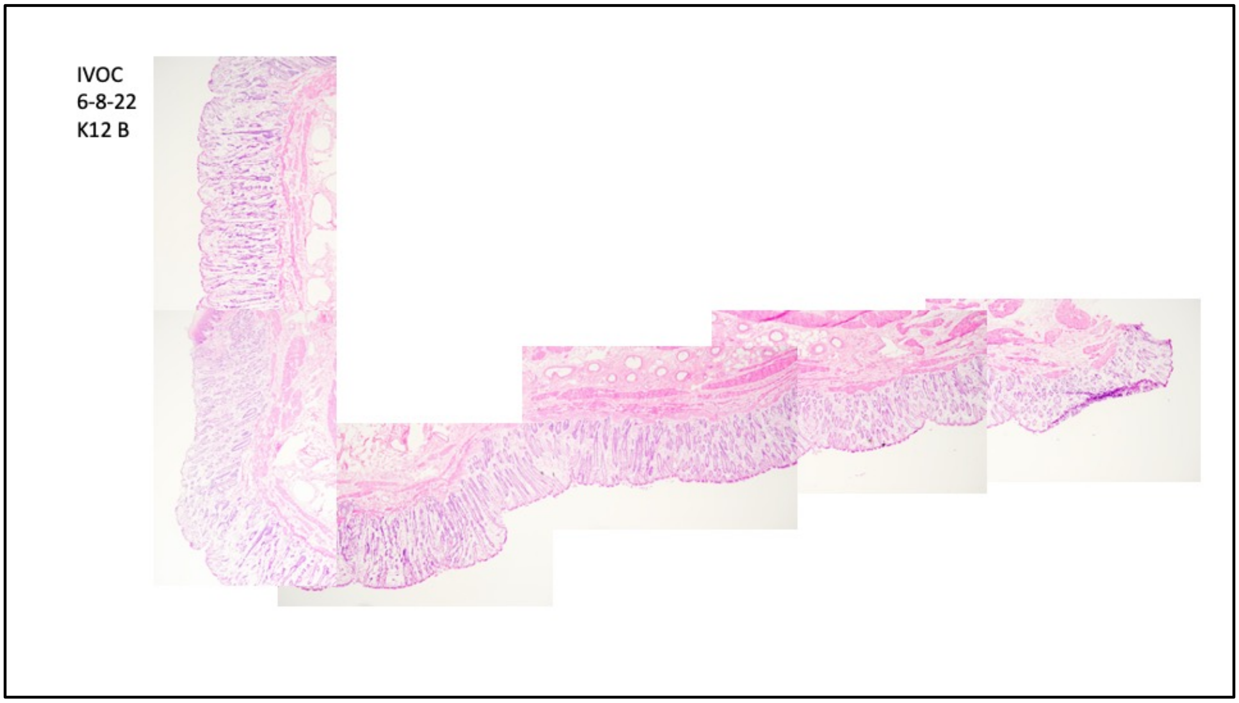

IVOC
6-8-22
K12 B
Excellent tissue integrity in all regions.
