# Supplementary material for: Bovine Rectoanal Junction In Vitro Organ Culture Model System to Study Shiga Toxin-Producing Escherichia coli Adherence
Source: Microorganisms. 2023 May 15;11(5):1289. doi: 10.3390/microorganisms11051289 (PMC10220872; doi:10.3390/microorganisms11051289)
Supplement: Supplementary file 1 [file microorganisms-11-01289-s001.zip › Supp.Data-Histopathology Reports/Supp.Data-HistopathologyReport-S6-Comp10^6-6-15-22_6-29-22.pdf]

Dr. Indira Kudva

Histopathology Results
IVOC Samples
6-15-22
6-29-22

By
Dr. Eric Cassmann
Aug 2022

6/15/22
PRE-ASSAY

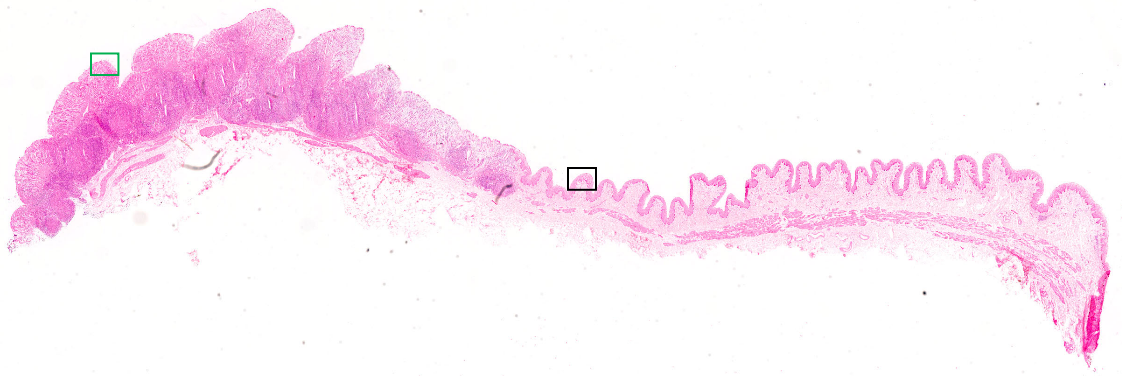

H&E stained frozen section of recto-anal junction approximately 30 mm long that contains squamous epithelium and mucosal columnar epithelial cells with abundant goblet cells. The mucosal portion of tissue contains mucosa, submucosa, circular smooth muscle of the tunica muscularis, and tunica adventitia. Within the mucosa, there are lymphoid nodules – recto-anal mucosal lymphoid tissue (RAMALT). Nuclei of epithelial cells and cells in the RAMALT are stained well. Cells in the lamina propria, submucosa, and tunica adventitia (fibroblasts and other lymphoid mononuclear cells) have pale stained basophilic nuclei.

Black box: In the squamous epithelium there is moderate intercellular bridging (intercellular edema) and intracellular vacuolation of squamous epithelial cells (intracellular edema).

Green box: The surface mucosal epithelium is mostly intact, but there are rare with loss of surface columnar epithelium.

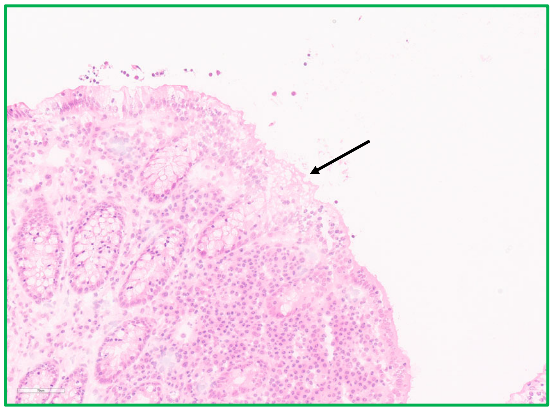

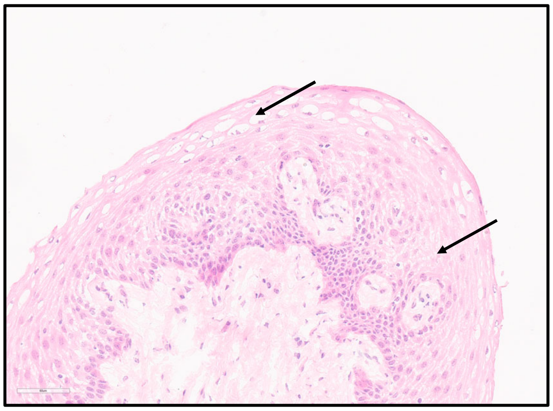

Black box: In the squamous epithelium there is intercellular bridging (intercellular edema) and intracellular vacuolation of squamous epithelial cells (intracellular edema).

Green box: The surface mucosal epithelium is mostly intact, but there are rare with loss of surface columnar epithelium.

6/15/22
NB

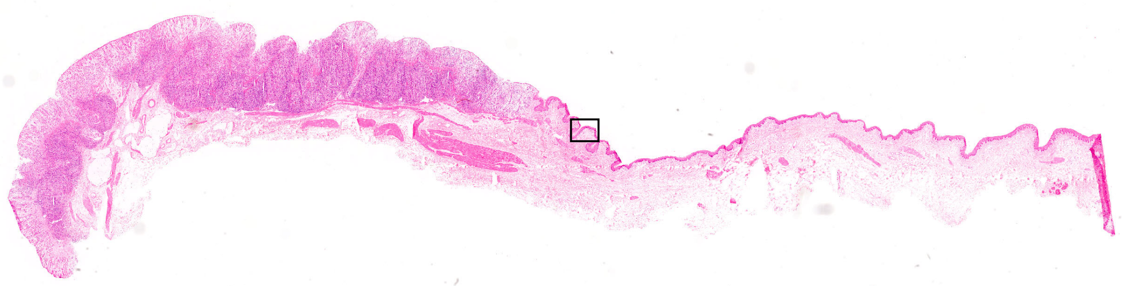

H&E stained frozen section of recto-anal junction approximately 35 mm long that contains squamous epithelium and mucosal columnar epithelial cells with abundant goblet cells. The mucosal portion of tissue contains mucosa, submucosa, circular smooth muscle of the tunica muscularis, and tunica adventitia. Within the mucosa, there are lymphoid nodules – recto-anal mucosal lymphoid tissue (RAMALT). Nuclei of epithelial cells and cells in the RAMALT are stained well. Cells in the lamina propria, submucosa, and tunica adventitia (fibroblasts and other lymphoid mononuclear cells) have well stained basophilic nuclei.

There are multifocal 1.0-1.5 mm disruptions of surface mucosal epithelium. Underlying mucosal and lamina propria is intact.

There is mild to moderate intercellular bridging (intercellular edema) and intracellular vacuolation (intracellular edema/) within the squamous epithelium.

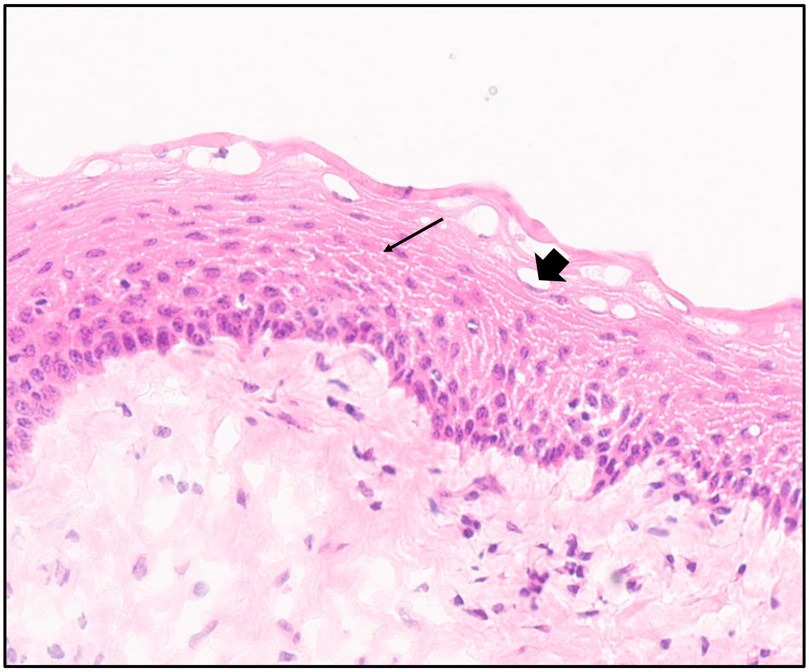

Black arrow: Intercellular bridging (edema)
Black block arrow: Intracellular vacuolation (edema)

6/15/22
EDL933-A

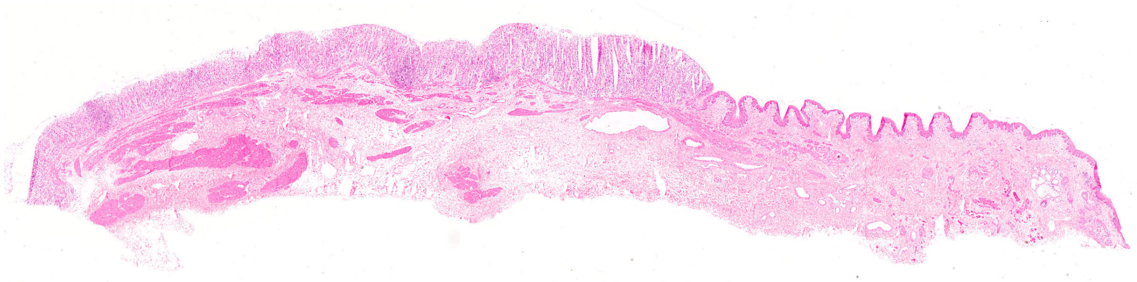

H&E stained frozen section of recto-anal junction approximately 33 mm long that contains squamous epithelium and mucosal columnar epithelial cells with abundant goblet cells. The mucosal portion of tissue contains mucosa, submucosa, circular smooth muscle of the tunica muscularis, and tunica adventitia. Within the mucosa, there are lymphoid nodules – recto-anal mucosal lymphoid tissue (RAMALT). Nuclei of epithelial cells and cells in the RAMALT are stained well. Cells in the lamina propria, submucosa, and tunica adventitia (fibroblasts and other lymphoid mononuclear cells) have well stained basophilic nuclei.

Approximately 33% of the rectal mucosa lacks superficial epithelium. Underlying mucosal and lamina propria is intact.

There is mild intercellular bridging (intercellular edema) and intracellular vacuolation (intracellular edema/) within the squamous epithelium.

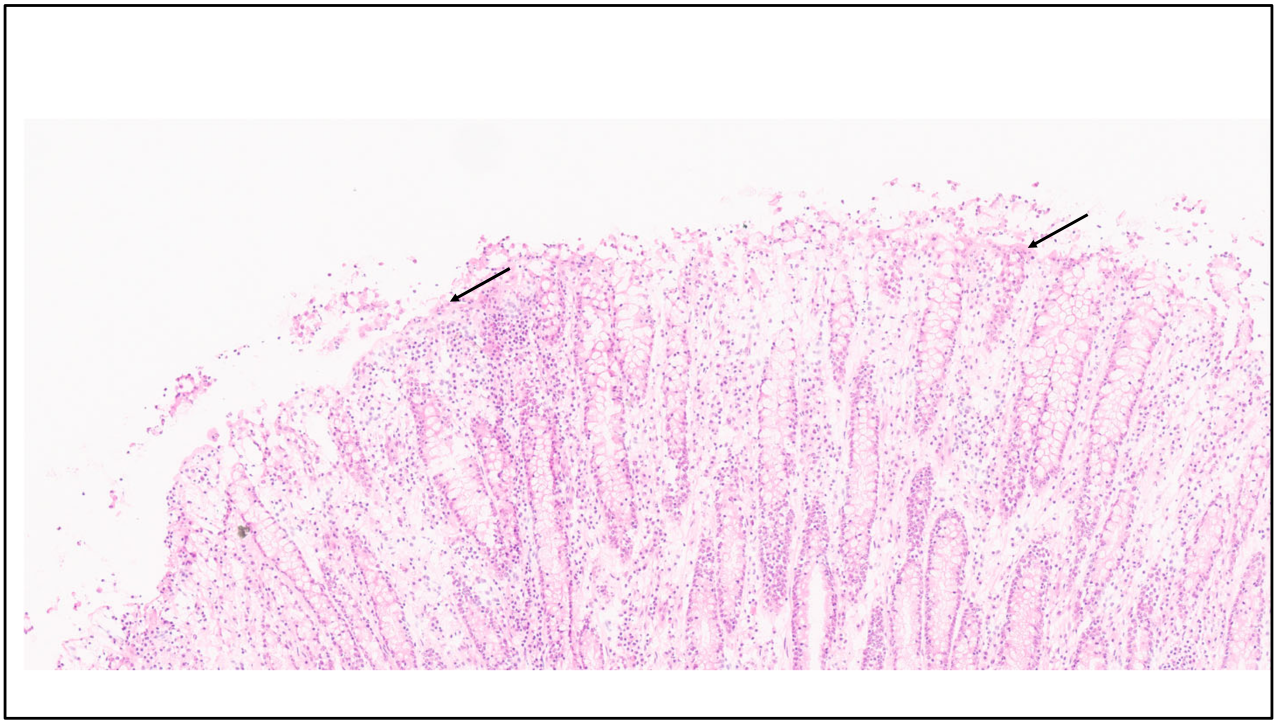

Arrows indicate areas of intact surface epithelium in this filed of view. The rest of the surface mucosal epithelium is disrupted and lost.

6/15/22
EDL933-B

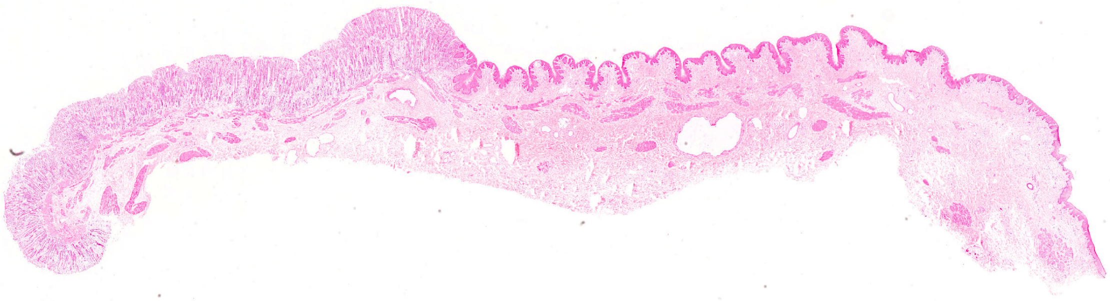

H&E stained frozen section of recto-anal junction approximately 33 mm long that contains squamous epithelium and mucosal columnar epithelial cells with abundant goblet cells. The mucosal portion of tissue contains mucosa, submucosa, circular smooth muscle of the tunica muscularis, and tunica adventitia. Within the mucosa, there are lymphoid nodules – recto-anal mucosal lymphoid tissue (RAMALT). Nuclei of epithelial cells and cells in the RAMALT are stained well. Most cells in the lamina propria, submucosa, and tunica adventitia (fibroblasts and other lymphoid mononuclear cells) have well stained basophilic nuclei.

20-30% of the rectal mucosa lacks superficial epithelium. Underlying mucosal and lamina propria is intact.

There is mild intercellular bridging (intercellular edema) and intracellular vacuolation (intracellular edema/) within the squamous epithelium.

6/15/22
K12

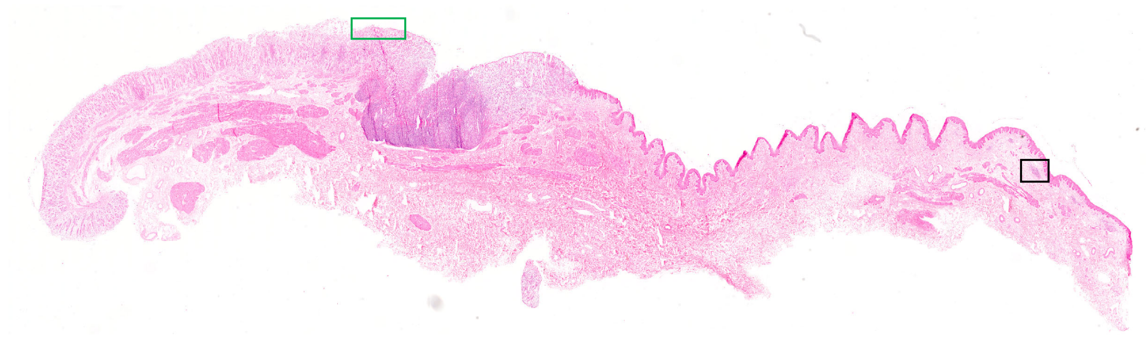

H&E stained frozen section of recto-anal junction approximately 31 mm long that contains squamous epithelium and mucosal columnar epithelial cells with abundant goblet cells. The mucosal portion of tissue contains mucosa, submucosa, circular smooth muscle of the tunica muscularis, and tunica adventitia. Within the mucosa, there are lymphoid nodules – recto-anal mucosal lymphoid tissue (RAMALT). Nuclei of epithelial cells and cells in the RAMALT are stained well. Most cells in the lamina propria, submucosa, and tunica adventitia (fibroblasts and other lymphoid mononuclear cells) have well stained basophilic nuclei.

35-40% of the rectal mucosa lacks superficial epithelium. Underlying mucosal and lamina propria is intact.

There is overall minimal to mild intercellular bridging (intercellular edema) and intracellular vacuolation (intracellular edema/) within the squamous epithelium; however, there is a single foci of prominent intracellular vacuolation within squamous epithelium

There are multifocal perivascular lymphoid aggregates in the dermis of the squamous epithelial component of the RAJ.

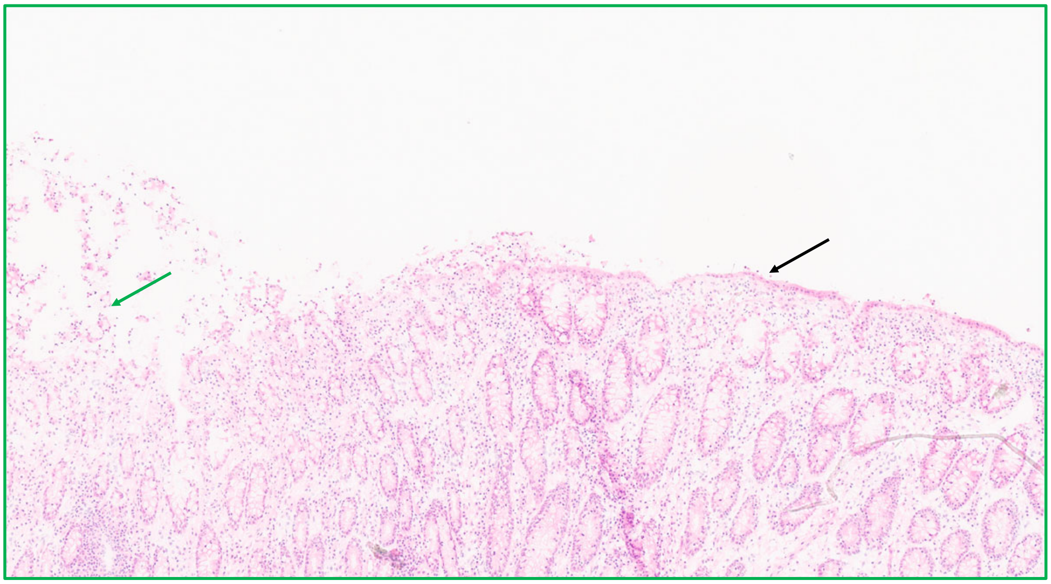

Green Arrow: absent/ disrupted superficial mucosal epithelium
Black Arrow: intact superficial mucosal epithelium

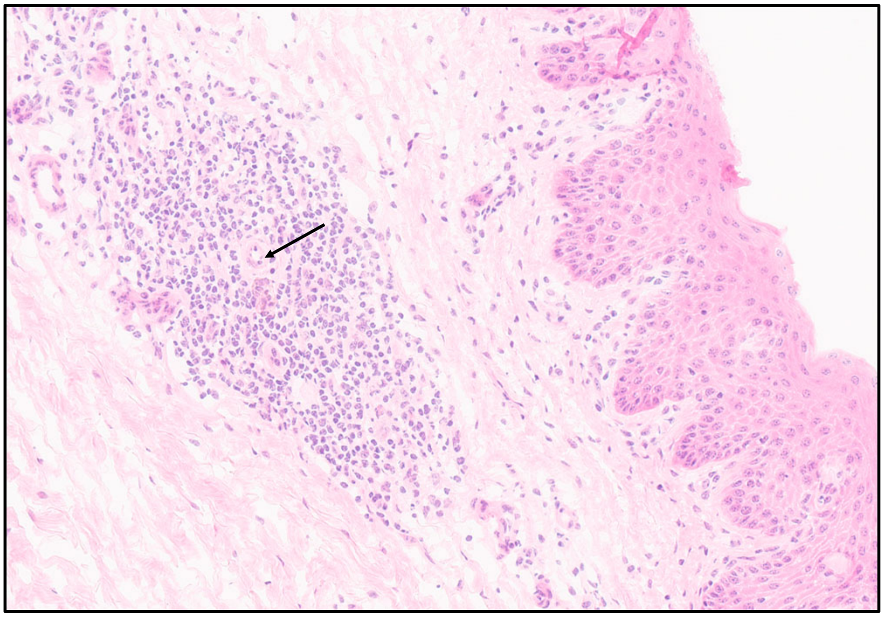

Aggregates of lymphocytes around a small arteriole in the dermis of the squamous portion of the recto-anal tissue.

The perivascular lymphocytic infiltrate accumulated antemortem and not during IVOC

6/15/22
Summary

A recurring finding in IVOC test samples was the loss of superficial surface mucosal epithelium. This finding was rare in the Pre-Assay sample indicating that test conditions (bacterial colonization) may influence the adherence of superficial epithelium in the IVOC model. In areas with loss of superficial mucosal epithelium, the underlying and subjacent lamina propria lacked inflammatory infiltrates. Therefore, the disruption of surface epithelium was post-mortem and is thought to be due to post-mortem mechanical disruption possibly in combination with test conditions (bacterial colonization). Overall, the degree of tissue loss was minimal, and more than 70-90% of surface mucosa was present in most sections.

Other recurring findings were intracellular and intercellular edema within the squamous epithelium. These findings are most likely post-mortem artifacts related to tissue processing methods and/or IVOC procedures since the finding is present within Pre-assay and test IVOC samples alike.

6/29/22
PRE-ASSAY

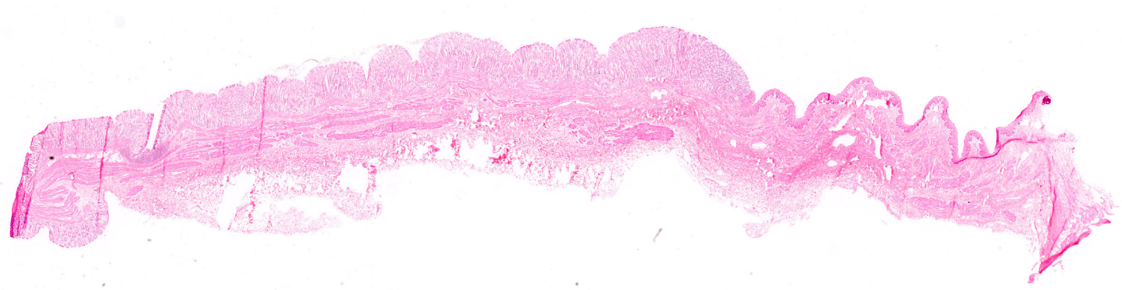

H&E-stained frozen section of recto-anal junction approximately 32 mm long that contains squamous epithelium and mucosal columnar epithelial cells with abundant goblet cells. The mucosal portion of tissue contains mucosa, submucosa, circular smooth muscle of the tunica muscularis, and tunica adventitia. Within the mucosa, there is a lymphoid nodule – recto-anal mucosal lymphoid tissue (RAMALT). Nuclei of epithelial cells and cells in the RAMALT are stained well. Cells in the lamina propria, submucosa, and tunica adventitia (fibroblasts and other lymphoid mononuclear cells) have well stained basophilic nuclei.

The mucosal surface epithelium is well intact in this section.

In the squamous epithelium there is diffuse mild intercellular bridging (intercellular edema) and moderate intracellular vacuolation of squamous epithelial cells (intracellular edema).

There is multifocal artifactual separation of the dermis from the epidermis.

6/29/22
NB

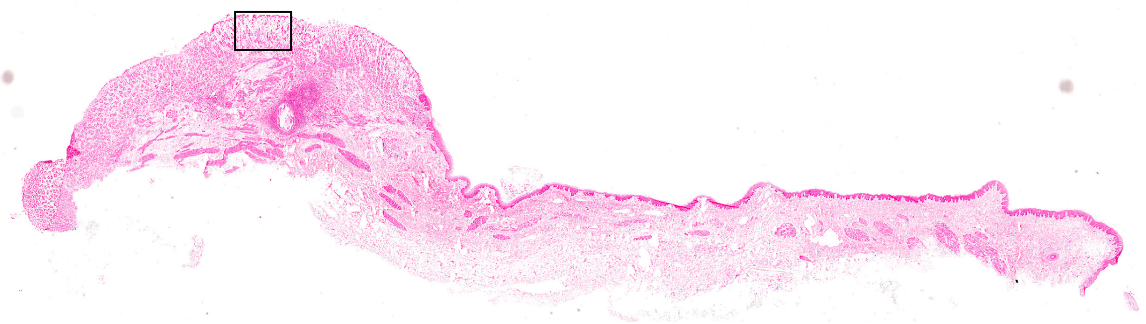

H&E-stained frozen section of recto-anal junction approximately 29 mm long that contains squamous epithelium and mucosal columnar epithelial cells with abundant goblet cells. The mucosal portion of tissue contains mucosa, submucosa, circular smooth muscle of the tunica muscularis, and tunica adventitia. Subjacent to the mucosa, there is a single lymphoid nodule (with herniated crypt) – recto-anal mucosal lymphoid tissue (RAMALT). Nuclei of epithelial cells and cells in the RAMALT are stained well. Cells in the lamina propria, submucosa, and tunica adventitia (fibroblasts and other lymphoid mononuclear cells) have pale stained basophilic nuclei. There is increased separation between glands and epithelial cells in the mucosal (loss of glandular component).

40-50% of the rectal mucosa lacks superficial epithelium. The underlying mucosal and lamina propria is intact.

In the squamous epithelium there is multifocal mild to moderate intercellular bridging (intercellular edema) and intracellular vacuolation of squamous epithelial cells (intracellular edema).

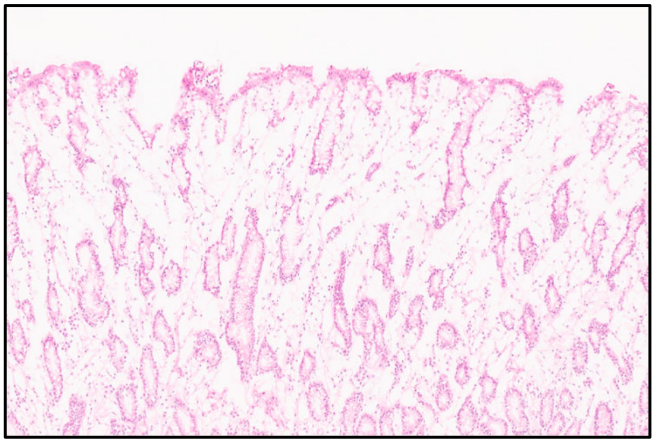

There is increased white space between epithelial cells and goblet cells that form the crypts. This is concomitant with loss of epithelial component.

6/29/22
EDL933-A

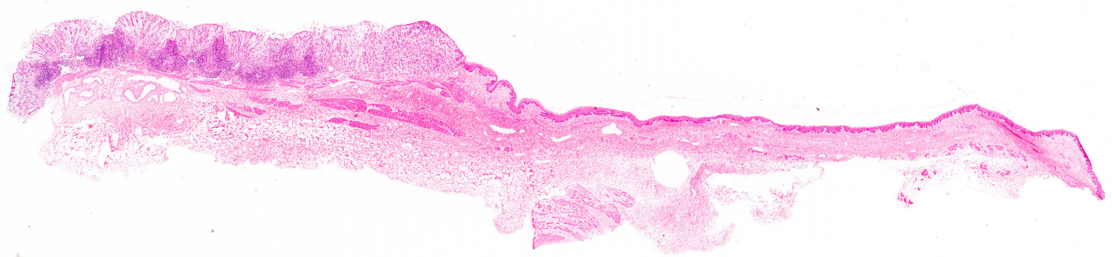

H&E-stained frozen section of recto-anal junction approximately 33 mm long that contains squamous epithelium and mucosal columnar epithelial cells with abundant goblet cells. The mucosal portion of tissue contains mucosa, submucosa, circular smooth muscle of the tunica muscularis, and tunica adventitia. In the mucosa, there are lymphoid nodules – recto-anal mucosal lymphoid tissue (RAMALT). The lymphoid nodules are hyperplastic in this section with prominent germinal centers. Nuclei of epithelial cells and cells in the RAMALT are stained well. Cells in the lamina propria, submucosa, and tunica adventitia (fibroblasts and other lymphoid mononuclear cells) have pale stained basophilic nuclei.

15-25% of the rectal mucosa lacks superficial epithelium. The underlying mucosal and lamina propria is intact.

In the squamous epithelium there is minimal intercellular bridging (intercellular edema) and mild intracellular vacuolation of squamous epithelial cells (intracellular edema).

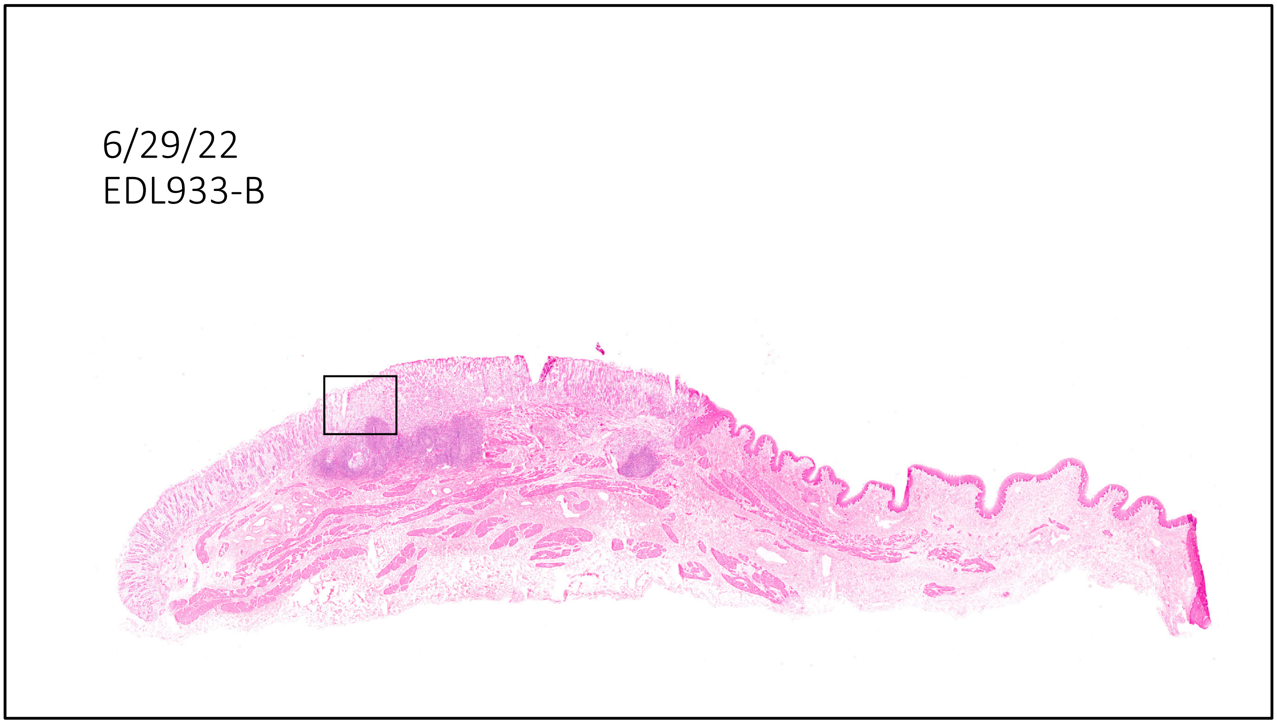

H&E-stained frozen section of recto-anal junction approximately 26 mm long that contains squamous epithelium and mucosal columnar epithelial cells with abundant goblet cells. The mucosal portion of tissue contains mucosa, submucosa, circular smooth muscle of the tunica muscularis, and tunica adventitia. Subjacent to the mucosa, there is a single lymphoid nodule (with herniated crypt) – recto-anal mucosal lymphoid tissue (RAMALT). Nuclei of epithelial cells and cells in the RAMALT are stained well. Cells in the lamina propria, submucosa, and tunica adventitia (fibroblasts and other lymphoid mononuclear cells) have well stained basophilic nuclei.

20-30% of the rectal mucosa lacks superficial epithelium. The underlying mucosal and lamina propria is mostly intact but disrupted in rare foci.

In the squamous epithelium there is focal mild to moderate intercellular bridging (intercellular edema) and multifocal mild to moderate intracellular vacuolation of squamous epithelial cells (intracellular edema).

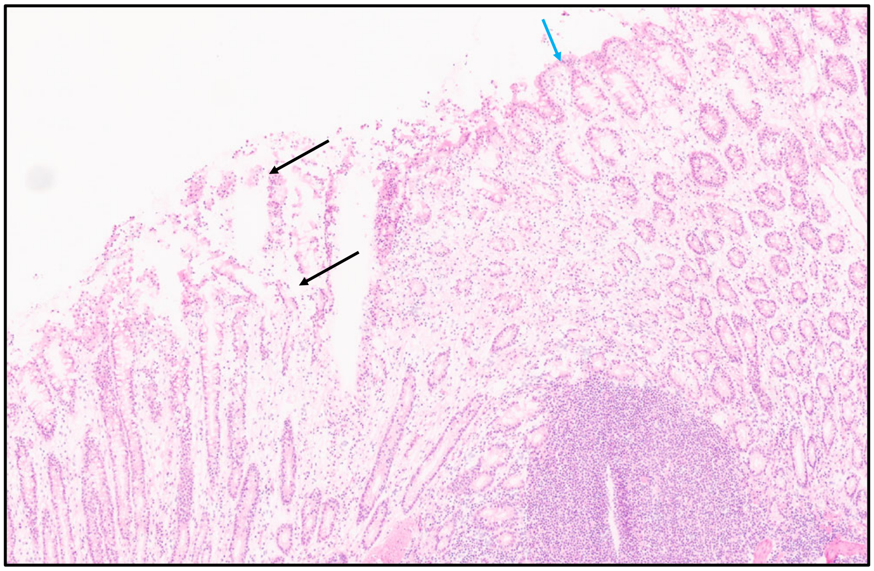

The overlying superficial mucosal epithelium and underlying crypt epithelium within the lamina propria are disrupted (black arrows). Most of the superficial mucosal epithelium is intact throughout the entire section (short blue arrow).

6/29/22
K12

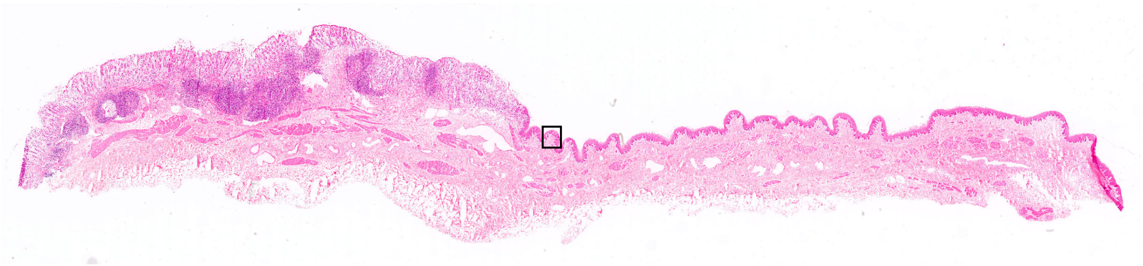

H&E-stained frozen section of recto-anal junction approximately 34 mm long that contains squamous epithelium and mucosal columnar epithelial cells with abundant goblet cells. The mucosal portion of tissue contains mucosa, submucosa, circular smooth muscle of the tunica muscularis, and tunica adventitia. Subjacent to the mucosa, there is a single lymphoid nodule (with herniated crypt) – recto-anal mucosal lymphoid tissue (RAMALT). Nuclei of epithelial cells and cells in the RAMALT are stained well. Cells in the lamina propria, submucosa, and tunica adventitia (fibroblasts and other lymphoid mononuclear cells) have well stained basophilic nuclei.

30-40% of the rectal mucosa lacks superficial epithelium. The underlying mucosal and lamina propria is mostly intact.

In the squamous epithelium there is multifocal mild to moderate intercellular bridging (intercellular edema) and multifocal mild to moderate intracellular vacuolation of squamous epithelial cells (intracellular edema). There is some tissue folding that does no interfere with interpretation.

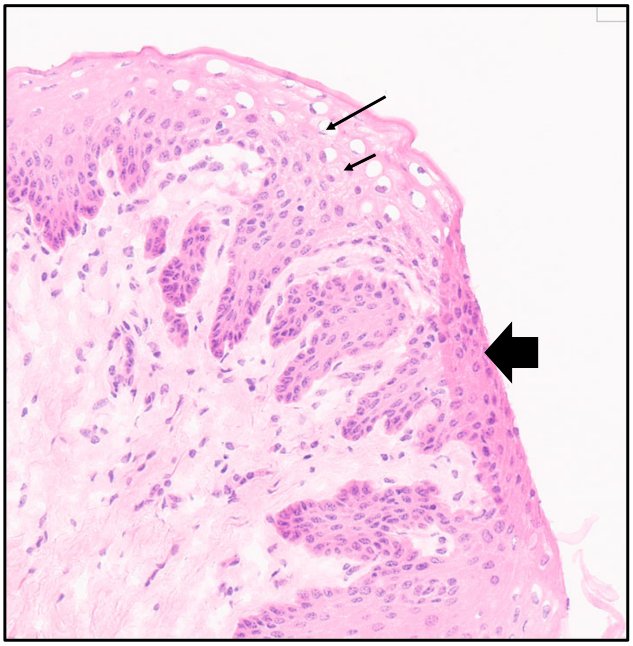

In the epidermis there is intracellular vacuolation (long arrow) and prominent intercellular bridging (short arrow). There is limited /mild folding of the tissue (block arrow) that does not limit interpretation.

6/29/22
Summary

Overall, the findings are like the 6/15/22 and 6/22/22 timepoints.

A recurring finding in IVOC test samples was the loss of superficial surface mucosal epithelium. This finding was rare in the Pre-Assay sample indicating that test conditions (bacterial colonization) and/or elapsed time may influence the adherence of superficial epithelium in the IVOC model. In areas with loss of superficial mucosal epithelium, the underlying and subjacent lamina propria lacked pathologic changes that would be expected with antemortem ulcerations (i.e neutrophils and fibrin). Therefore, the disruption of surface epithelium occurred post-mortem and is thought to be due to post-mortem mechanical disruption possibly in combination with test conditions (bacterial colonization).

There was a noteworthy difference between NB and Pre-Assay. The superficial mucosal epithelium remained well intact in the Pre-Assay, so the elapsed time between Pre-assay and the NB negative control may influence surface mucosal integrity. Overall, the degree of tissue loss varied throughout this timepoint; the range of surface mucosal epithelium present in test tissue was 25-85% (15-75% absent).

Other recurring findings were intracellular and intercellular edema within the squamous epithelium. These findings are most likely post-mortem artifacts related to tissue processing methods and/or IVOC procedures since the finding is present within Pre-assay and test IVOC samples alike.
