# Supplementary material for: Bovine Rectoanal Junction In Vitro Organ Culture Model System to Study Shiga Toxin-Producing Escherichia coli Adherence
Source: Microorganisms. 2023 May 15;11(5):1289. doi: 10.3390/microorganisms11051289 (PMC10220872; doi:10.3390/microorganisms11051289)
Supplement: Supplementary file 1 [file microorganisms-11-01289-s001.zip › SupplementaryFigures.pdf]

Research-Supp. Figures.

Bovine Rectoanal Junction-In vitro Organ Culture Model System to Study Shiga Toxin-Producing *Escherichia coli* Adherence.

Indira T. Kudva[1,*], Erika N. Biernbaum[1,2], Eric D. Cassmann[3], and Mitchell V. Palmer[4]

SUPPLEMENTARY FIGURES

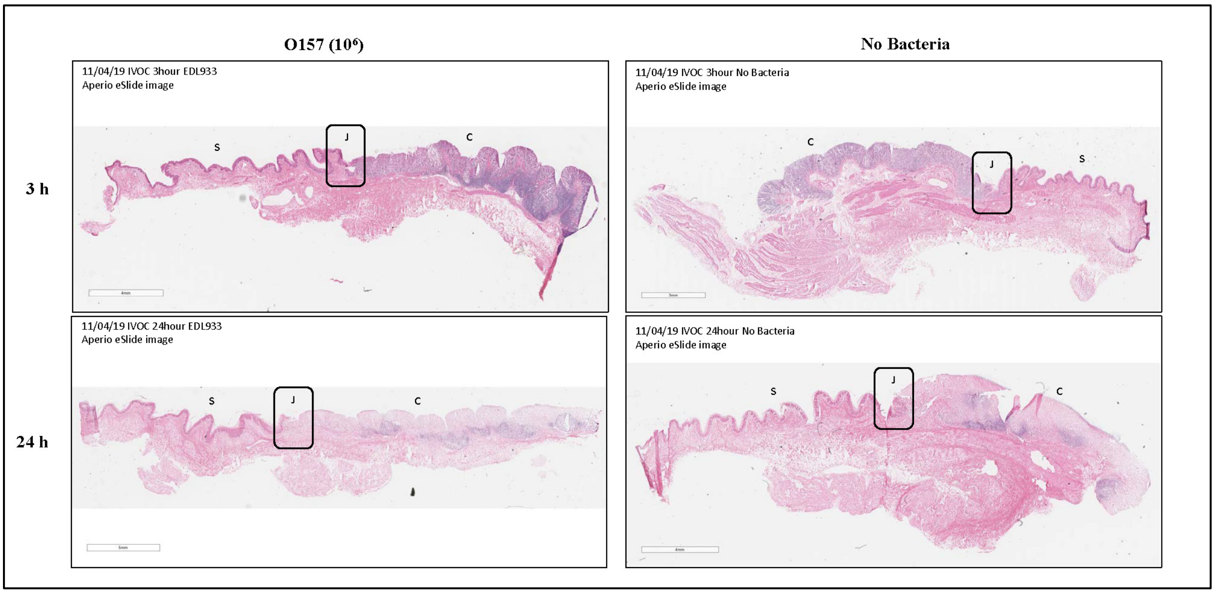

Figure S1. eImages of RAJ-IVOC tissue sections from Trial 1. The RAJ-IVOC was inoculated with either O157 (10^6 CFU inoculum as shown in parenthesis), or not inoculated (no bacteria) and incubated at 39°C for 3 or 24 h. H&E-stained tissue section slides were scanned using the Aperio digital pathology system to obtain the eImages. The squamous (S), junction (J, boxed), and columnar (C) regions of the RAJ are indicated. A 3 mm scale bar is shown.

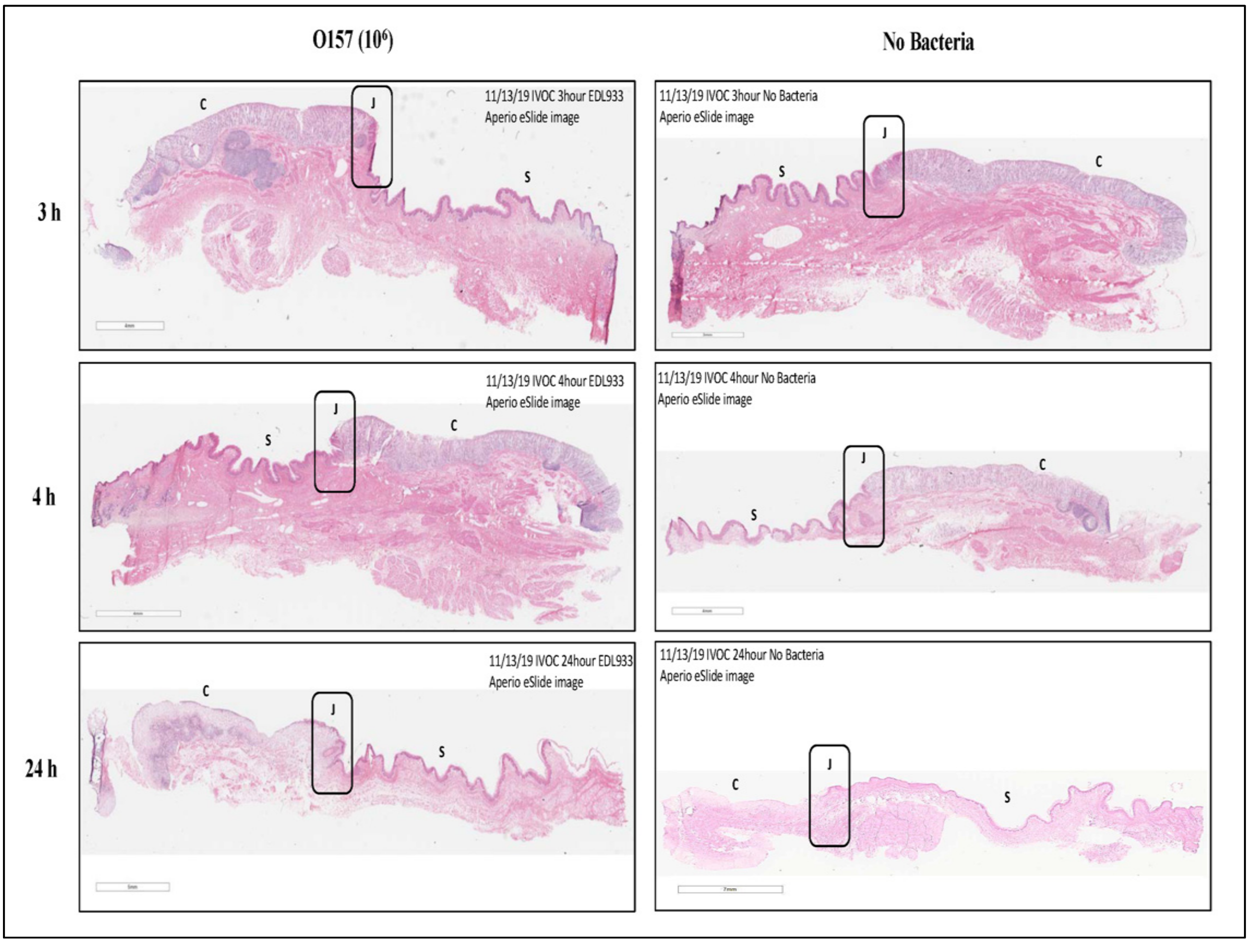

Figure S2. eImages of RAJ-IVOC tissue sections from Trial 2. The RAJ-IVOC was inoculated with either O157 (10^6 CFU inoculum as shown in parenthesis), or not inoculated (no bacteria) and incubated at 39°C for 3, 4 or 24 h. H&E-stained tissue section slides were scanned using the Aperio digital pathology system to obtain the eImages. The squamous (S), junction (J, boxed), and columnar (C) regions of the RAJ are indicated. A 3 mm scale bar is shown.

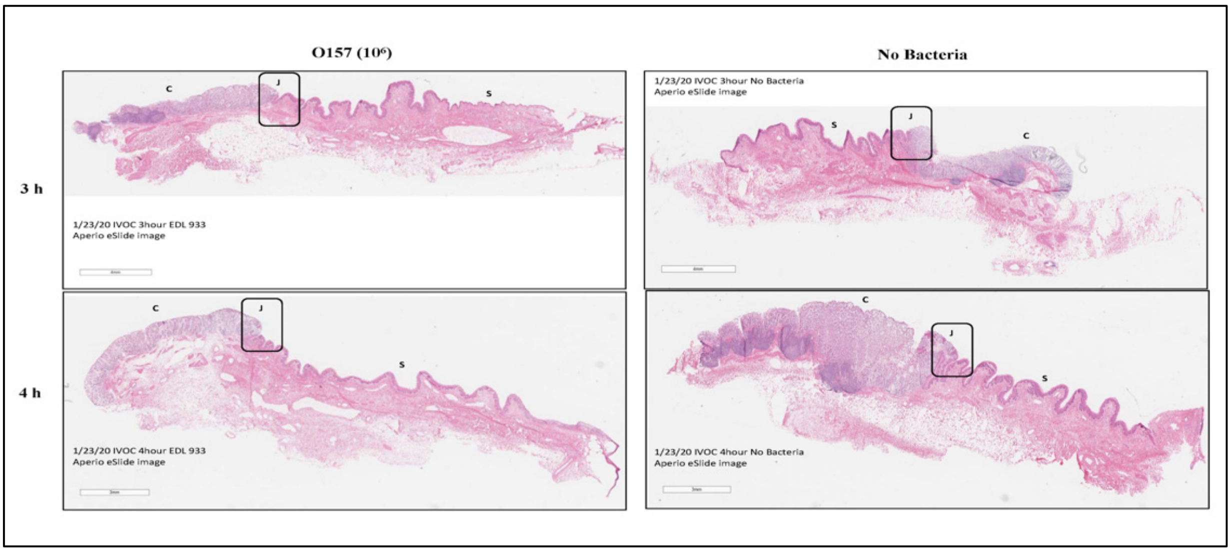

Figure S3. eImages of RAJ-IVOC tissue sections from Trial 3. The RAJ-IVOC was inoculated with either O157 (10^6 CFU inoculum as shown in parenthesis), or not inoculated (no bacteria) and incubated at 39°C for 3 or 4 h. H&E-stained tissue section slides were scanned using the Aperio digital pathology system to obtain the eImages. The squamous (S), junction (J, boxed), and columnar (C) regions of the RAJ are indicated. A 3 mm scale bar is shown.

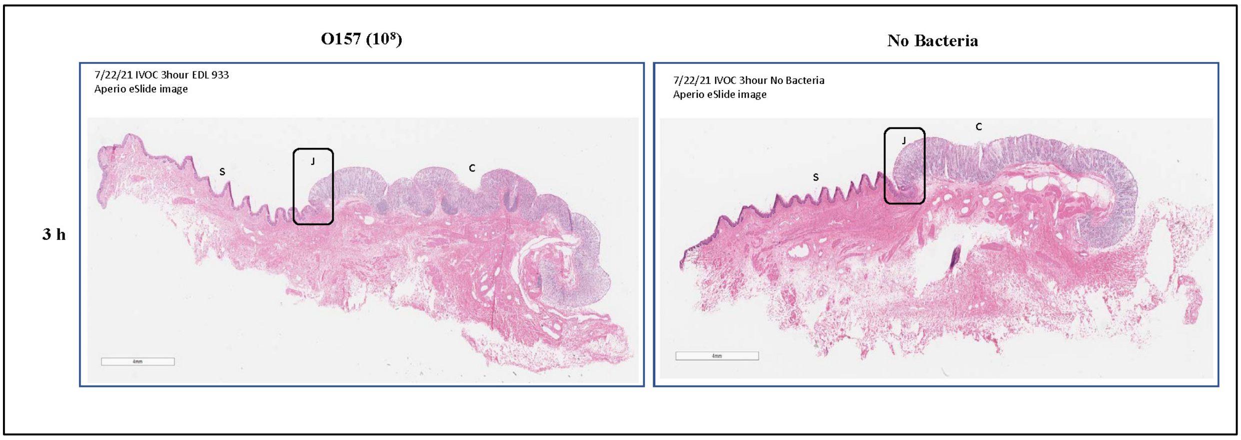

Figure S4. eImages of RAJ-IVOC tissue sections from Trial 4. The RAJ-IVOC was inoculated with either O157 (10^8 CFU inoculum as shown in parenthesis), or not inoculated (no bacteria) and incubated at 39°C for 3 h. H&E-stained tissue section slides were scanned using the Aperio digital pathology system to obtain the eImages. The squamous (S), junction (J, boxed), and columnar (C) regions of the RAJ are indicated. A 3 mm scale bar is shown.

1.

2.

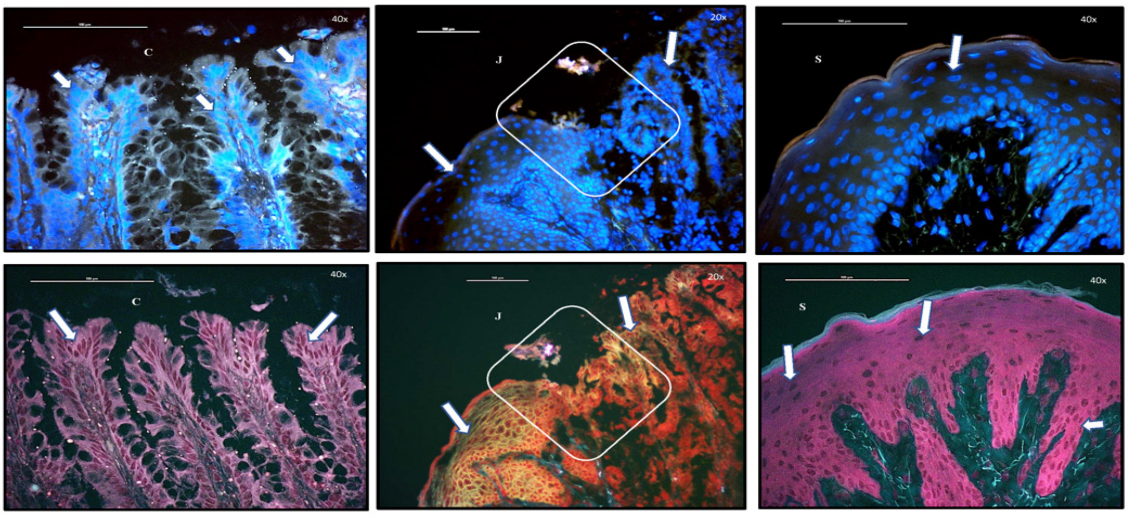

Figure S5. Images of a fixed-RAJ-IVOC tissue stained with DAPI (Panel 1) and REdDot2 (Panel 2). The nuclei in the DAPI-stained RAJ-IVOC tissue sections have a blue florescence while RedDot2-stained nuclei are red, as shown with arrows. The squamous (S), junction (J, boxed), and columnar (C) regions of the RAJ are shown along with the 100 µm scale bar. Images were captured at 200x or 400x magnification; the objective used is indicated on the images.

A.

No bacteria | **O157 strain EDL 933** | ***E. coli* K12**

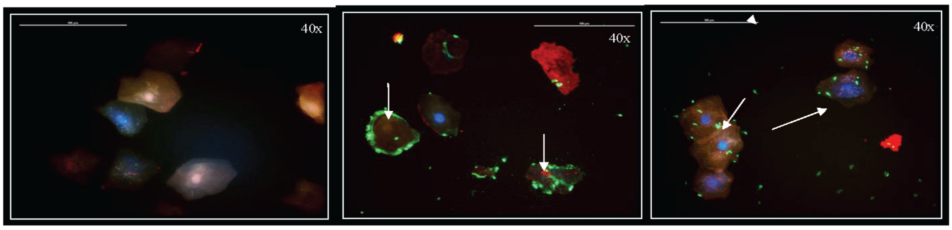

B.

Quantitation of RSE cells with adherent test bacteria

Bacteria tested	Bacterial adherence pattern	RSE cells (n=160)[a] with adherent bacteria, in the ranges shown, for three different trials[b] (MOI[(c)] = 10^6 bacteria: 10^5 cells)						Percent mean ± standard error of mean of RSE cells with adherent bacteria in the ranges shown[e]	
		Assay I		Assay II		Assay III			
		>10	1-10[d]	>10	1-10	>10	1-10	>10	1-10
O157 str. EDL933 (ATCC 43895)	**Aggregative, moderate**	20	130	37	123	21	134	16.25 ± 3.442	**80.63 ± 2.009**
E. coli K12 (MG1655)	**Diffuse, moderate**	0	87	7	116	0	115	1.458 ± 1.458	**66.25 ± 5.940**

[a] Total number of cells evaluated in each trial.
[b] Each trial had one slide per bacterial group. Each slide in turn had 8 technical replicates spotted on it; 10-20 well-dispersed cells were evaluated per spot to get a total of n=160.
[c] MOI, multiplicity of infection.
[d] Number of bacteria adhering to each cell is shown as a range of >10 and 1-10. Number of cells without bacteria is not shown.
[e] Percent means for ranges used to determine "moderate" adherence are in bold.

Figure S6. Qualitative (Panel A) and quantitative (Panel B) data from the RSE cell adherence assay. The 'aggregative, moderate' and 'diffuse, moderate' adherence patterns of the O157 and E. coli K12 bacteria on RSE cells are shown in Panel A. The immunofluorescent images were captured at 400x magnification; the

objective used is shown on the images along with the 100 μm scale bar. The bacteria (O157 and E. coli K12) as indicated by arrows, the RSE cells' cytokeratins, and the nuclei have green, orange-red and blue fluorescence, respectively. The quantitative data from three adherence assays are shown in the table in Panel B; a statistically significant difference in quantitative adherence ($p<0.014$) was observed.

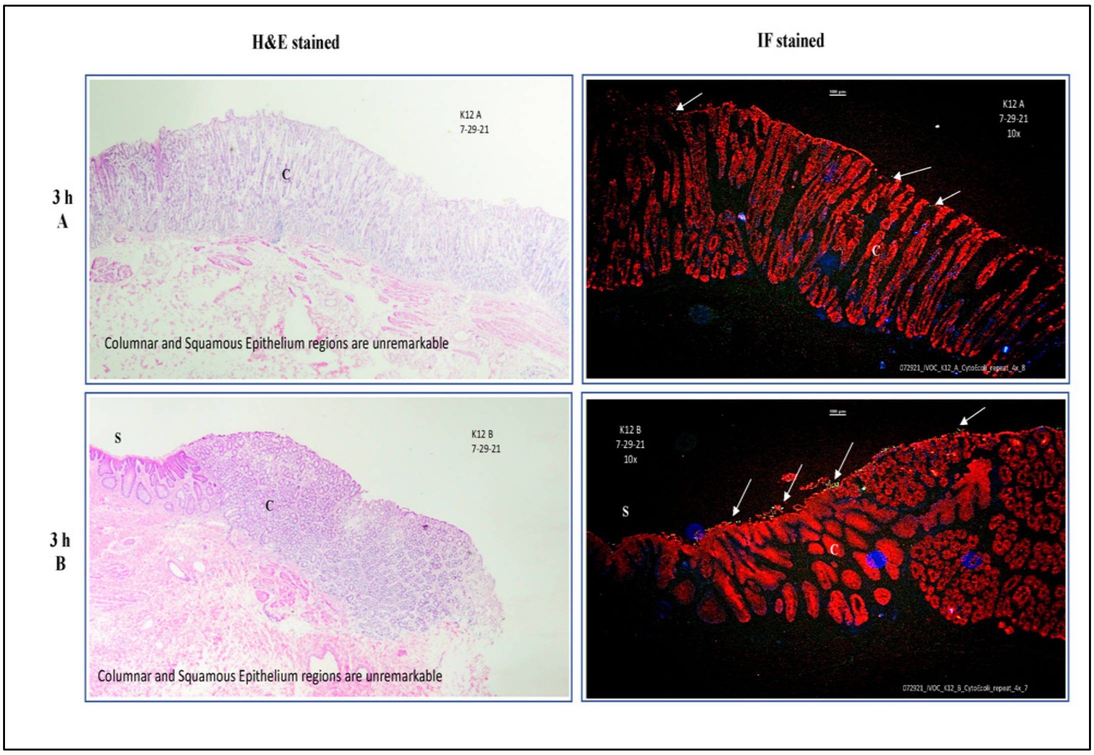

Figure S7. H&E-stained eImages, and immunofluorescent (IF) images of tissue sections from two RAJ-IVOC pilot trials, A and B, with E. coli K12. The RAJ-IVOCs were inoculated with 10^8 CFU E. coli K12 and incubated at 39°C for 3 h. The H&E-stained tissue section slides were scanned using the Aperio digital pathology system to obtain the eImages. The immunofluorescent images were recorded at 100x magnification; the objective used is shown on the image. The bacteria (E. coli K12) as indicated by arrows, the RSE cells' cytokeratins, and the nuclei have green, orange-red and blue fluorescence, respectively. The squamous (S), junction (J), and columnar (C) regions of the RAJ are indicated.

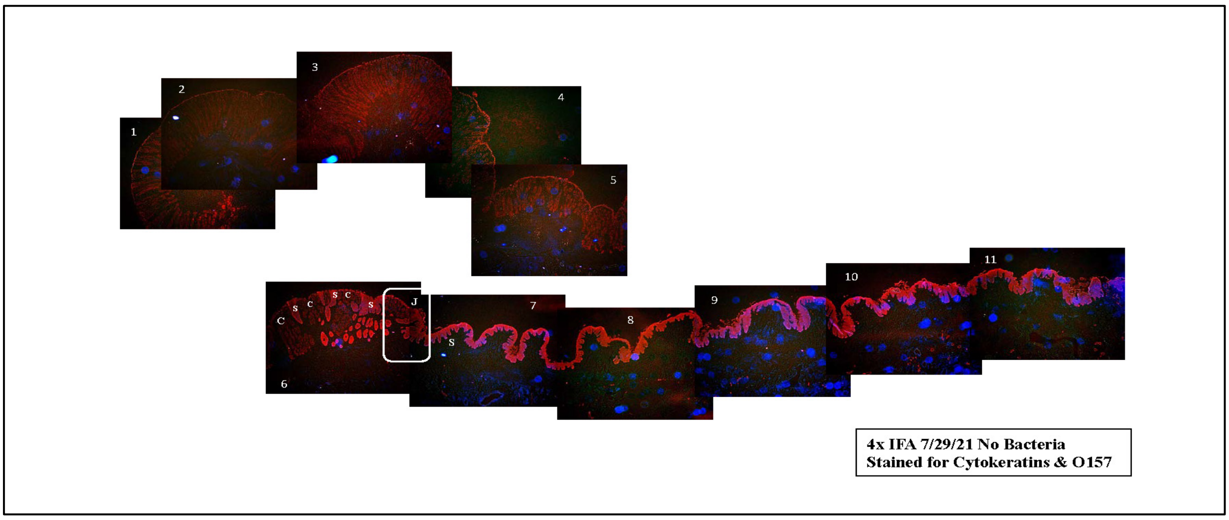

Figure S8. Stitched immunofluorescent images of a tissue section from a representative 'no bacteria-RAJ-IVOC' control used in the comparative adherence assay. The tissue section was stained with antibodies targeting the RAJ cells and O157 to demonstrate the absence of O157 in the control. The immunofluorescent images were recorded at 40x magnification; the objective used is shown on the image-label. The images were stitched in the order shown, panels 1-11, to provide a complete picture of the RAJ. The RAJ cells' cytokeratins, and the nuclei have orange-red and blue fluorescence, respectively. The squamous (S), junction (J), and columnar (C) regions of the RAJ are indicated.

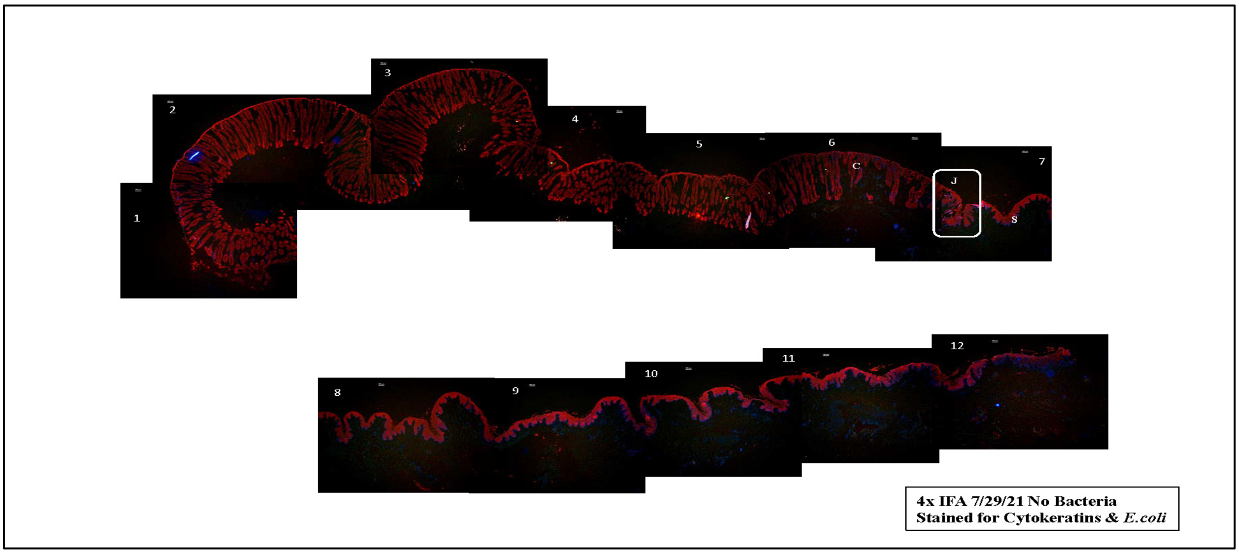

Figure S9. Stitched immunofluorescent images of a tissue section from a representative 'no bacteria-RAJ-IVOC' control used in the comparative adherence assay. The tissue section was stained with antibodies targeting the RAJ cells and E. coli to demonstrate the absence of E. coli in the control. The immunofluorescent images were recorded at 40x magnification; the objective used is shown on the image-label. The images were stitched in the order shown, panels 1-12, to provide a complete picture of the RAJ. The RAJ cells' cytokeratins, and the nuclei have orange-red and blue fluorescence, respectively. The squamous (S), junction (J, boxed), and columnar (C) regions of the RAJ are indicated.

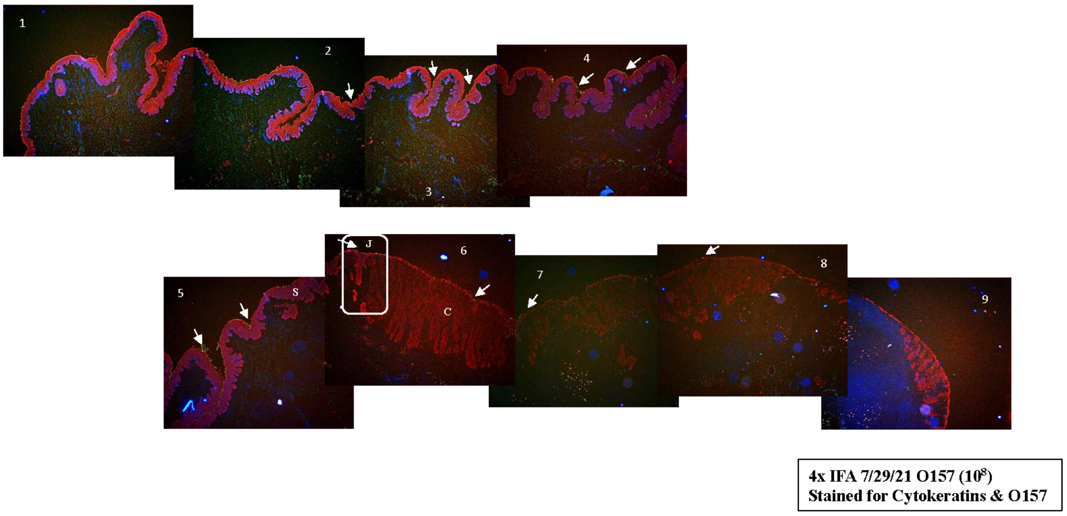

Figure S10. Stitched immunofluorescent images of a tissue section from a representative RAJ-IVOC inoculated with 10^8 CFU O157 in the comparative adherence assay. The tissue section was stained with antibodies targeting the RAJ cells and O157 to demonstrate the presence of adherent O157. The immunofluorescent images were recorded at 40x magnification; the objective used is shown on the image-label. The images were stitched in the order shown, panels 1-9, to provide a complete picture of the RAJ with adherent O157 indicated with arrows. The adherent O157, RAJ cells' cytokeratins, and the nuclei have green, orange-red and blue fluorescence, respectively. The squamous (S), junction (J, boxed), and columnar (C) regions of the RAJ are indicated.

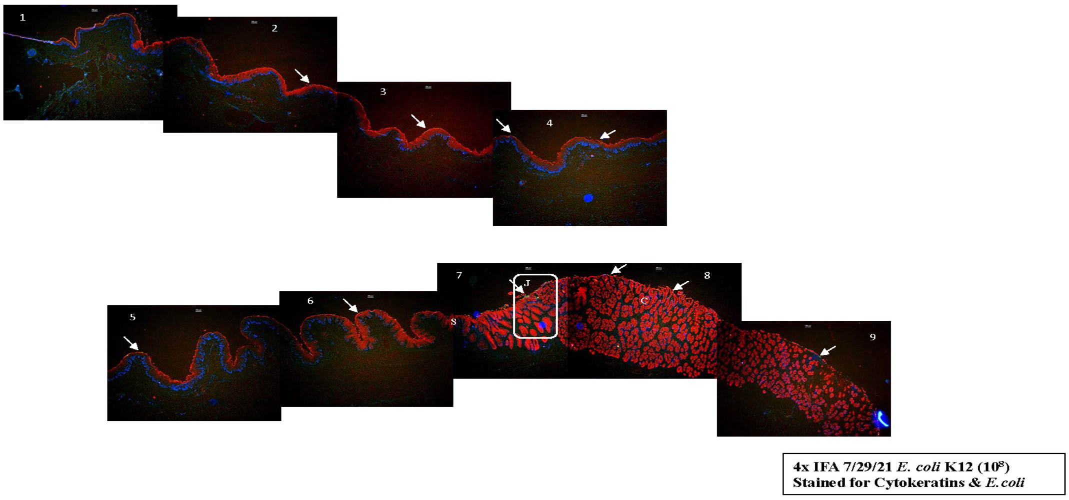

Figure S11. Stitched immunofluorescent images of a tissue section from a representative RAJ-IVOC inoculated with 10^8 CFU E. coli K12 in the comparative adherence assay. The tissue section was stained with antibodies targeting the RAJ cells and E. coli to demonstrate the presence of adherent E. coli K12. The immunofluorescent images were recorded at 40x magnification; the objective used is shown on the image-

label. The images were stitched in the order shown, panels 1-9, to provide a complete picture of the RAJ with adherent E. coli K12 indicated with arrows. The adherent E. coli K12, RAJ cells' cytokeratins, and the nuclei have green, orange-red and blue fluorescence, respectively. The squamous (S), junction (J, boxed), and columnar (C) regions of the RAJ are indicated.

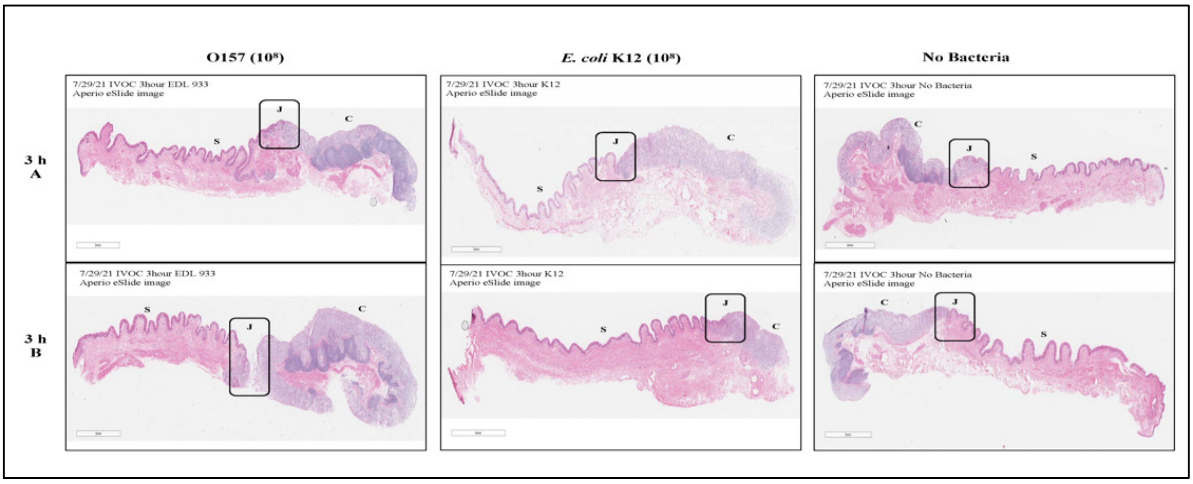

Figure S12. eImages of RAJ-IVOC tissue sections from a comparative adherence assay using the 10^8 CFU inoculum. The RAJ-IVOC were set in duplicates (A and B), inoculated with either O157 or E. coli K12 (10^8 CFU inoculum of each as shown in parenthesis), or not inoculated (no bacteria), and incubated at 39°C for 3 h. H&E-stained tissue section slides were scanned using the Aperio digital pathology system to obtain the eImages. The squamous (S), junction (J, boxed), and columnar (C) regions of the RAJ are indicated. A 3 mm scale bar is shown.

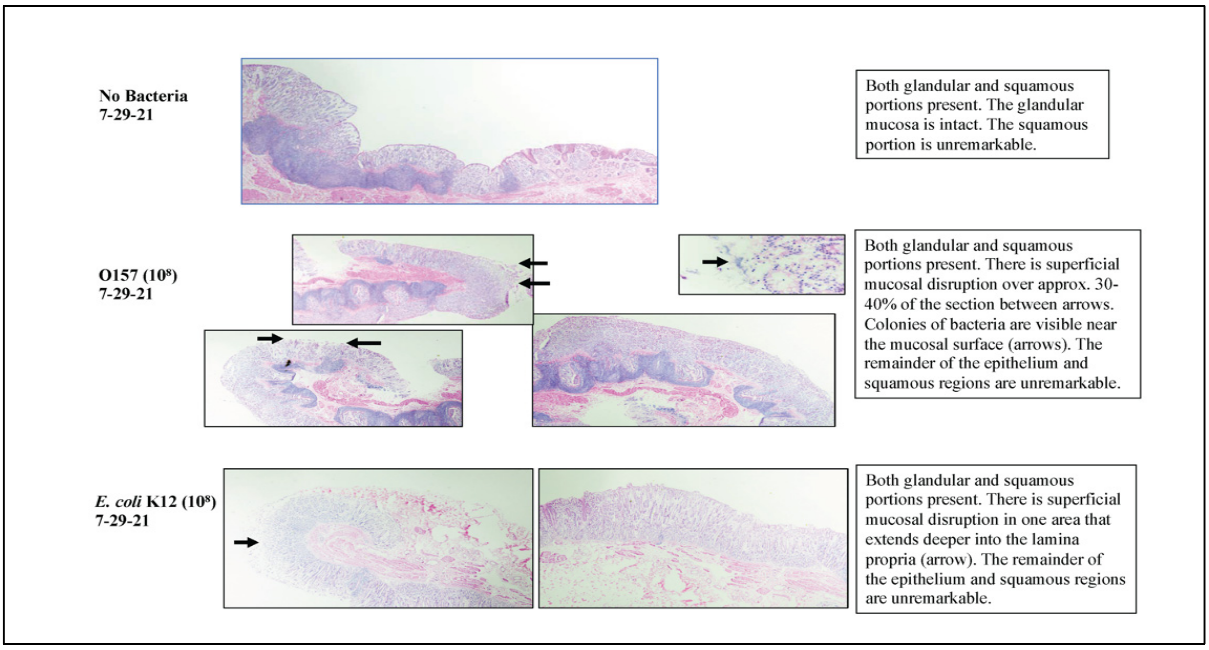

Figure S13. Representative histopathological report of a comparative adherence assay using the 10^8 CFU inoculum. The RAJ-IVOC were inoculated with either O157, or E. coli K12, or not inoculated (no bacteria),

and incubated at 39°C for 3 h. H&E-stained tissue section slides were scanned using the Aperio digital pathology system to obtain the eImages.

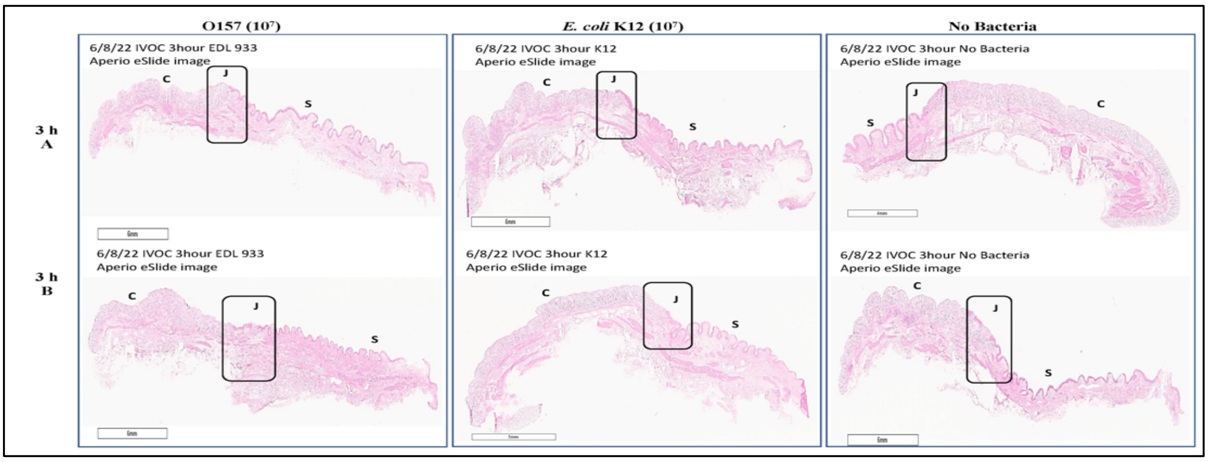

Figure S14. eImages of RAJ-IVOC tissue sections from a comparative adherence assay using the 10^7 CFU inoculum. The RAJ-IVOC were set in duplicates (A and B), inoculated with either O157, or E. coli K12 (10^7 CFU inoculum of each as shown in parenthesis), or not inoculated (no bacteria), and incubated at 39°C for 3 h. H&E-stained tissue section slides were scanned using the Aperio digital pathology system to obtain the eImages. The squamous (S), junction (J, boxed), and columnar (C) regions of the RAJ are indicated. A 3 mm scale bar is shown.

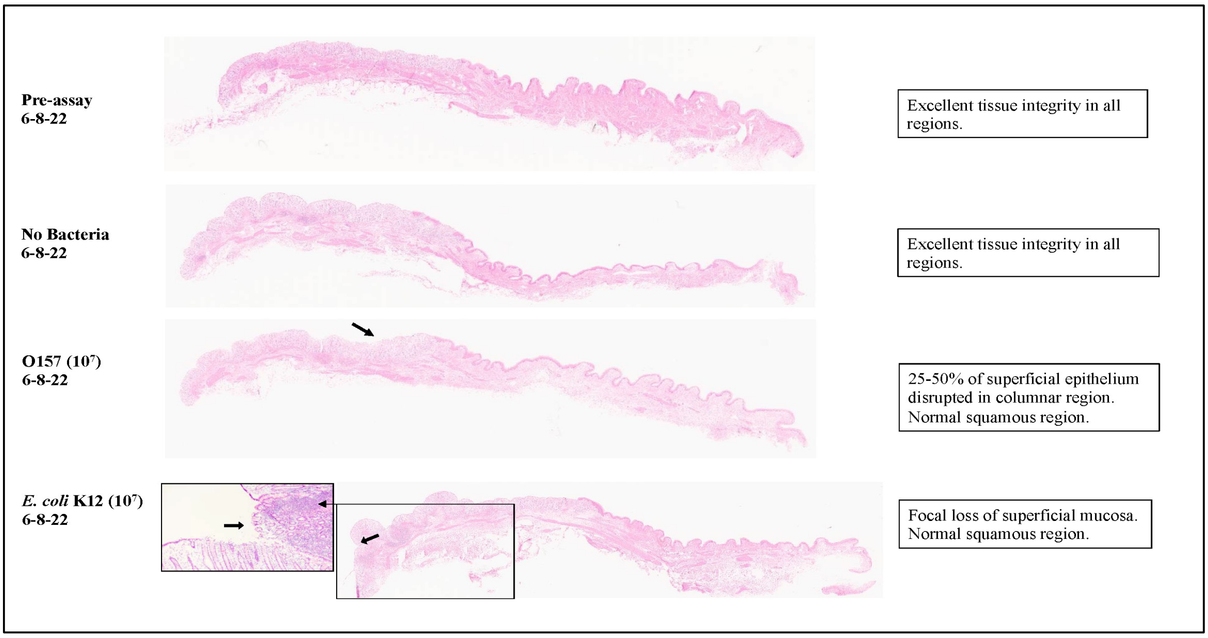

Figure S15. Representative histopathological report of a comparative adherence assay using the 10^7 CFU inoculum. The RAJ-IVOC were inoculated with either O157, or E. coli K12, or not inoculated (no bacteria), and incubated at 39°C for 3 h. H&E-stained tissue section slides were scanned using the Aperio digital pathology system to obtain the eImages.

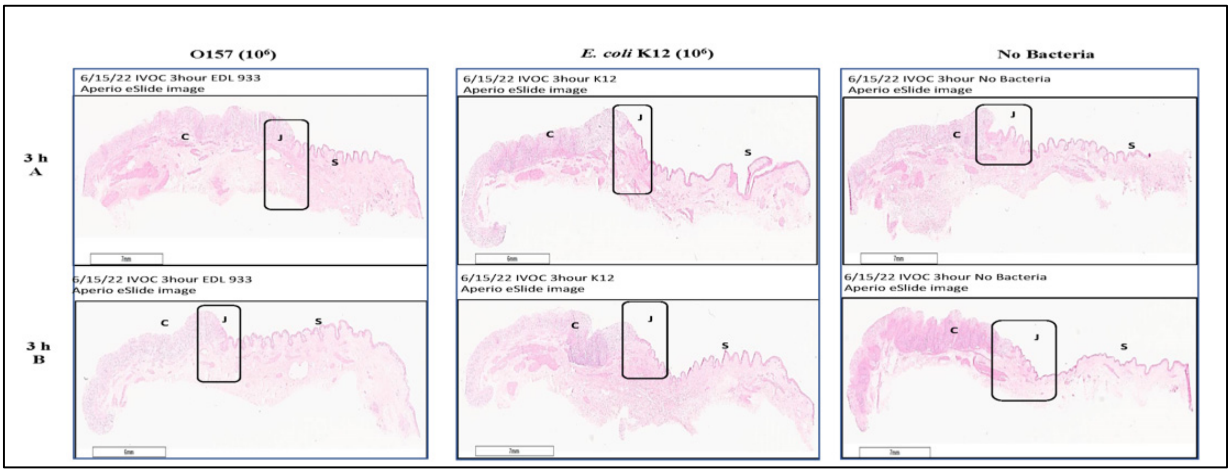

Figure S16. eImages of RAJ-IVOC tissue sections from a comparative adherence assay using the 10^6 CFU inoculum. The RAJ-IVOC were set in duplicates (A and B), inoculated with either \ O157, or E. coli K12 (10^6 CFU inoculum of each as shown in parenthesis), or not inoculated (no bacteria), and incubated at 39°C for 3 h. H&E-stained tissue section slides were scanned using the Aperio digital pathology system to obtain the eImages. The squamous (S), junction (J, boxed), and columnar (C) regions of the RAJ are indicated. A 3 mm scale bar is shown.

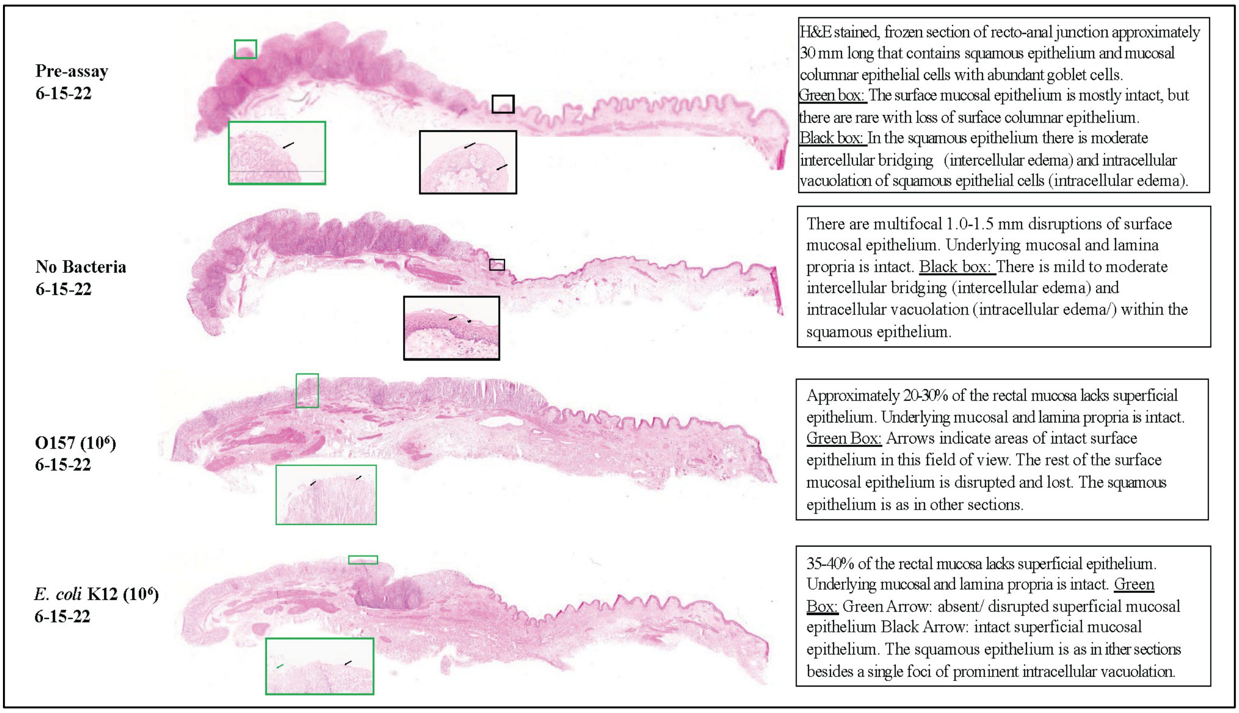

Figure S17. Representative histopathological report of a comparative adherence assay using the 10^6 CFU inoculum. The RAJ-IVOC were inoculated with either O157, or E. coli K12, or not inoculated (no bacteria), and incubated at 39°C for 3 h. H&E-stained tissue section slides were scanned using the Aperio digital pathology system to obtain the eImages.

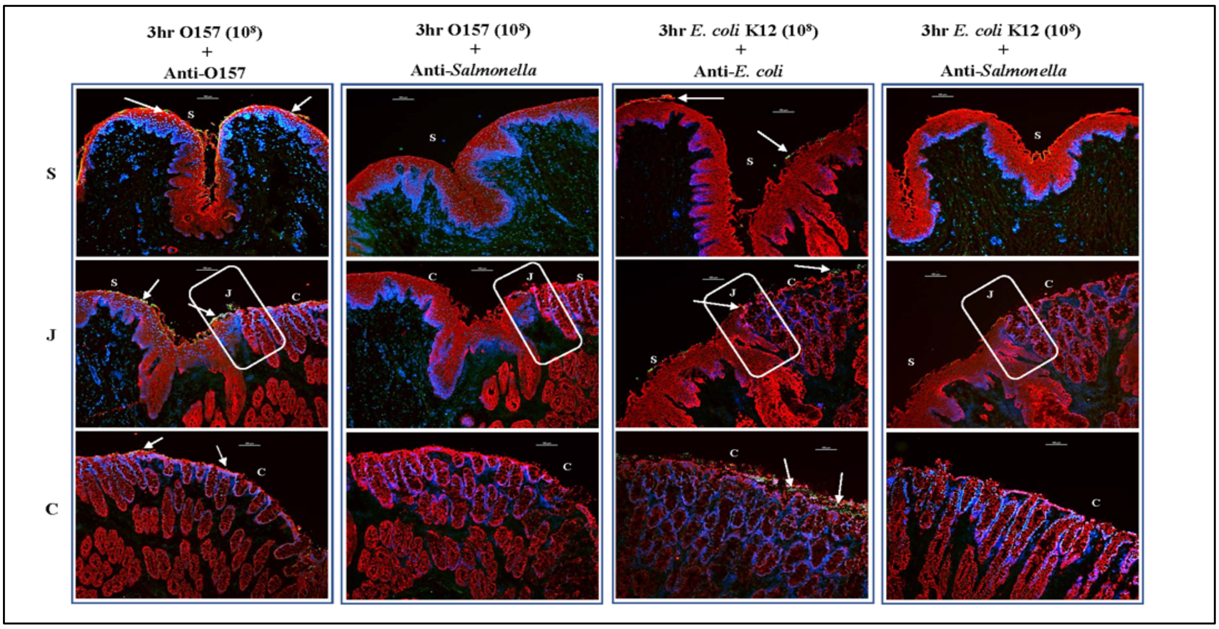

Figure S18. Immunofluorescent images of tissue sections from a representative RAJ-IVOC inoculated with 10^8 CFU O157 or E. coli K12 in a comparative adherence assay. The tissue sections were stained with antibodies targeting the RAJ cells and O157, E. coli or Salmonella to demonstrate the presence of adherent O157 or E. coli K12 and the absence of Salmonella, thereby also confirming specificity of antibodies used. The immunofluorescent images were recorded at 100x magnification. The adherent bacteria (shown with arrows), RAJ cells' cytokeratins, and the nuclei have green, orange-red and blue fluorescence, respectively. The squamous (S), junction (J, boxed), and columnar (C) regions of the RAJ are indicated.

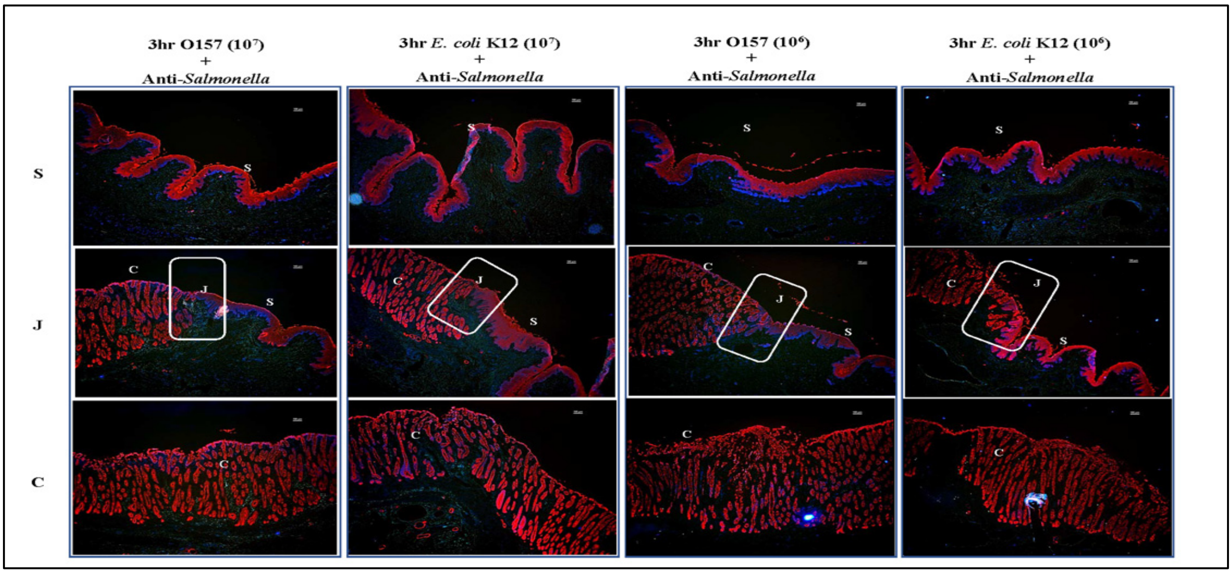

Figure S19. Immunofluorescent images of tissue sections from representative RAJ-IVOC inoculated with 10^7 or 10^6 CFU O157 or E. coli K12 in a comparative adherence assay. Only images of tissue sections stained with antibodies targeting Salmonella to demonstrate the absence of Salmonella and hence, the absence of any non-specific targeting by antibodies are shown. The immunofluorescent images were recorded at 100x

magnification. The RAJ cells' cytokeratins and the nuclei have orange-red and blue fluorescence, respectively. The squamous (S), junction (J, boxed), and columnar (C) regions of the RAJ are indicated.

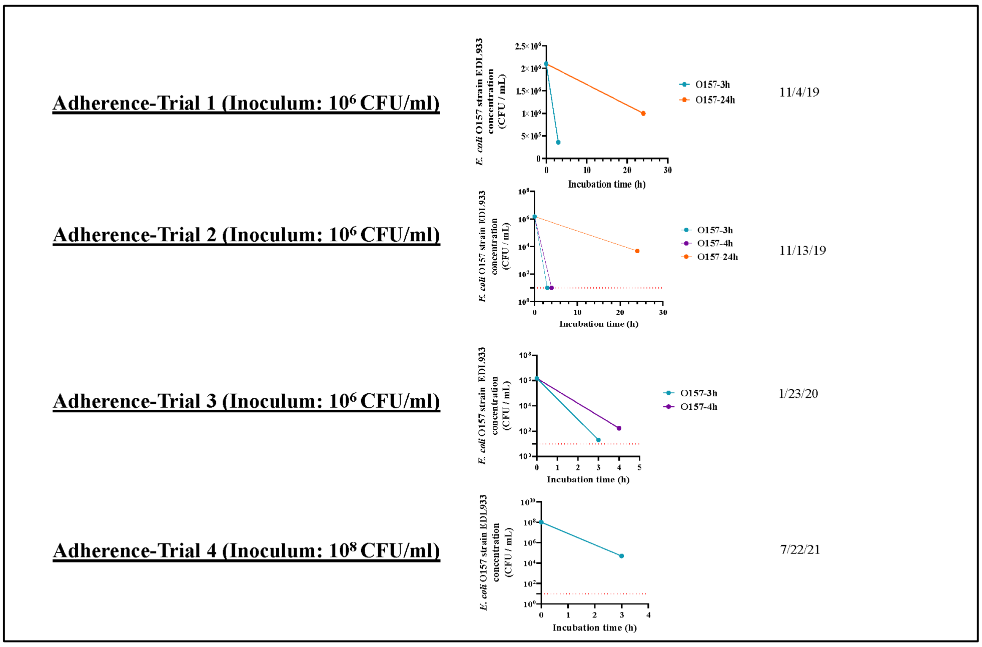

Figure S20A. Graphs representing O157 counts recovered from RAJ-IVOC tissues by culture in adherence trials 1 to 4.

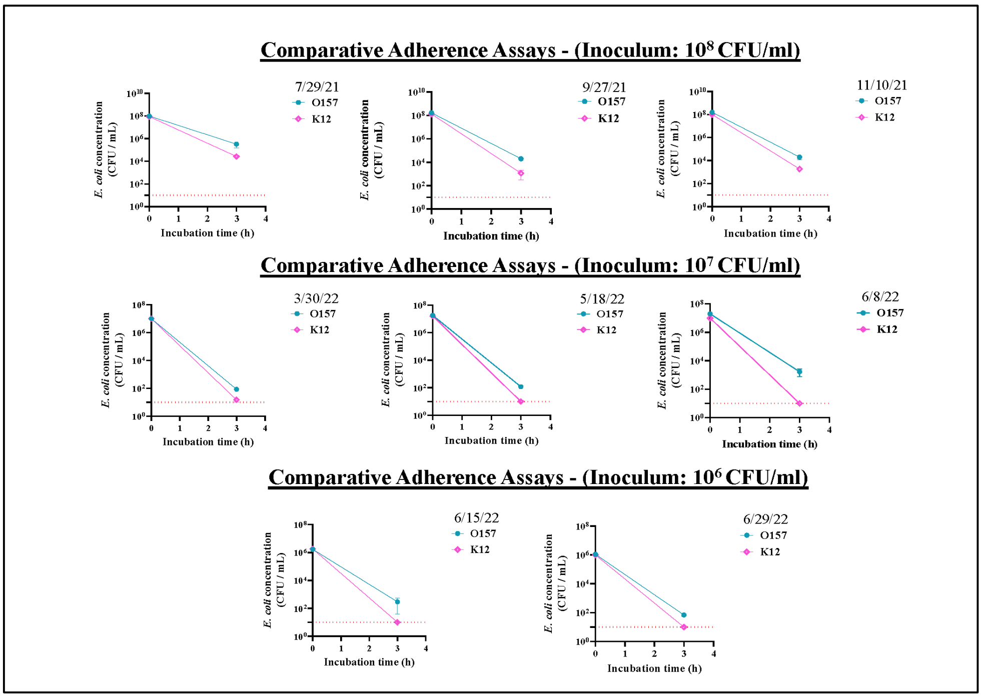

Figure S20B. Graphs representing E. coli (O157 and K12) counts recovered from RAJ-IVOC tissues by culture in comparative adherence assays conducted with different inoculum concentrations.

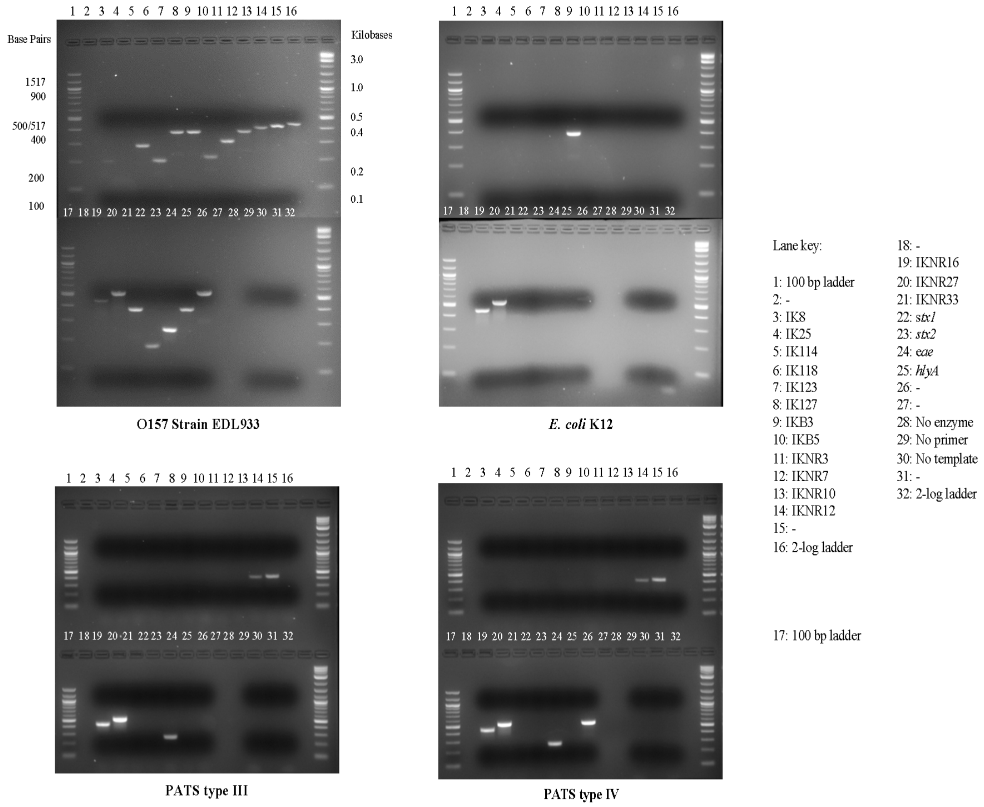

Figure S21. Electrophoretic patterns of amplicons comprising representative PATS profiles on 3% agarose gels.
